# Revision of the sophorolipid biosynthetic pathway in *Starmerella bombicola* based on new insights in the substrate profile of its lactone esterase

**DOI:** 10.1186/s13068-024-02533-1

**Published:** 2024-06-27

**Authors:** Zhoujian Diao, Sophie L. K. W. Roelants, Goedele Luyten, Jan Goeman, Isabel Vandenberghe, Gonzalez Van Driessche, Sofie L. De Maeseneire, Wim K. Soetaert, Bart Devreese

**Affiliations:** 1 Laboratory of Microbiology, Protein Research Unit, Department of Biochemistry and Microbiology, Faculty of Science, Ghent University, K. L. Ledeganckstraat 35, 9000 Ghent, Belgium; 2 Department of Biotechnology, Faculty of Bioscience Engineering, Centre for Industrial Biotechnology and Biocatalysis (InBio.Be), Ghent University, Coupure Links 653, 9000 Ghent, Belgium; 3 Bio Base Europe Pilot Plant, Rodenhuizenkaai 1, 9042 Ghent, Belgium; 4 R&D Department, AmphiStar, Zwijnaarde, Belgium; 5 Laboratory for Organic and Bioorganic Synthesis, Department of Organic Chemistry, Ghent University, Krijgslaan 281 (S.4), 9000 Ghent, Belgium

**Keywords:** Biosurfactants, Lipase, Serine hydrolase, *Starmerella bombicola* lactone esterase (SBLE), Bola sophorolipids, Lactonic sophorolipids, Transesterification, Mass spectrometry

## Abstract

**Background:**

Sophorolipids (SLs) are a class of natural, biodegradable surfactants that found their way as ingredients for environment friendly cleaning products, cosmetics and nanotechnological applications. Large-scale production relies on fermentations using the yeast *Starmerella bombicola* that naturally produces high titers of SLs from renewable resources. The resulting product is typically an extracellular mixture of acidic and lactonic congeners. Previously, we identified an esterase, termed *Starmerella bombicola* lactone esterase (SBLE), believed to act as an extracellular reverse lactonase to directly use acidic SLs as substrate.

**Results:**

We here show based on newly available pure substrates, HPLC and mass spectrometric analysis, that the actual substrates of SBLE are in fact bola SLs, revealing that SBLE actually catalyzes an intramolecular transesterification reaction. Bola SLs contain a second sophorose attached to the fatty acyl group that acts as a leaving group during lactonization.

**Conclusions:**

The biosynthetic function by which the *Starmerella bombicola* ‘lactone esterase’ converts acidic SLs into lactonic SLs should be revised to a ‘transesterase’ where bola SL are the true intermediate. This insights paves the way for alternative engineering strategies to develop designer surfactants.

**Supplementary Information:**

The online version contains supplementary material available at 10.1186/s13068-024-02533-1.

## Background

Sophorolipids (SLs) belong to the glycolipid biosurfactant family that have been applied in a variety of fields, such as household products, personal care, cosmetics and agriculture, attributing to their unique characteristics, including low toxicity, biodegradability and antimicrobial activity [1–5]. SLs are secondary metabolites produced by a number of non-conventional yeasts, most of which are members of the *Starmerella* clade. *Starmerella* strains naturally produce SLs as a mixture comprising components with two different molecular conformations, viz. an open acidic structure and a closed lactonized structure [5]. The structural variation affects their physicochemical and biological properties. Among them, the better water solubility and foaming performance pertain to acidic SLs, while the lactone form shows better antimicrobial activity and surface tension lowering ability [6].

**Fig. 1:**
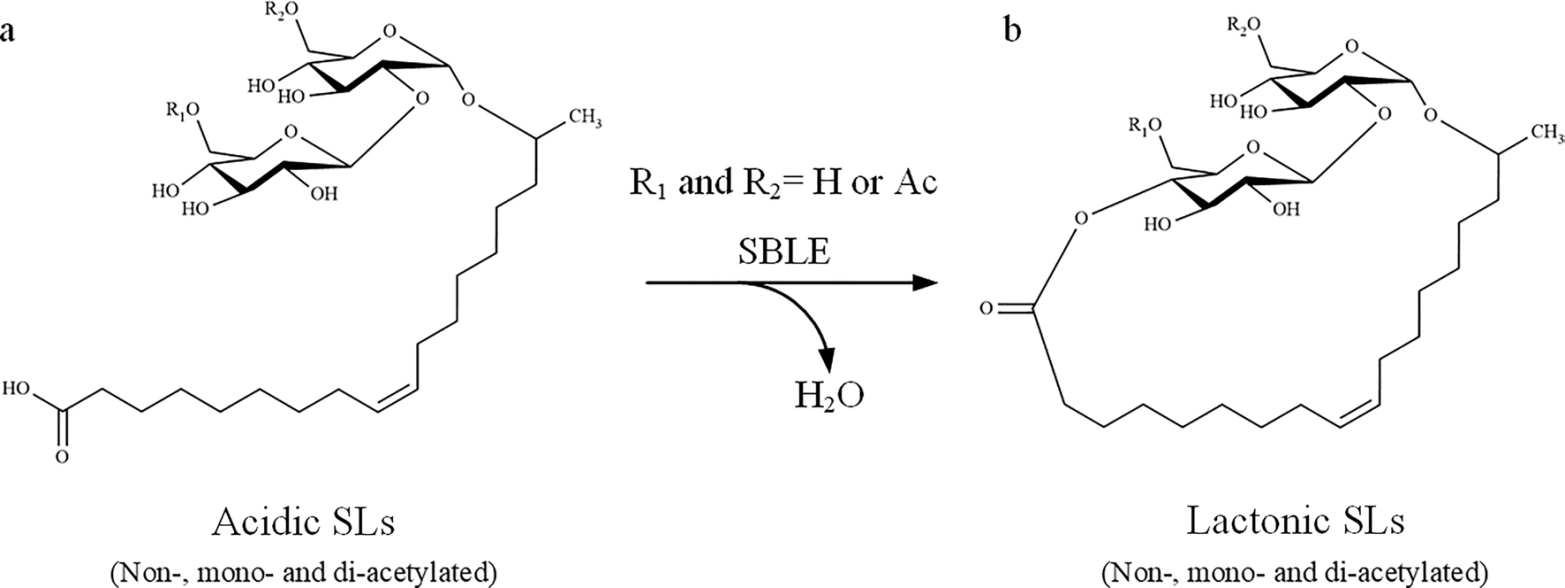
Schematic diagram of the reaction believed to be catalyzed by SBLE, i.e., conversion of acidic SLs to form lactonic SLs

Most studies on SLs biosynthesis were performed in *Starmerella bombicola* in view of its high productivity [7, 8]. These investigations led to the discovery of a biosynthetic gene cluster (BGC) and allowed to propose a biosynthetic pathway. This BGC encodes a cytochrome P450, two glycosyltransferases and an acetyltransferase gene, explaining the formation of an *ω*- or (*ω*-1) hydroxy fatty acid, consecutive addition of two glucose moieties that then can be acetylated to form acidic SLs [9–15]. Later, an enzyme designated as *Starmerella bombicola* lactone esterase (SBLE) was identified and believed to be responsible for the last step of the biosynthetic pathway, that is, catalyzing intramolecular esterification (lactonization) of acidic SLs to form their lactone congeners (Fig. 1) [13, 15].

SBLE was discovered in the extracellular medium of *S. bombicola* cultures by an extracellular proteomics analysis. This enzyme was described as a novel member of the lipase A family (CALA), of which the *Moesziomyces antarcticus* (*Pseudozyma antarctica*, formerly *Candida/Pseudozyma antarctica*) enzyme CALA is the best characterized and industrially available as NovoCor AD L [13]. The capacity of SBLE to catalyze the acidic SLs lactonization in the aqueous environment, was deemed to be extremely uncommon. In general, lipases perform esterification reactions only in an anhydrous environment [16] and only a few enzymes are reported to catalyze this sort of reactions in water-rich media [17–19]. Furthermore, enzymatic synthesis of macromolecular lactones (chain length higher than 12 C-atoms) was considered to be difficult and entropically unfavorable, notably in aqueous phase [20]. Currently, lipase B (CALB), another lipase from *M. antarcticus*, is the only lipase that has been documented to lactonize non-acetylated acidic SLs in vitro, but this is only obtained in an organic solvent, i.e., dry tetrahydrofuran (THF) and results in non-natural congeners [21, 22]. In contrast to CALB, CALA—as a homolog of SBLE—has not been reported to possess lactonization activity towards acidic SLs hitherto. Yet, CALA was demonstrated to prefer esterification reactions over hydrolysis [23] and, alike SBLE, has capacities for catalyzing condensation reactions in a water-rich media [24, 25]. Curiously, CALA still retains hydrolytic activity, e.g., for triglycerides [26], whereas no hydrolytic activity was detected in vitro for SBLE [15].

**Fig. 2:**
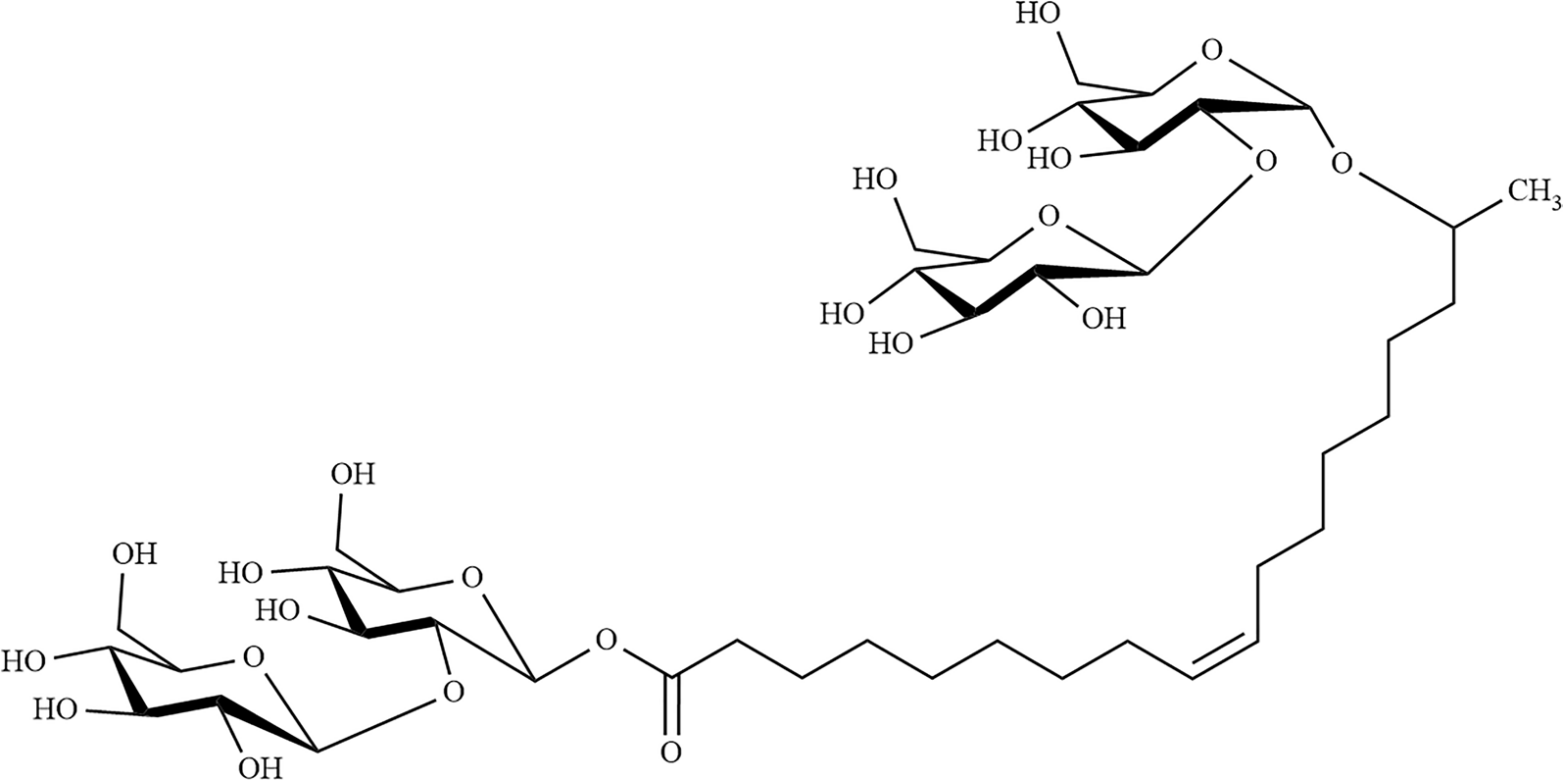
Chemical structure of non-acetylated bolaform SLs

SBLE was recombinantly produced in *P. pastoris* [15, 27]. This rSBLE was used to assess catalytic activity and to determine specificity. The substrate for these studies was always believed to contain exclusively acidic SLs as it was produced using an engineered strain deficient in SBLE, which was believed to convert acidic SLs into lactonic SLs. Hence, based on these studies, SBLE was described to only accept acetylated (mono-/di-) precursors of acidic SL in vitro and to act as a reverse lactonase. The presence of another type of sophorolipids present in minute amounts in extracellular glycolipids produced by *S. bombicola* was first described by Price et al. [28] and had not been identified before due to the low amounts, combined with sensitivity of analytical equipment. A double knock-out *S. bombicola* strain, i.e., a strain deficient in SBLE and in the acetyltransferase was found to produce an SLs mixture that not only contains acidic (open) SLs, but also increased amounts of these bola SLs (Fig. 2) [29, 30].

Van Bogaert, Buyst [29] did not observe formation of bola sophorolipids from the single deletion Δ*sble* strain, assuming that additional deletion of the acetyltransferase was required to form bola SLs. However, during recent investigations in bioreactor experiments with the Δsble strain which are described in an accompanying paper [31], it was observed that actually also in this strain, elevated amounts of bola SLs were detected in contrast to what had been described by Van Bogaert, Buyst [29]. This coincided with novel studies on the rSBLE activity in our laboratory with better defined and purified SL samples that recently became available through the continuous engineering and optimization attempts with *S. bombicola*. This work enabled us to shed new insights on the actual activity of SBLE and we surprisingly show that the actual SBLE substrates are in fact bola SLs indicating that SBLE catalyzes an intramolecular transesterification reaction to synthesize lactonic SLs from bola SLs rather than lactonization of acidic SLs as described previously [13, 15].

## Materials and methods

### Materials

Most chemicals were purchased from Chem-Lab ((Zedelgem, Belgium); methanol, ethanol, glycerol, sodium phosphate, sodium chloride, imidazole) and Sigma-Aldrich ((New Jersey, U.S.); reduced glutathione, magnesium sulfate, Tris–HCl, sodium citrate, trifluoroacetic acid and α-cyano-4-hydroxycinnamic acid), unless indicated otherwise. Sophorolipids were produced by the authors, some were obtained from Amphistar NV.

### Production of recombinant SBLE

For recombinant SBLE (rSBLE) production, a *HAC1* co-expressing strain of *P. pastoris* (syn. *Komagataella phaffii*) NRRL-Y-11430 [32] transformed with the pPICZαB_rSBLEopt construct which harbors the highest yield of rSBLE described in De Waele, Vandenberghe [27], was utilized in the research. The strain was grown in buffered glycerol-complex medium (BMGY) in 3-L baffled shake flasks containing 500 ml medium for 48 h at 28 °C, 250 rpm. Then, the induction of gene expression was performed in buffered-methanol complex (BMMY) medium for 48 h at 16 °C, 250 rpm. Every 12 h, 1% methanol was added for continuous stimulation of protein production. Both BMGY and BMMY consist of 1% (w/v) yeast extract (Lab M), 2% (w/v) peptone (BD), 100 mM phosphate buffer at pH 6.0 and 1.34% (w/v) yeast nitrogen base (YNB, Formedium) with 1% (v/v) glycerol or 1% (v/v) methanol as sole carbon source, respectively. Finally, the culture containing the produced rSBLE was centrifuged (5000*g*, 10 min) to collect the supernatant for protein purification.

### Purification of recombinant SBLE

For purification of rSBLE, a two-step purification strategy was utilized by following the protocol described in De Waele, Vandenberghe [27]. 0.01% (w/v) reduced glutathione and 2 mM (final concentration) of magnesium sulfate were added in the supernatant, after which the pH was adjusted to 7.5. After filtering the sample through a Steritop® Filter Unit (EMD Millipore) or VacuCap® (VWR) with a pore size of 0.22 µm, the filtrate was subsequently loaded on a HisTrap™ HP column (5 ml, Cytiva) previously equilibrated with binding buffer of 50 mM Na_2_HPO_4_, pH 7.5, 500 mM NaCl at a flow rate of 5 ml/min. Purification was done on an ÄKTA Purifier system (GE Healthcare). Following sample loading, the column was washed with binding buffer until the UV (280 nm) absorbance reached a steady baseline. Then, a step-wise elution was performed using 20- and 200 mM, respectively, imidazole in binding buffer. The two eluted fractions were mixed and immediately desalted via a buffer exchange using 25 mM Tris–HCl, pH 7.5, 150 mM NaCl and Amicon® Ultra-15 centrifugal filter devices (Merck) with a 10-kDa cut-off, and concentrated to 1 ml eventually. In the second step, the 1 ml concentrated IMAC fraction was injected onto a HiLoad® 16/600 Superdex® 200 pg column (GE Healthcare) equilibrated with the desalting buffer (25 mM Tris–HCl, pH 7.5, 150 mM NaCl) and eluted with the same buffer. The fractions containing rSBLE were concentrated to 1.0 ml using Amicon® Ultra-15 centrifugal filter devices (Merck) with a 10 kDa cut-off. The concentration of rSBLE was determined using the Thermo Scientific™ Coomassie (Bradford) Protein Assay Kit and using the Bio-Rad Microplate Reader model 680 (595 nm). SDS-PAGE analysis confirmed the purity of the rSBLE enzyme (Supplementary Figure S1). The protein was stored at -80℃ for further experiments.

### HPLC-based assay for sophorolipid analysis

An HPLC-based activity assay was followed as described in De Waele, Vandenberghe [27] with some adaptations. In brief, 10 μg of purified rSBLE was added to 500 μl of reaction buffer, containing 5 mM of substrate and 50 mM sodium citrate at pH 3.5. The mixture was incubated for 2 h at 30℃ and 1400 rpm after which the reaction was stopped using 1500 μl 100% (v/v) ethanol. The reaction in which rSBLE was replaced by the same amount of buffer (25 mM Tris, 150 mM NaCl, pH7.5) used for protein purification was prepared as negative control in the assay. After concentrating the sample using a SpeedVac vacuum centrifuge (Thermo Savant, Holbrook, NY) to 250 μl, 100 μl of the samples were analyzed by reverse phase HPLC using an Ettan™ LC system (GE Healthcare) implementing a Brownlee Spheri-5 RP-18 Cartridge Column-220 × 2.1 mm (Perkin Elmer) using a gradient separation with UV absorption detection (207 nm). The gradient started at 30% acetonitrile (ACN) and linearly increased to 50% in 15 min, after which the gradient increased linearly from 50% ACN until 80% in 30 min. The elution solvent was kept at 80% ACN for 5 min and was then returned to 30% ACN in 1 min. A flow rate of 0.15 mL/min was applied. For those peaks that significantly differed in the chromatograms of samples before and after reaction, fractions were collected for MALDI-TOF MS. Note that this method is slightly different from our previous work [27] and although HPLC profiles look similar, retention times cannot be directly compared.

### MALDI-TOF MS analysis of sophorolipids

Fractions collected from HPLC were dried using a SpeedVac vacuum centrifuge (Thermo Savant, Holbrook, NY) and the dried compounds were then resuspended in 12 µL of 50% ACN/0.1% trifluoroacetic acid (TFA) solution. 1 µL of resuspended compound, mixed with a saturated α-cyano-4-hydroxycinnamic acid solution in a 1:1 ratio was spotted onto an Opti-TOF 384 Well MALDI Plate Insert for MALDI-TOF MS analysis with the MALDI-TOF/TOF 4800 Plus (ABSciex).

### LC–MS analysis of sophorolipids

To confirm the conversion of bola SLs to lactonic SLs by rSBLE, LC–MS analysis was performed. SL samples (dissolved to 1 mg/ml in ethanol) were separated on an Agilent 1100 series HPLC equipped with a quaternary pump and diode array detector, using a Phenomenex Kinetex C18 150 × 4.6 mm 5µ solid core type column at 35 °C, flow rate 1.5 ml/min. A gradient ranging from 20 to 80% acetonitrile (ACN) in 30 min with 0.1% formic acid (HCOOH) was used to separate the products. The HPLC system was coupled to an Agilent G1956B single quadrupole MS detector equipped with an electrospray ionization (ESI) source. The mass spectrometer was set to scan the mass-to-charge (m/z) range of 600–1200 amu in negative ion mode.

### HPLC analysis of sophorose

Products from enzymatic conversion of sophorolipids were analyzed by ultra performance liquid chromatography (UPLC) on an Acquity H-Class UPLC system (Waters) coupled with an Acquity Evaporative Light Scattering Detector (ELSD; Waters) in combination with an Acquity UPLC BEH amide column (130 Ä, 1.7 μm, 2.1 × 100 mm; Waters). The column was kept at 55 °C and samples were analyzed at a flow rate of 0.5 mL/min for 8 min/sample.

A binary gradient elution system was applied, consisting of 1% triethyl amine in milli-Q water (eluent A) and 100% acetonitrile (eluent B). The gradient profile was as follows: during the first 3 min, the concentration of eluent A increases from 10 to 25%, which is kept at this level for 0.5 min. Next, the concentration of eluent A decreases again to 10% in 1 min. and is maintained at this level for the remaining 3.5 min. of the method.

## Results

### Activity assay of rSBLE on pure acidic SLs

In previous studies, the activity of SBLE was measured using a SLs mixture isolated from a strain deficient in SBLE. This SLs mixture was supposed to contain acidic SLs only. The recent finding of bola SLs in ∆*sble* cultures [29] raised questions about this. Therefore, activity tests were now performed on different SL samples of which the composition was better defined by improved analytical methods.

First, the in vitro activity of recombinantly produced rSBLE for the lactonization using three different samples of purified acidic SLs was tested. The samples used were largely enriched for (1) di-acetylated acidic SLs (C_18:1_, ω), (2) di-acetylated acidic SLs (C_18:1_, ω-1), and (3) non-acetylated acidic SLs (C_18:1_, ω-1) (Table 1).

**Table 1:** Used acidic sophorolipids (SLs), their purity and composition (Ac: acetyl)

Product	Di-Ac, C_18:1_, terminal (ω)	Di-Ac, C_18:1_, subterminal (ω-1)	Non-Ac, C_18:1_, subterminal (ω-1)
Code	INV-103/104	INV-115	INV-91
Purity	95.3%	96.4%	93.1%
Other minor components	Mono-Ac, C_18:1_, terminal (ω): 2.6% non-Ac, C_18:1_, terminal (ω): 1.9% Others: 0.2%	Mono-Ac, C_18:1_, subterminal (ω-1): 1.8% Others: 1.8%	Non-Ac, C_18:0_, subterminal (ω-1): 2.9% Others: 3.9%
Molecular mass (amu)	706.4	706.4	622.4

**Fig. 3:**
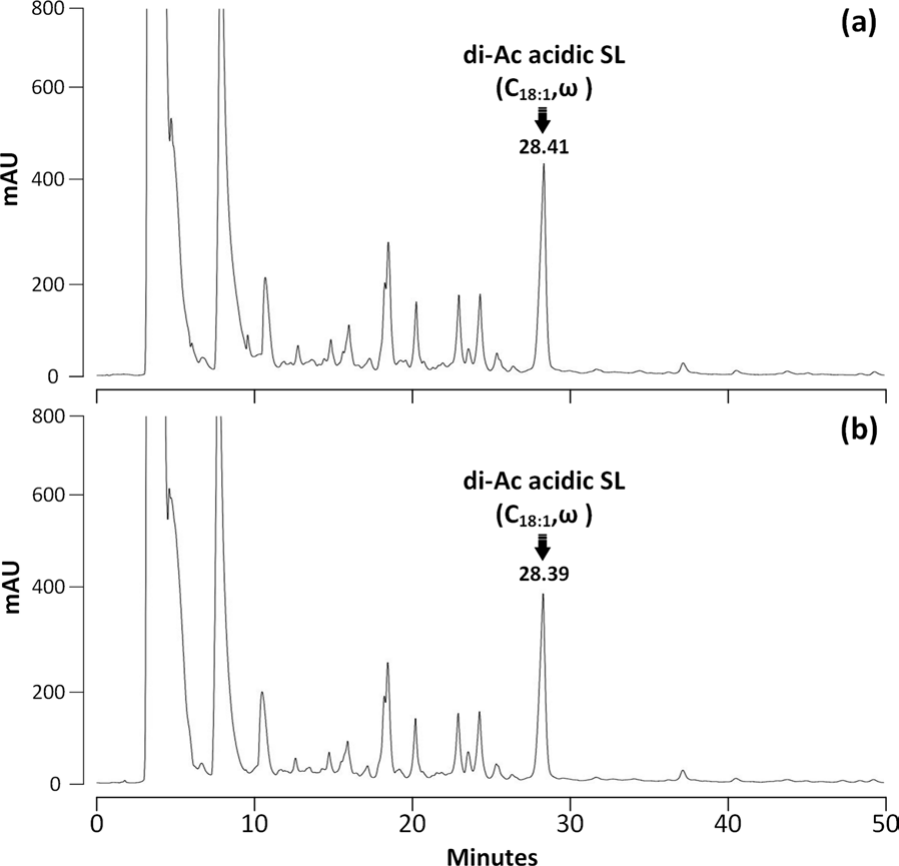
HPLC–UV chromatograms of samples obtained from the activity assay performed on di-acetylated acidic SLs with terminal (*ω*) hydroxylation. **a** Negative control (without addition of enzyme). The marked peak refers to the di-acetylated C_18:1_ acidic SL. **b** Reaction mixture obtained after incubation di-acetylated acidic SLs with rSBLE

**Fig. 4:**
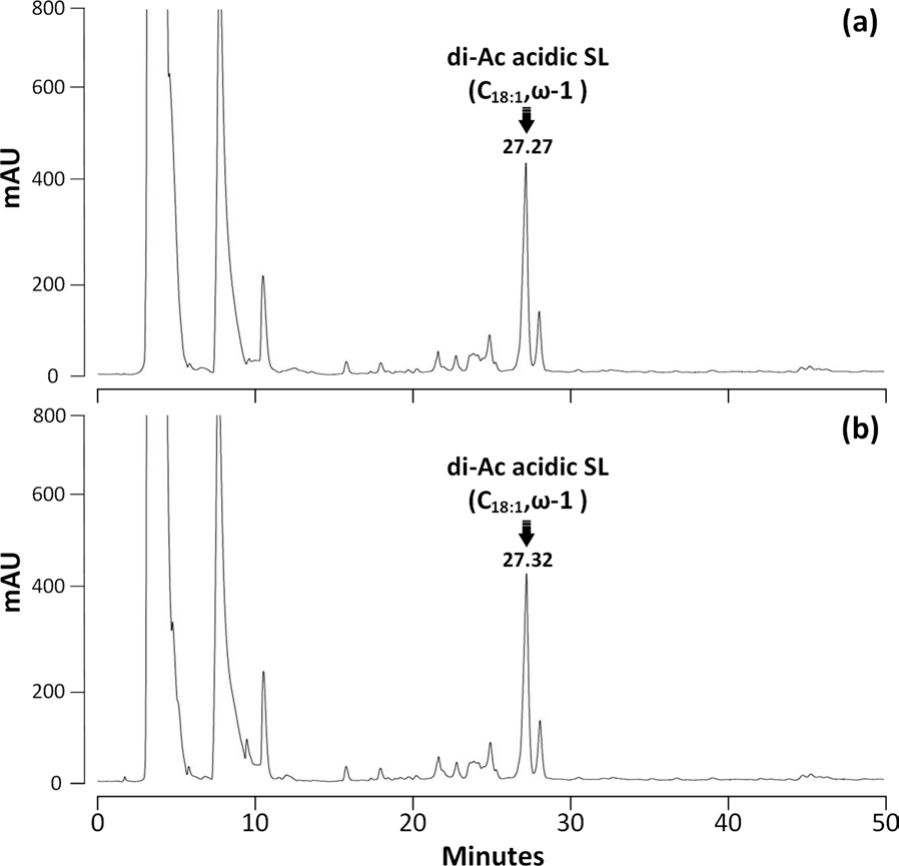
HPLC–UV chromatograms of samples obtained from the activity assay using di-acetylated acidic SLs with attachment of the glycosyl group mainly at the subterminal (*ω*-1) position. **a** Negative control of this experiment (without addition of enzyme). The marked peak refers to the di-acetylated C_18:1_ acidic SL. **b** Reaction mixture obtained after incubation di-acetylated acidic SLs with rSBLE

The activity of rSBLE was analyzed using an HPLC-based assay. A negative control experiment was performed, which involved analysis of the reaction mixture without the addition of enzyme. The results showed that no lactonic SLs were detected after reaction of any of the three acidic SLs (Figs. 3, 4and5). Indeed, lactonic SLs are more hydrophobic and elute later than acidic SLs (see further). In none of the chromatograms such peaks were observed. In fact, the chromatograms of samples before and after incubation with rSBLE do not display any significant change in pattern.

**Fig. 5:**
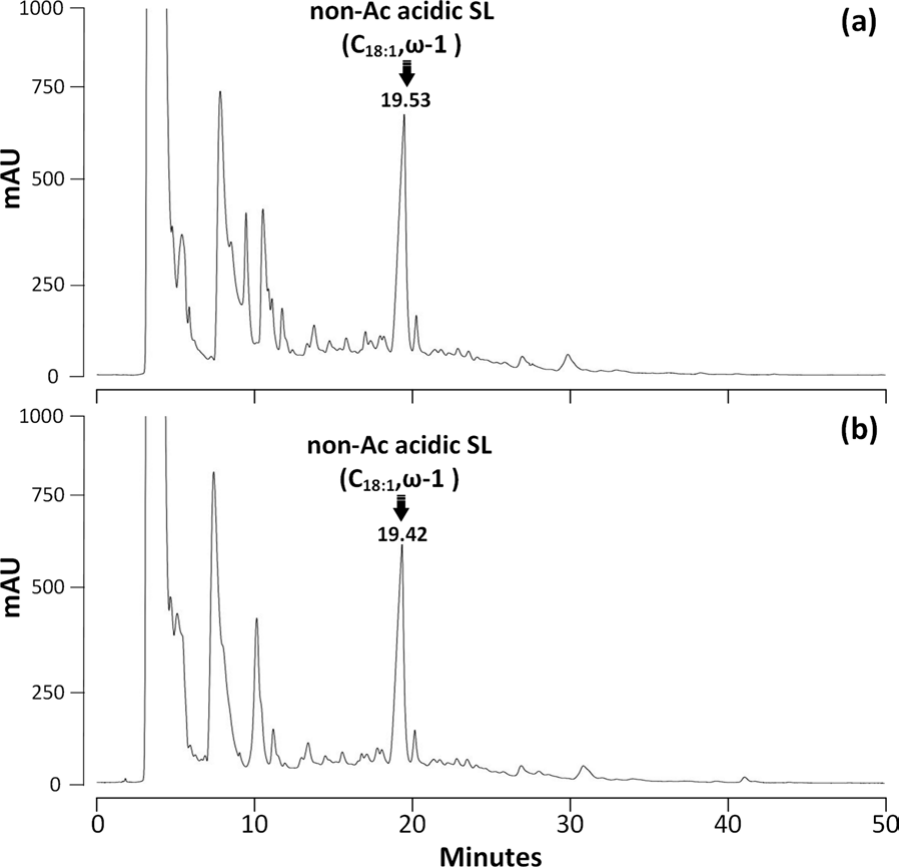
HPLC–UV chromatograms of samples obtained from the activity assay of rSBLE using non-acetylated acidic SLs with attachment of glycosyl group mainly at subterminal (*ω*-1) position. **a** Negative control of this sample (without addition of enzyme). The marked peak refers to the non-acetylated C_18:1_ acidic SL. **b** Reaction mixture obtained after incubation non-acetylated acidic SLs with rSBLE

Additionally, all the samples were analyzed using MALDI-TOF MS to check for possible formation of SL polymers. However, inspection of the mass spectra did not provide any indication that polymerization occurred in the reaction mixtures (data not shown).

**Fig. 6:**
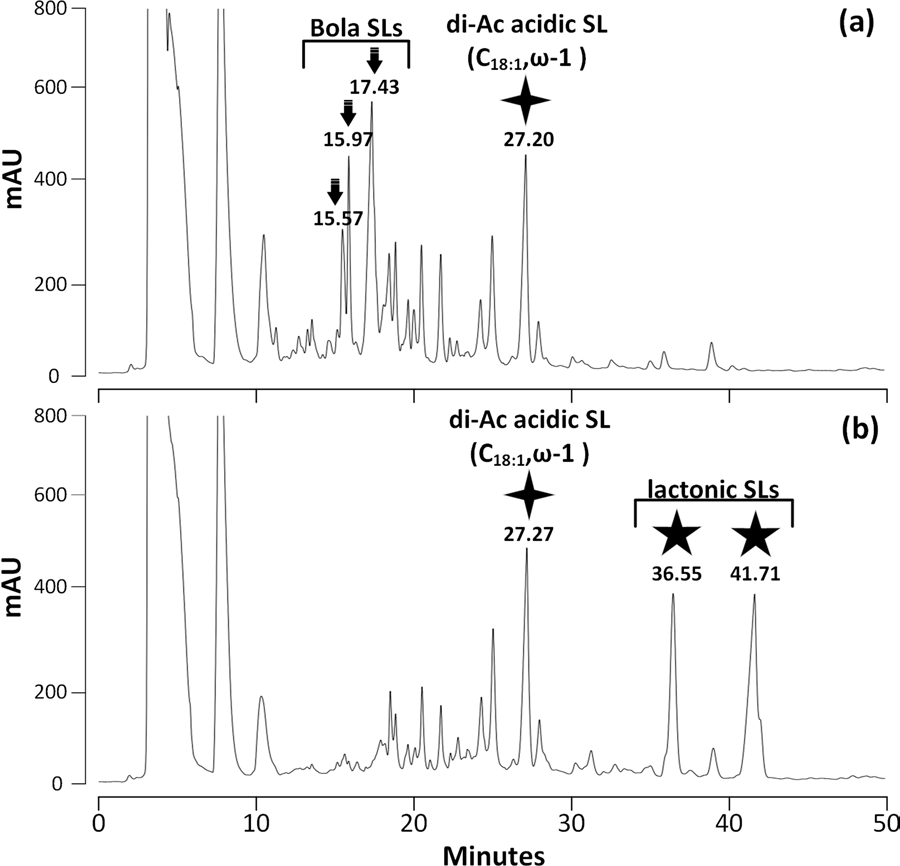
HPLC–UV chromatograms of samples from **a** the negative control of an SL mixture obtained from the Δsble strain used for the activity assay, **b** the activity assay of rSBLE using this SL mixture shown as substrate. The five-angle stars in panel **b** indicate the produced lactonic SLs after incubation. The arrows in panel **a** indicate the three peaks with significant decrease after reaction with the addition of the rSBLE enzyme (shown in panel **b**), black four-angle stars indicate the peak corresponding to di-acetylated acidic SLs (C_18:1_) with subterminal (*ω*-1) attachment, of which no reduction is observed upon addition of rSBLE

These data surprisingly indicate that rSBLE is not able to convert any of the three provided acidic SLs into lactonic SL in vitro. We returned therefore to an activity test using the original crude SL mixture that was used by Ciesielska, Roelants [15]. This mixture was obtained from an extract from the ∆*sble* strain described by Ciesielska, Van Bogaert [13] without further purification. This crude SLs mixture was at that time considered to only contain acidic SLs. However, when comparing the HPLC chromatogram of this ‘old’ SL mixture with the new samples of enriched acidic SLs, it became clear that this sample does not only contain di-acetylated acidic SLs (retention time 27.2 min) as expected. Indeed, additional peaks/compounds are observed with retention 15.6 min, 16.0 min and 17.4 min, (indicated with arrows) (Fig. 6a).

MALDI-TOF MS was performed to investigate the identity of these compounds. The observed molecular masses of these compound all correspond to the calculated mass of bola SLs, previously described by Van Bogaert et al. [29]. Indeed, the mass spectrum displays a compound with m/z of 1011.5 (for the peak at RT of 15.6 min, Table 2, Supplementary Fig. S2a), which corresponds to the Na^+^-adduct of mono-acetylated bola SL (C_18:1_). Also, di-acetylated (C_18:2_) and di-acetylated (C_18:1_) bola SLs were detected with MW, after subtraction of the mass of the Na^+^ ion, of 1028.5 and 1030.5 at RTs of 16.0 min and 17.4 min, respectively (Table 2, Supplementary Fig. S2b, c).

**Table 2:** Overview of bola SLs in the reaction mixture that displayed the largest decrease in intensity after incubation with rSBLE as shown in Fig. 6 (Ac: acetyl)

Retention (RT, min)	Molecular mass	Type of bola SL
15.6	988.5	Mono-Ac bola SL, C_18:1_
16.0	1028.4	Di-Ac bola SL, C_18:2_
17.4	1030.4	Di-Ac bola SL, C_18:1_

Additional confirmation of the identity of these components comes from MS/MS analysis included in supplementary Fig S3. This striking observation indicated thus that the sophorolipid mixture produced by the SBLE deficient *S. bombicola* strain does not only contain acidic SLs, but also bola SLs. More curious, exactly these components disappear after incubation of this sample with rSBLE giving rise to the formation of di-acetylated lactonic SLs (Fig. 6b), whereas peaks corresponding to acidic SLs hardly diminish in intensity, see for example the peak corresponding to di-acetylated SL at 27.7 min marked with a four-angle star.

### In vitro activity assays of rSBLE using bola SLs

Based on the observation that upon incubating SBLE with a mixture of bola SLs and acidic SLs, lactonized SLs are obtained, but not with acidic SLs alone, it became obvious that bola SLs might be the actual substrates of the SBLE enzyme. In order to confirm this, two types of bola SLs (Table 3) were used for an additional assay of which the main compounds are: (1) di-acetylated bola SLs and di-acetylated acidic SLs in an approximately 1:1 ratio (code: INV-113) and (2) non-acetylated (and minor amounts of mono-acetylated) bola SLs (code: INV-22).

**Table 3:** Used bola SLs substrates, main components and corresponding proportions (Ac: acetyl)

Product	Bola SLs sample 1	Bola SLs sample 2
Code	INV-113	INV-22
Composition	Di-Ac bola SLs: 41.6% Di-Ac acidic SLs: 43.2% Tri-Ac bola SLs: 5.2% Others: 10.0%	Non-Ac bola SLs: 72.6% Mono-Ac bola SLs: 25.3% Di-Ac bola SLs: 0.7% Non-Ac acidic SLs: 1.4%

**Fig. 7:**
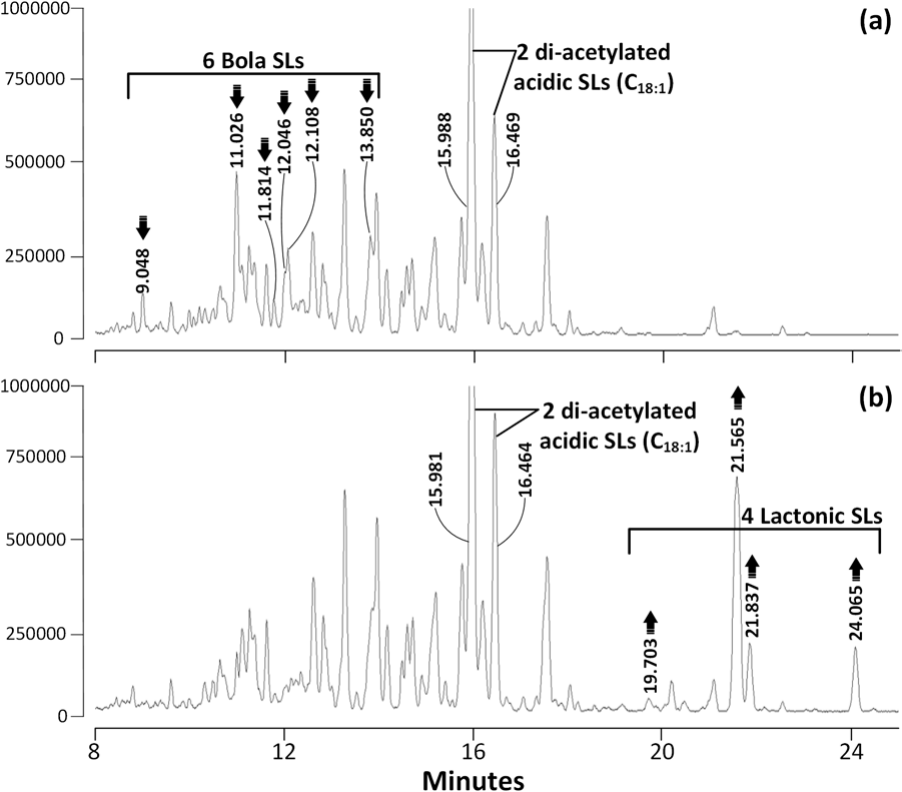
TIC chromatograms from an LC–MS-based activity assay of rSBLE using a bola SLs sample containing mainly acetylated congeners as substrate (code: INV-113). The top chromatogram (**a**) displays the mixture to which no enzyme is added. Peaks indicated with an arrow are mono- to tetra-acetylated bola SLs (see Table 4) that decreased in intensity after incubation with the enzyme. The lower chromatogram (**b**) displays the product of this mixture after incubation with rSBLE. All indicated peaks are lactonic SLs (Table 4)

The reaction products after incubation of these bola SLs samples with rSBLE were again analyzed using HPLC, MALDI-TOF MS and, additionally, via LC–MS. For bola SLs sample 1 (INV-113), our data showed that, compared with the negative control (Fig. 7a), four lactonic SLs products (Fig. 7b, Table 4) were formed, in which di-acetylated lactonic SL (C_18:1_) is the most abundant product (RT 21.57 min). The peaks corresponding to the main bola SLs (Fig. 7a, Table 4) disappeared or decreased significantly after the reaction. Mass spectra of the major peaks are provided in Supplementary Figure S4. Curiously, since the product contains mainly di-acetylated lactones, our analyses reveal that in this INV-113 mixture, produced from an engineered *S. bombicola* strains, the main di-acetylated bola SL refers to acetylation at the sophorose group attached to the glycosyl bound sophorose, whereas the sophorose attached to the fatty acid carboxyl is non-acetylated. The product released upon the lactonization reaction is thus sophorose and not acetyl-sophorose. Further analysis of fermentation products is required to confirm this apparent asymmetric location of the acetyl groups. The release of sophorose is confirmed by HPLC analysis of the reaction mixture after the conversion (Supplementary Fig. S5).

**Table 4:** Overview of congeners present in a sample of bola SLs with high acetylation degree that displayed the largest change in intensity after incubation with rSBLE as shown in Fig. 7. Molecular mass as determined by LC–MS (Ac: acetyl)

Retention (RT, min)	Molecular mass	Type
Significantly decreased bola SLs		
9.048	988.5	Mono-Ac bola SL, C_18:1_
11.026	1030.4	Di-Ac bola SL, C_18:1_
11.814	1072.4	Tri-Ac bola SL, C_18:1_
12.046	1032.4	Di-Ac bola SL, C_18:0_
12.108	1072.4	Tri-Ac bola SL, C_18:1_
13.850	1114.4	Tetra-Ac bola SL, C_18:1_
Produced lactonic SLs		
19.703	648.2	Mono-Ac lactonic SL, C_18:0_
21.565	688.3	Di-Ac lactonic SL, C_18:1_
21.837	688.3	Di-Ac lactonic SL, C_18:1_
24.065	690.3	Di-Ac lactonic SL, C_18:0_

**Fig. 8:**
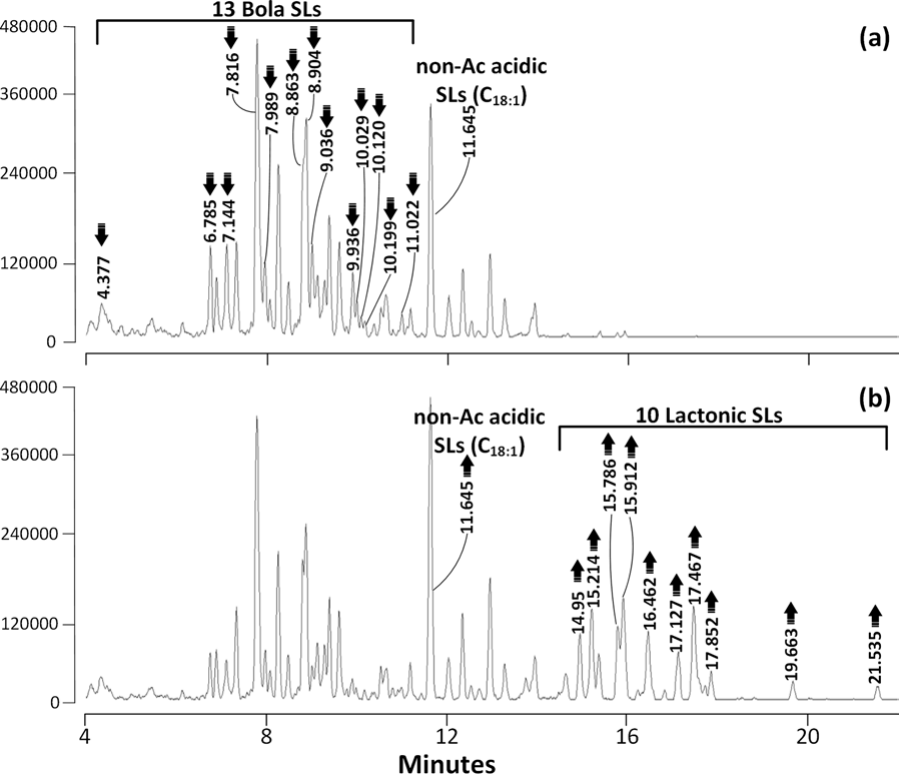
LC–MS TIC chromatograms from an activity assay of rSBLE on a sample mainly containing non-acetylated bola SLs (code: INV-22). The top chromatogram (**a**) is the mixture to which no enzyme is added. Peaks indicated with an arrow are mainly non- and mono-acetylated bola SLs (see Table 5) that decreased in intensity after incubation with the enzyme. The lower chromatogram (**b**) is obtained from the same SL mixture, but is obtained after incubation with rSBLE. Indicated peaks correspond mainly to non- and mono-acetylated lactonic SLs (Table 5)

This was also the case for the sample with mainly non-acetylated bola SLs (INV-22) (Fig. 8, Table 5). The results of the activity assays thus further confirm that SBLE converts acetylated bola SLs (acetylation degree mono-, di-, tri- and tetra- to form the corresponding lactonic SLs). All peaks that decreased in intensity after reaction with rSBLE corresponded to bola SLs, whereas peaks corresponding to acidic SLs remained unchanged after reaction.

**Table 5:** Overview of congeners present in a sample of bola SLs with lower acetylation degree that displayed the largest change in intensity after incubation with rSBLE as shown in Fig. 8. Molecular mass as determined by LC–MS (Ac: acetyl)

Retention (RT, min)	Molecular mass	Type
Significantly decreased bola SLs		
4.377	962.3	Mono-Ac bola SL, C_16:0_
6.785	920.3	Non-Ac bola SL, C_16:0_
7.144	920.3	Non-Ac bola SL, C_16:0_
7.816	946.3	Non-Ac bola SL, C_18:1_
7.989	960.4	Mono-Ac bola SL, C_16:1_
8.863	948.4	Non-Ac bola SL, C_18:0_
8.904	988.5	Mono-Ac bola SL, C_18:1_
9.036	988.5	Mono-Ac bola SL, C_18:1_
9.936	990.4	Mono-Ac bola SL, C_18:0_
10.029	990.4	Mono-Ac bola SL, C_18:0_
10.120	1030.4	Di-Ac bola SL, C_18:1_
10.199	1028.3	Di-Ac bola SL, C_18:2_
11.022	1030.4	Di-Ac bola SL, C_18:1_
Produced lactonic SLs		
14.95	604.2	Non-Ac lactonic SL, C_18:1_
15.214	604.3	Non-Ac lactonic SL, C_18:1_
15.786	686.2	Di-Ac lactonic SL, C_18:2_
15.912	604.3	Non-Ac lactonic SL, C_18:1_
16.462	620.3	Mono-Ac lactonic SL, C_16:0_
17.127	606.4	Non-Ac lactonic SL, C_18:0_
17.467	646.3	Mono-Ac lactonic SL, C_18:1_
17.852	646.2	Mono-Ac lactonic SL, C_18:1_
19.663	648.4	Mono-Ac lactonic SL, C_18:0_
21.535	688.3	Di-Ac lactonic SL, C_18:1_
Significantly increased acidic SLs		
11.645	622.3	Non-Ac acidic SL, C_18:1_

Performing the same experiment on a mixture of bola SLs containing mainly non- and mono-acetylated congeners (bola SLs sample 2, INV-22) also resulted in the formation of lactonic SLs (Table 5). However, in this case the peaks corresponding to lactonic products are lower and the bola SLs did not disappear completely. The enzyme seems thus less efficient, maybe due to weaker affinity towards the more polar substrates. In this case, we also observed an increase of the peak corresponding to non-acetylated acidic SLs indicating hydrolysis of the glycosyl ester bond. This may indicate some hydrolytic activity of SBLE resulting in a premature release of the acyl intermediate, although we cannot conclude spontaneous chemical hydrolysis under the conditions used.

## Discussion

SBLE, a lipase-like enzyme of the CALA family [13], was characterized in previous studies [15, 27]. The activity of this enzyme was at that time assessed using a sophorolipid mixture isolated from a *S. bombicola* ∆*sble* strain, presumed to only contain acidic SLs with acetylation degree from non- to di-. It was thus assumed that SBLE acts as a reverse lactonase catalyzing the ring closure of open acidic SLs to form lactonic SLs. However, in recent studies, it was found that this ∆*sble* strain actually produces a mixture of bola SLs and acidic SLs [31]. The activity of heterogeneously produced rSBLE was therefore re-investigated by performing the activity assays on different, more homogeneous SL samples.

Most recently, SL products with high purity have indeed become obtainable as a result of the improvements in SL preparation in terms of production process [33–36] and downstream processing (DSP) [6, 37, 38]. In this work, aiming to the re-characterization of SBLE, three samples consisting of purified acidic SLs (di-acetylated, C_18:1_, *ω*-1/*ω* and non-acetylated, C_18:1_, *ω*-1) were used in an in vitro enzymatic activity assay to assess its ability towards the lactonization of these three pure substrates. However, no lactonic SL could be detected in any of the reaction products. For non-acetylated SL, this was expected in view of what was observed in Ciesielska, Roelants [15], i.e., that SBLE only would accept acetylated precursors in vitro. However, we could not confirm the activity on di-acetylated precursors either. In fact, we even used higher E:S ratios and prolonged incubation time compared to previous studies, but that did not affect the results. We also checked if the reaction mixture contains polymerization products of di-acetylated substrates based on previous reports that SLs polymerization occurs naturally [28]. However, a MALDI-TOF/MS analysis did not provide any evidence for this.

**Fig. 9:**
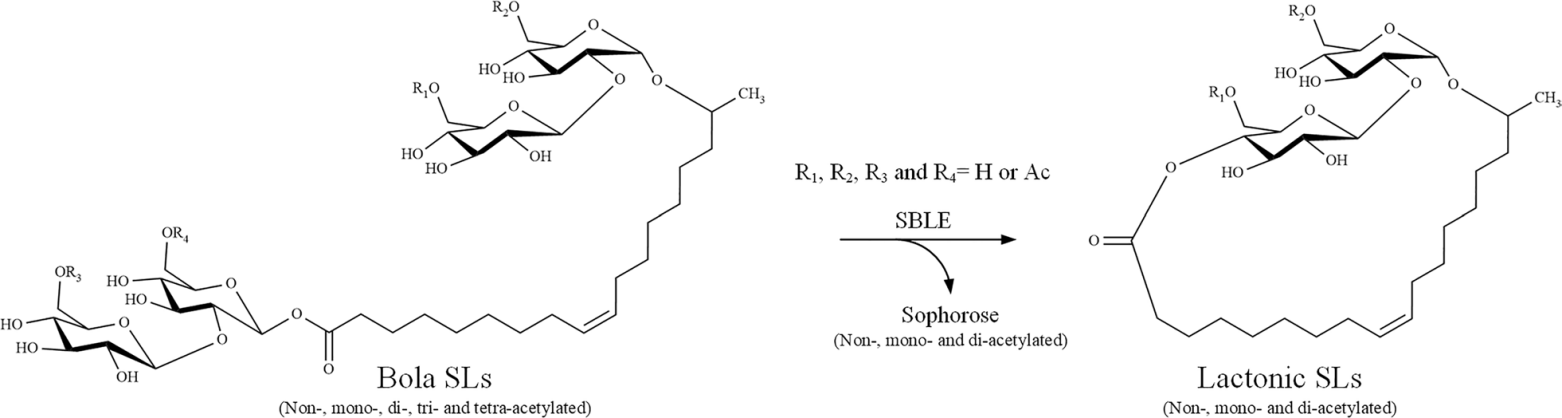
Revised reaction catalyzed by SBLE

Overall, in contrast to what we previously believed, SBLE was thus incapable of catalyzing the lactonization of acidic SLs in vitro. Therefore, an enzymatic activity assay was repeated using the original substrate mixture, derived from the Δ*sble* strain. Our analysis clearly revealed that apart from open acidic SLs, this product also contains bola SLs, a product that was hardly known during our previous work on the SBLE enzyme. Bola SLs, are present only in a nearly undetectable amount in the natural SL mixture from WT *S. bombicola* [28]. However, recent studies described the production of non-acetylated bolaform SLs using a *S. bombicola* strain deficient in both acetyltransferase (Δ*at*) and SBLE (Δ*sble*) genes [29, 30]. By comparing the HPLC chromatogram of the SL mixture obtained from the Δ*sble* strain with those of the purified acidic SLs, some additional components are indeed present. We found that exactly these components disappeared or declined significantly after incubation with rSBLE resulting in the formation of di-acetylated lactonic SLs. The compounds that declined upon incubation with rSBLE were characterized by MALDI-TOF MS and show m/z values corresponding to bola SLs, i.e., mono-acetylated (C_18:1_) and two di-acetylated (C_18:2_ and C_18:1_) bola SL. Consequently, on the basis of the novel finding that the formation of lactonic SLs was catalyzed by SBLE in vitro only in the presence of bola SLs, but not with acidic SLs. As such, it was found that bola SLs are the actual substrates of SBLE, and that SBLE would catalyze a transesterification reaction rather than an intramolecular esterification (lactonization) reaction as proposed previously [13, 15] (Fig. 9).

Aiming to confirm our hypothesis about this revised function of SBLE, two products enriched for bola SLs (bola SLs sample 1: INV-113 and 2: INV-22) were used as substrates in the subsequent enzymatic activity assay of rSBLE. The new role of SBLE was evaluated first using bola SLs sample 1. Six compounds displayed a significant reduction or disappearance in this substrate after incubation with the enzyme and were identified as acetylated bola SLs (mono-acetylated with C_18:1_, di-acetylated with C_18:1_ and C_18:0_, two tri-acetylated with C_18:1_ and tetra-acetylated with C_18:1_) resulting in the synthesis of four types of lactonic congeners (mono-acetylated with C_18:0_, three di-acetylated, one with C_18:0_ and two with C_18:1_). Curiously, some other bola SLs were identified in this substrate that did not seem to be converted by the enzyme, such as a tri-acetylated bola (C_18:1_) which should possess a different structure than the two significantly changed tri-acetylated bola (C_18:1_) mentioned above since it has a different elution time [39]. This suggests that SBLE has substrate selectivity towards specific isomers of bola SLs, a phenomenon described for other lipases [40–43].

The second substrate, we exploited to verify our new insights in the actual catalytic activity of SBLE is bola SLs sample 2 (INV-22) which is enriched for mainly non- and mono-acetylated congeners. Again, we see conversion into lactonic SL, but in general the yield is somewhat lower indicating a lower transesterification efficiency of SBLE towards non-acetylated bola SLs compared to the acetylated precursors in vitro. This should be confirmed by a kinetic analysis. The finding that SBLE is able to produce non-acetylated lactonic SLs in vitro is actually a new finding for SBLE, referring to a previous observation in the literature that this enzyme was not able to form non-acetylated lactonic SL [15].

We do now believe that the proteins encoded by the sophorolipids biosynthetic gene cluster do not only produce acidic SLs, but also bola SLs. The biosynthesis of lactonic SLs by *S. bombicola* as it has been always described, i.e., an internal esterification reaction of acidic SLs giving rise to lactonic SLs is thus revisited here. In vivo experiments unveiling the actual intermediate products using SBLE mutants are proposed in an accompanying paper [31], confirming that revision of the sophorolipid biosynthetic pathway is required.

The fact that the SLs pathway involves bola SLs as an important intermediate has been overlooked, is due to fast turnover towards either acidic SLs (via hydrolysis) or towards lactonic SLs (through an intramolecular transesterification reaction) as described here. This new insight provides also an explanation of a long-standing question about the thermodynamics of lactonic SLs formation. The formation of lactonic SLs was previously not well understood in view of the seemingly unfavorable loss of entropy when considering a simple intramolecular esterification reaction. Our new finding unveils that this reaction is actually driven by the hydrolysis of a high-energy glycosyl ester bond. The capacity to perform transesterification reactions in high water environment is a property shared with CALA [44].

## Conclusion

This work provides completely new insights in the activity of the *Starmerella bombicola* SBLE enzyme and in the biosynthetic pathway of sophorolipids. The actual substrates of SBLE are not acidic SLs as previously thought, but bola SLs, indicating that final lactonic SL production involves a transesterification rather than a classical lactonization reaction. We provide evidence that SBLE acts best on acetylated bola SLs, but a more detailed kinetic study using further purified substrates should shed light on the actual substrate selectivity. This revised understanding of the biosynthetic pathway is imminent for a better control of SL production in *S. bombicola*.

### Supplementary Information

Supplementary Material 1.Figure S1. SDS-PAGE analysis of recombinant SBLE.

Supplementary Material 2. Figure S2. MALDI-TOF MS and MS/MS of HPLC purified sophorolipids.

Supplementary Material 3. Figure S3. LC–MS spectra of sophorolipid products obtained after incubation of bola SLs with rSBLE.

Supplementary Material 4. Figure S4. MS/MS spectra of bola SLs and formed lactonic SLs after enzymatic conversion with rSBLE.

Supplementary Material 5. Figure S5. HPLC analysis of the sophorose released during sophorolipid lactonization. Comparison of retention times with commercial sophorose.

## Data Availability

All data are available either in the main text or in the supplementary information.

## Abbreviations

ACN: Acetonitrile
BGC: Biosynthetic gene cluster
BMGY: Buffered Glycerol-complex Medium
BMMY: Buffered Methanol-complex Medium
CALA: Candida antarctica lipase A
CALB: Candida antartica lipase B
DSP: Downstream processing
HPLC: High performance liquid chromatography
IMAC: Immobilized metal affinity chromatography
LC–MS: Liquid chromatography coupled mass spectrometry
MALDI-TOF MS: Matrix-assisted laser desorption/ionization mass spectrometry
MW: Molecular weight
rSBLE: Recombinant *Starmerella bombicola* lactone esterase
RT: Retention time
SBLE: *Starmerella bombicola* Lactone esterase
SLs: Sophorolipids
TIC: Total ion current
THF: Tetrahydrofuran
UGTA1: UDP-glucosyl transferase 1
UV: Ultraviolet
