# Supplementary material for: Revision of the sophorolipid biosynthetic pathway in Starmerella bombicola based on new insights in the substrate profile of its lactone esterase
Source: Biotechnol Biofuels Bioprod. 2024 Jun 27;17:89. doi: 10.1186/s13068-024-02533-1 (PMC11210130; doi:10.1186/s13068-024-02533-1)
Supplement: Supplementary file 1 — Supplementary Material 1.Figure S1. SDS-PAGE analysis of recombinant SBLE. [file 13068_2024_2533_MOESM1_ESM.docx]

**Supplementary Information**

Fig S1 MALDI-TOF MS spectra of 3 significantly decreased bola SLs, di-acetylated acidic SL without reduction (see Fig. 3.6a) and produced lactonic SLs (see Fig. 3.6b) from an activity assay of rSBLE using the substrate of acidic SL mixture (ASL mix). (RT: retention; Ac: acetyl)


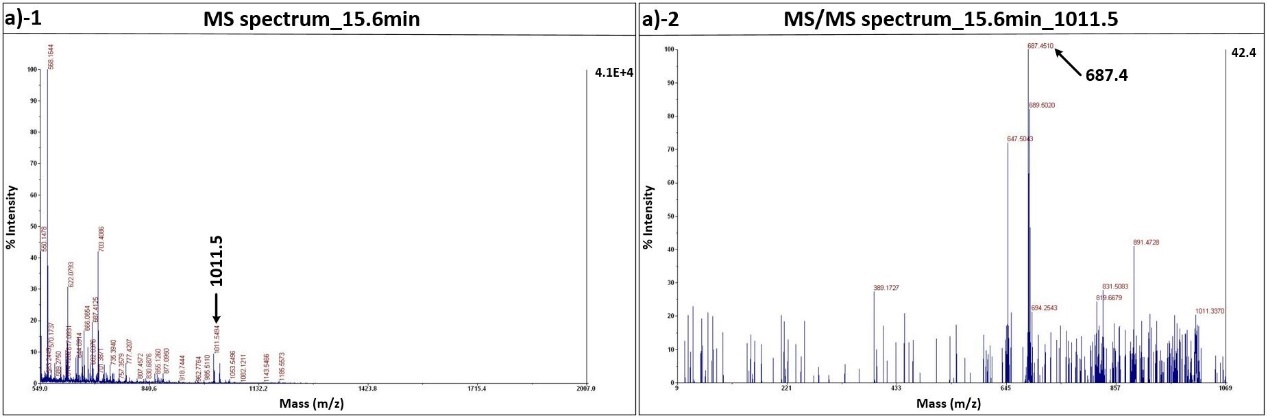

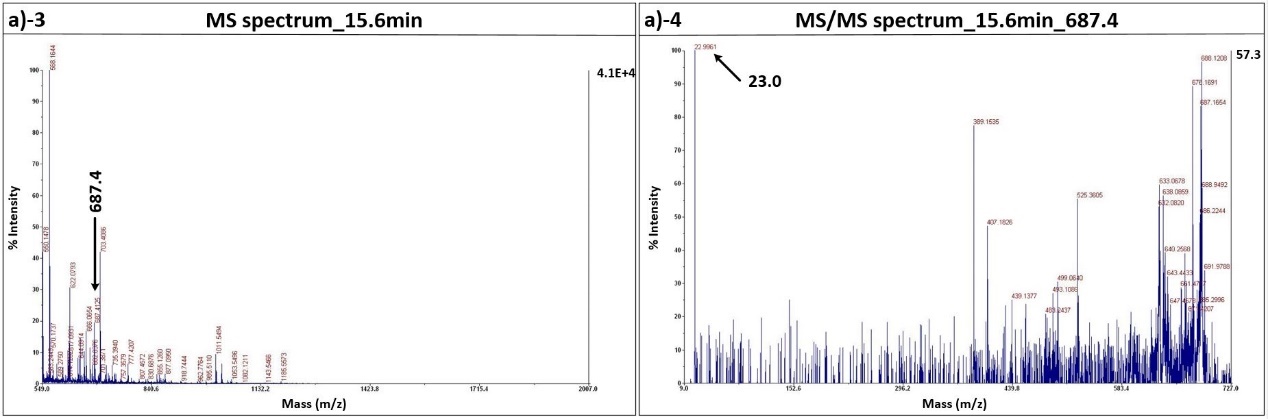


a) Maldi TOF MS and MS/MS spectra of HPLC fraction collected from the peak at retention time 15.6min. Panel 1: MS spectrum, indicating the presence of mono-Ac bola SL (C18:1) with molecular mass of 988 seen as a Na^+^-adduct with m/z 1011.5. Panel 2 : MS/MS spectrum of the 1011.5 precursor. The main fragment, with m/z 687.4 indicates the loss of a sophorosylgroup, presumably the sophorose that is acyl-esterified to the lipid carboxyl group. Panel 3 : displays the MS spectrum again, but marks a peak with the same m/Z value of 687.4 which is assumed to be the result of in-source fragmentation of bolaSL. Panel -4 shows the pseudo MS3 spectrum of this component, among which documenting the presence of Na^+^. This displays, amongst other fragments the loss of 162 Da, corresponding to a glucosyl unit.


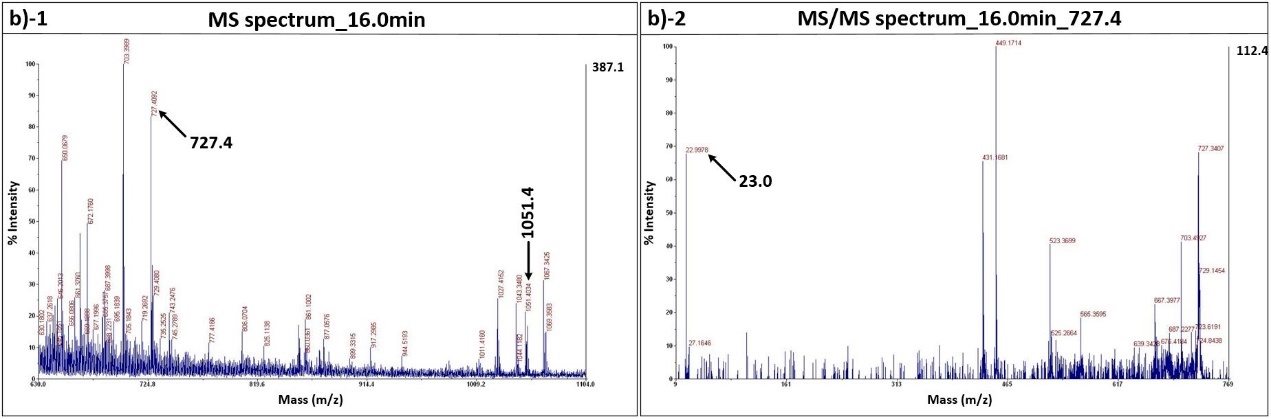


b. Maldi TOF MS and MS/MS spectra of HPLC fraction collected from the peak at retention time 16.0min. indicated di-Ac bola SL (C18:2) with molecular mass of 1028 seen as a Na+-adduct with m/z 1051.4. The peak intensity of this component was too low to obtain an informative MS/MS spectrum. However, similar as in the previous example, an in-source fragment corresponding to the loss of a sophorose molecule was observed at 727.5Da. b) Panel -2 shows the pseudo MS3 spectrum of this component, among which documenting the presence of Na^+^. This spectrum displays, amongst other fragments, the loss of 204 Da, corresponding to an N-acetylglucosyl unit.


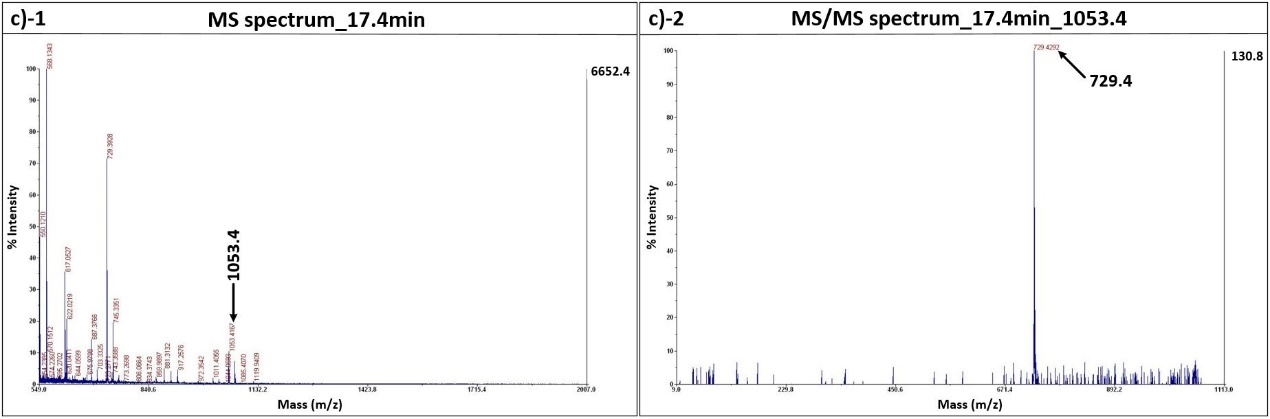

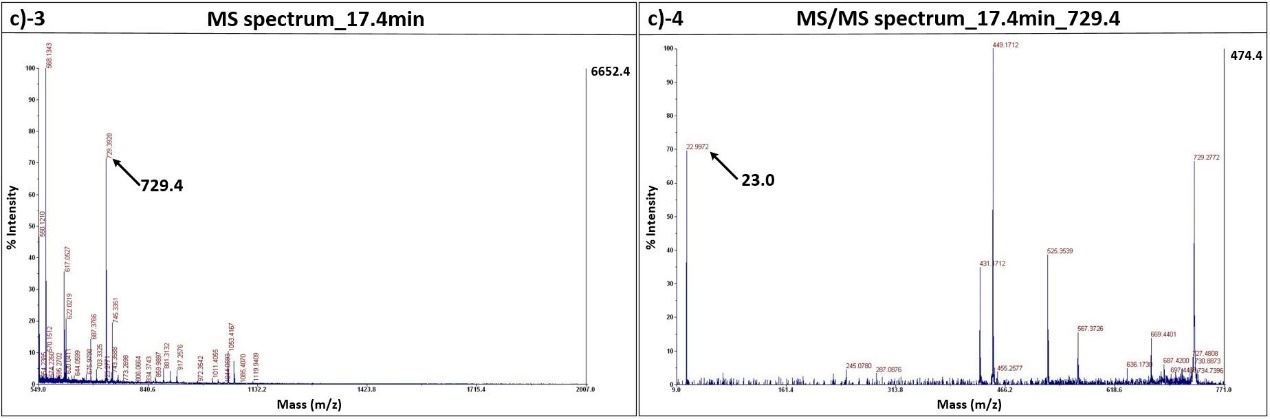


c) Panel 1. Maldi TOF MS and MS/MS spectra of HPLC fraction collected from the peak at retention time of 17.4min indicated di-Ac bola SL (C18:1) with molecular mass of 1030, displayed as the sodium adduct with m/z. 1053.4. Panel 2. MS/MS spectrum of the peak with m/Z 1053.4 resulting in a mean fragment at m/Z 729.4 corresponding to the loss of a sophorosyl fragment. Panel c. Similar as in a and b, this fragment was visible in the MS spectrum as an in-source fragment. Panel d. Pseudo MS3 spectrum of this fragment, showing amongst other fragments a loss of 204 Da corresponding to the loss of N-acetylglucose


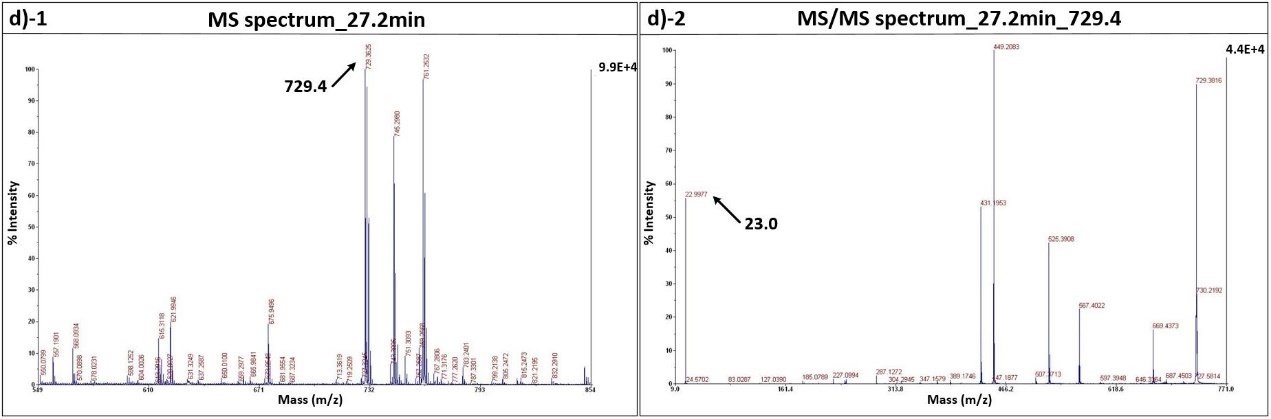


d) Panel 1. Maldi TOF MS and MS/MS spectra of HPLC fraction collected from the peak at retention time of 27.2 min, corresponding to di-Ac acidic SL (C18:1) with molecular mass of 706, visualized as the sodium adduct. Panel 2. MS/MS spectrum of this product.


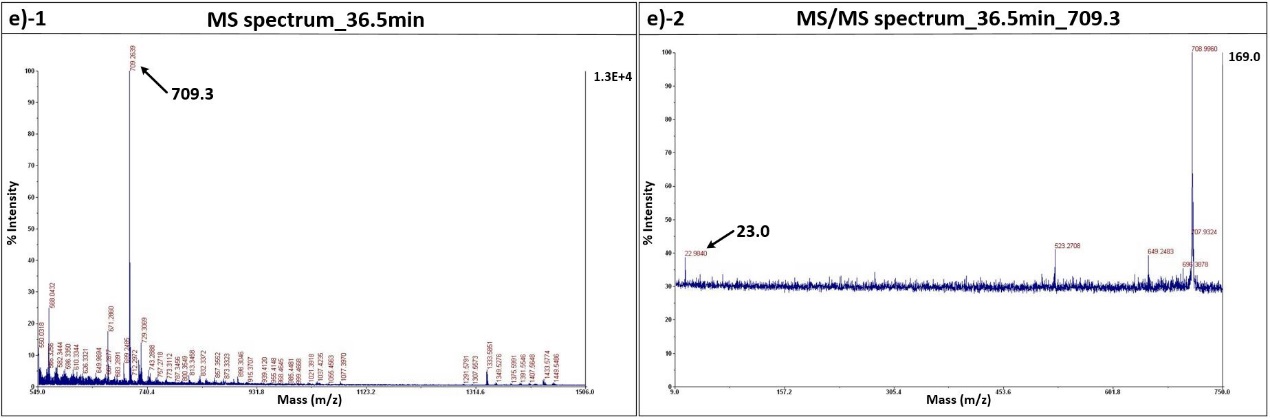


e) Panel 1. Maldi TOF MS and MS/MS spectra of HPLC fraction collected from the peak at retention time of 36.5min corresponding to di-Acetyl lactonic SL (C18:2) with molecular mass of 686, visualized as the Na+ adduct. Panel 2. MS/MS spectrum of this component.


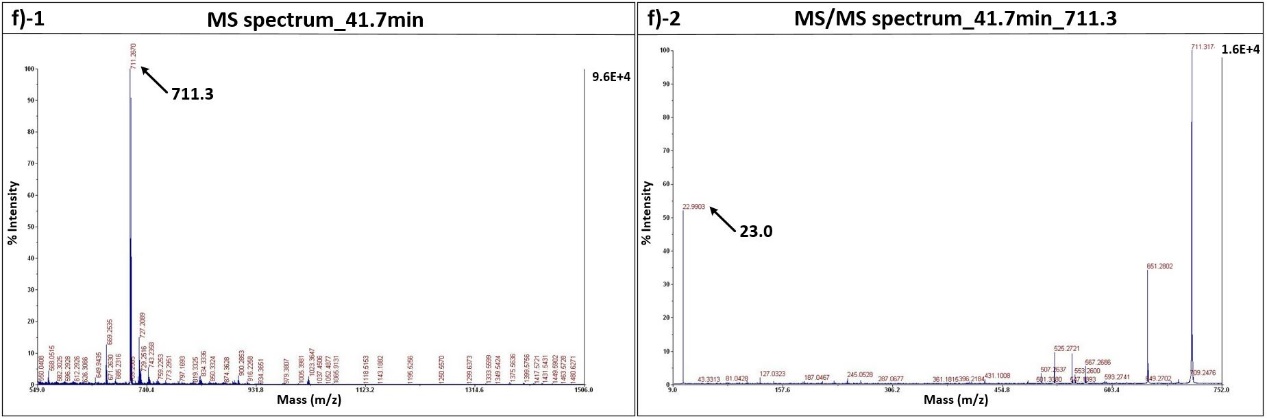


f) MaldiTOF MS and MS/MS spectra of HPLC fraction collected from the peak at retention time of 41.7min corresponding to di-acetyl lactonic SL (C18:1) with molecular mass of 688, visualized as the Na+ adduct. Panel 2. MS/MS spectrum of this component.
