# Supplementary material for: Revision of the sophorolipid biosynthetic pathway in Starmerella bombicola based on new insights in the substrate profile of its lactone esterase
Source: Biotechnol Biofuels Bioprod. 2024 Jun 27;17:89. doi: 10.1186/s13068-024-02533-1 (PMC11210130; doi:10.1186/s13068-024-02533-1)
Supplement: Supplementary file 2 — Supplementary Material 2. Figure S2. MALDI-TOF MS and MS/MS of HPLC purified sophorolipids. [file 13068_2024_2533_MOESM2_ESM.docx]

Fig S2 LC-MS spectra of significantly decreased bola SLs (see Fig 3.7a) and produced lactonic SLs (see Fig 3.7b) from an activity assay of rSBLE using the substrate of bola SL sample 1 (code: INV-113).


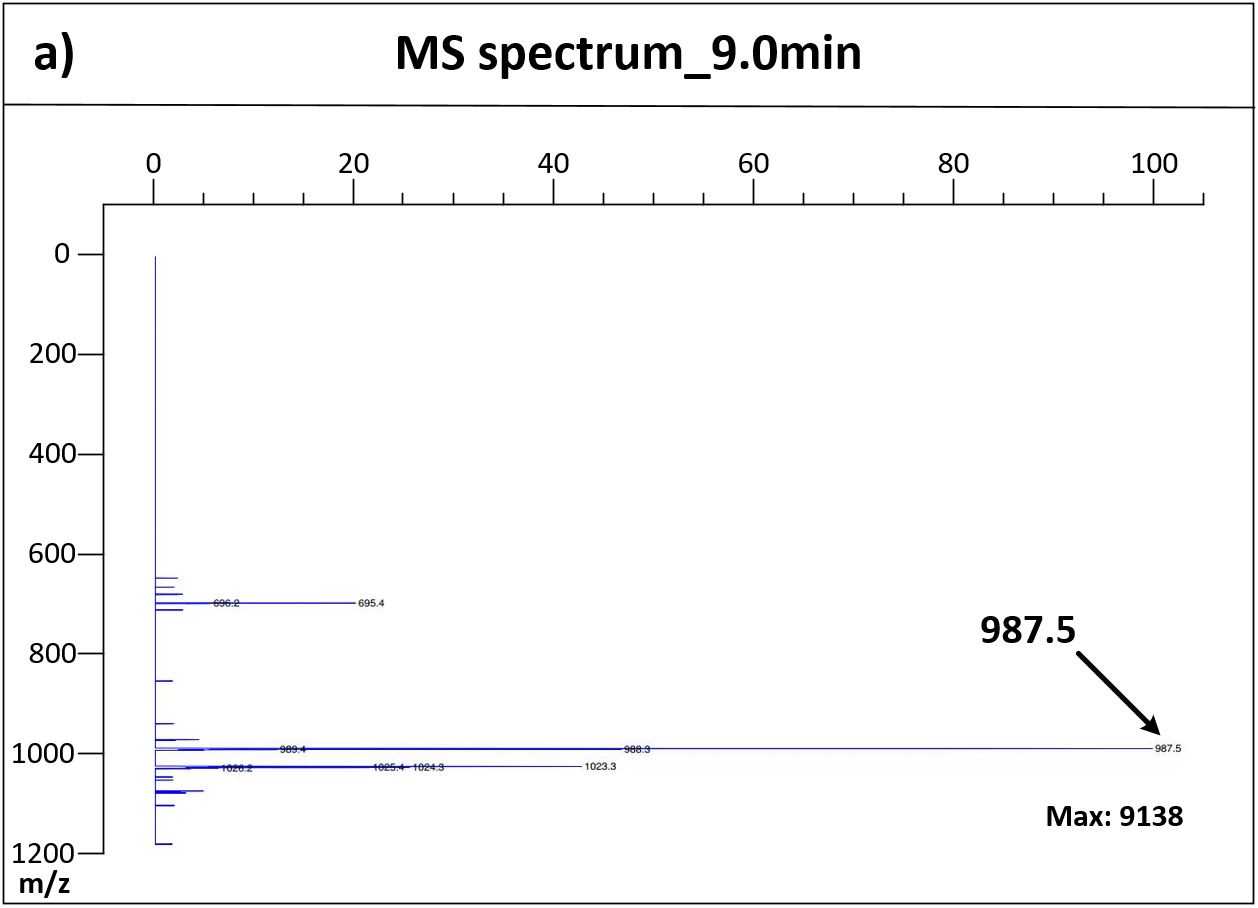


a). Mass spectrum of fractions of peak at RT of 9.0min indicated mono-Ac bola SL (C18:1) with molecular mass of 987.5.


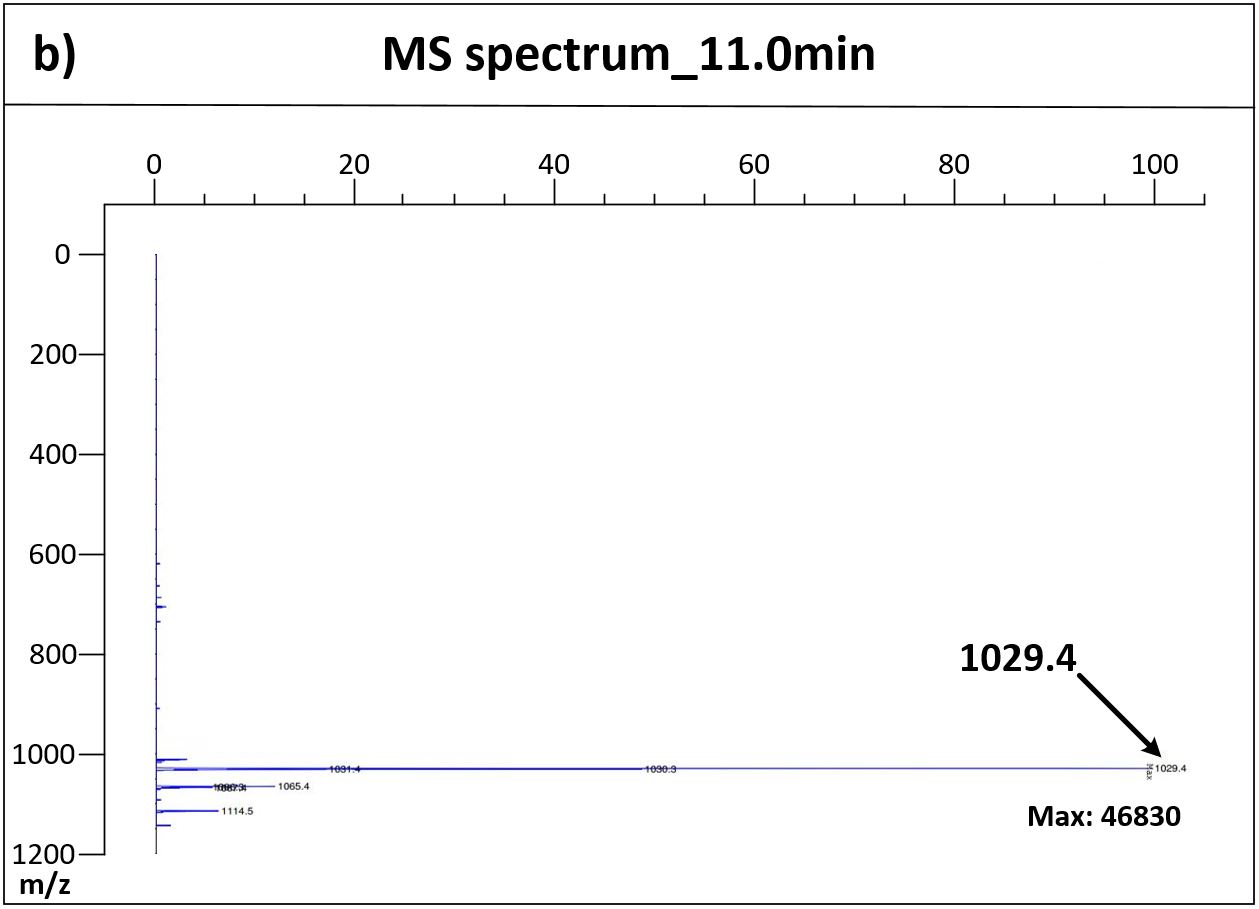


b). Mass spectrum of fractions of peak at RT of 11.0min indicated di-Ac bola SL (C18:1) with molecular mass of 1029.4.


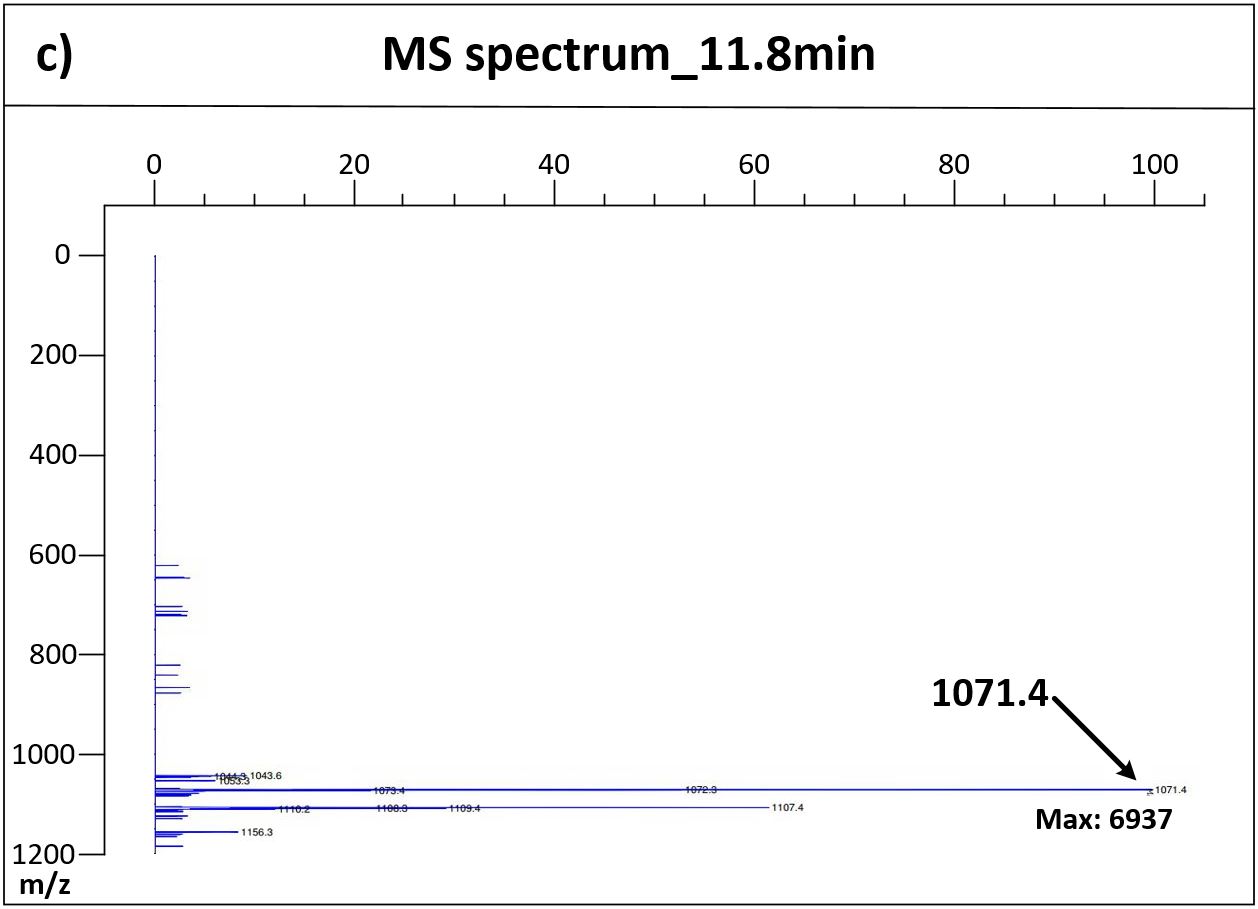


c). Mass spectrum of fractions of peak at RT of 11.8min indicated tri-Ac bola SL (C18:1) with molecular mass of 1071.4


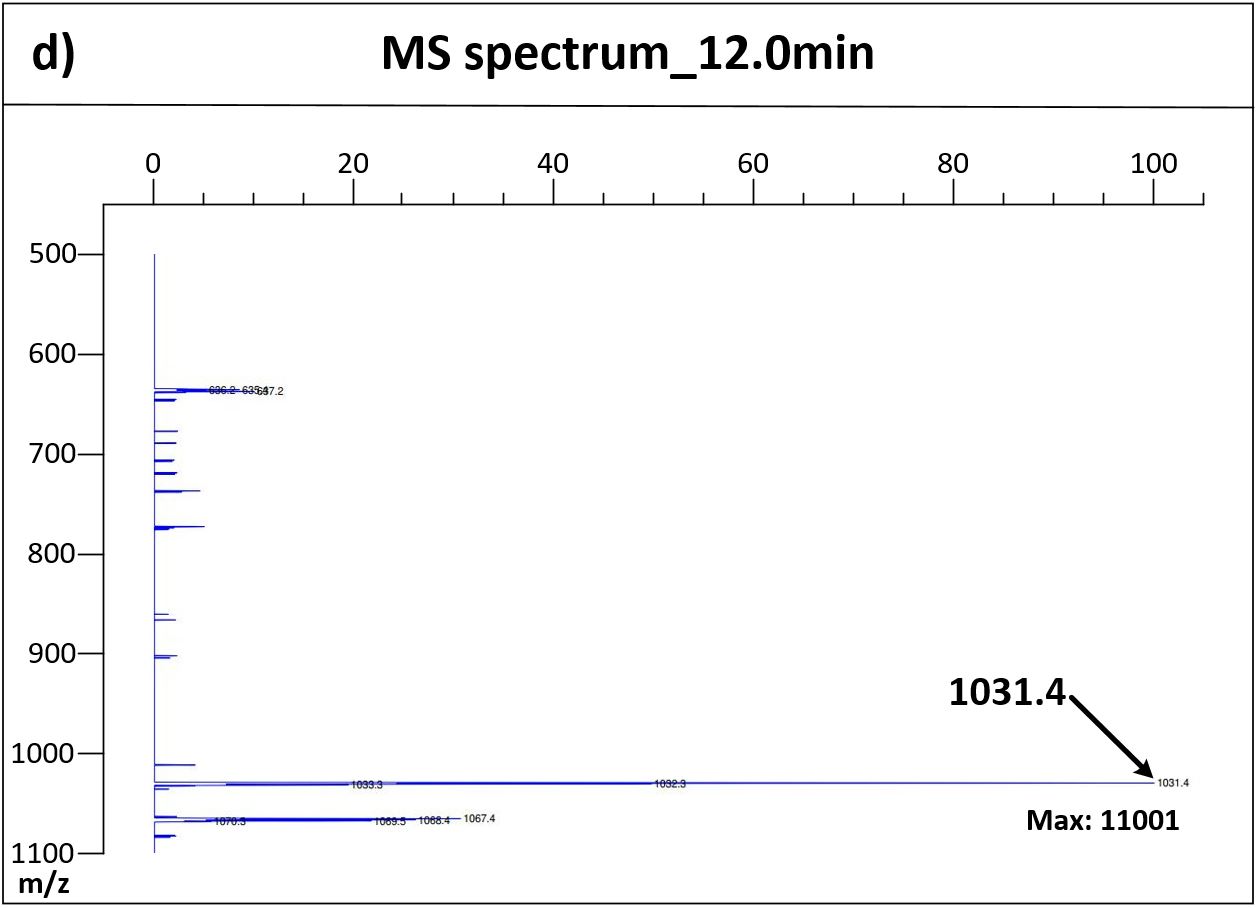


d). Mass spectrum of fractions of peak at RT of 12.0min indicated di-Ac bola SL (C18:0) with molecular mass of 1032.


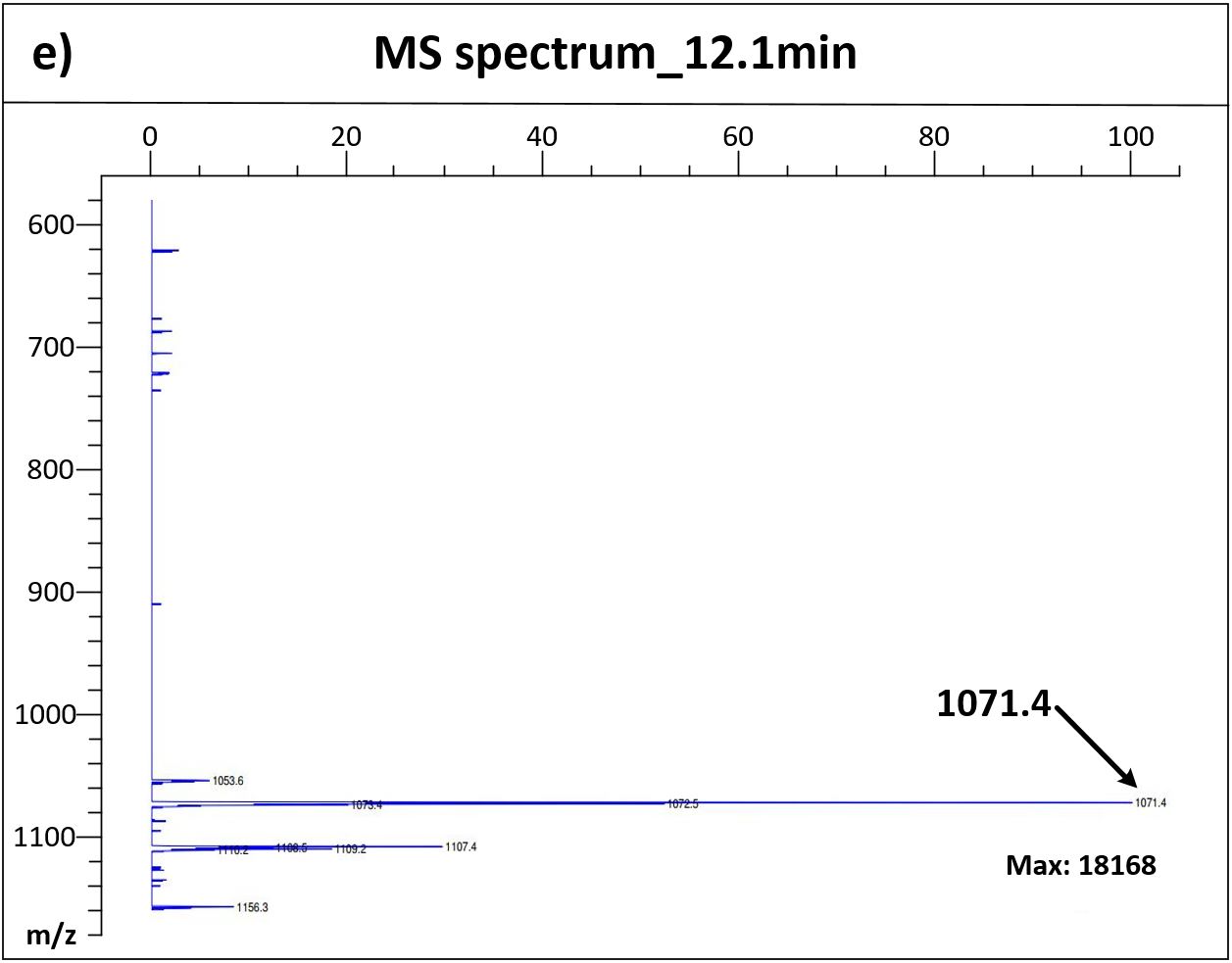


e). Mass spectrum of fractions of peak at RT of 12.1min indicated tri-Ac bola SL (C18:1) with molecular mass of 1072.


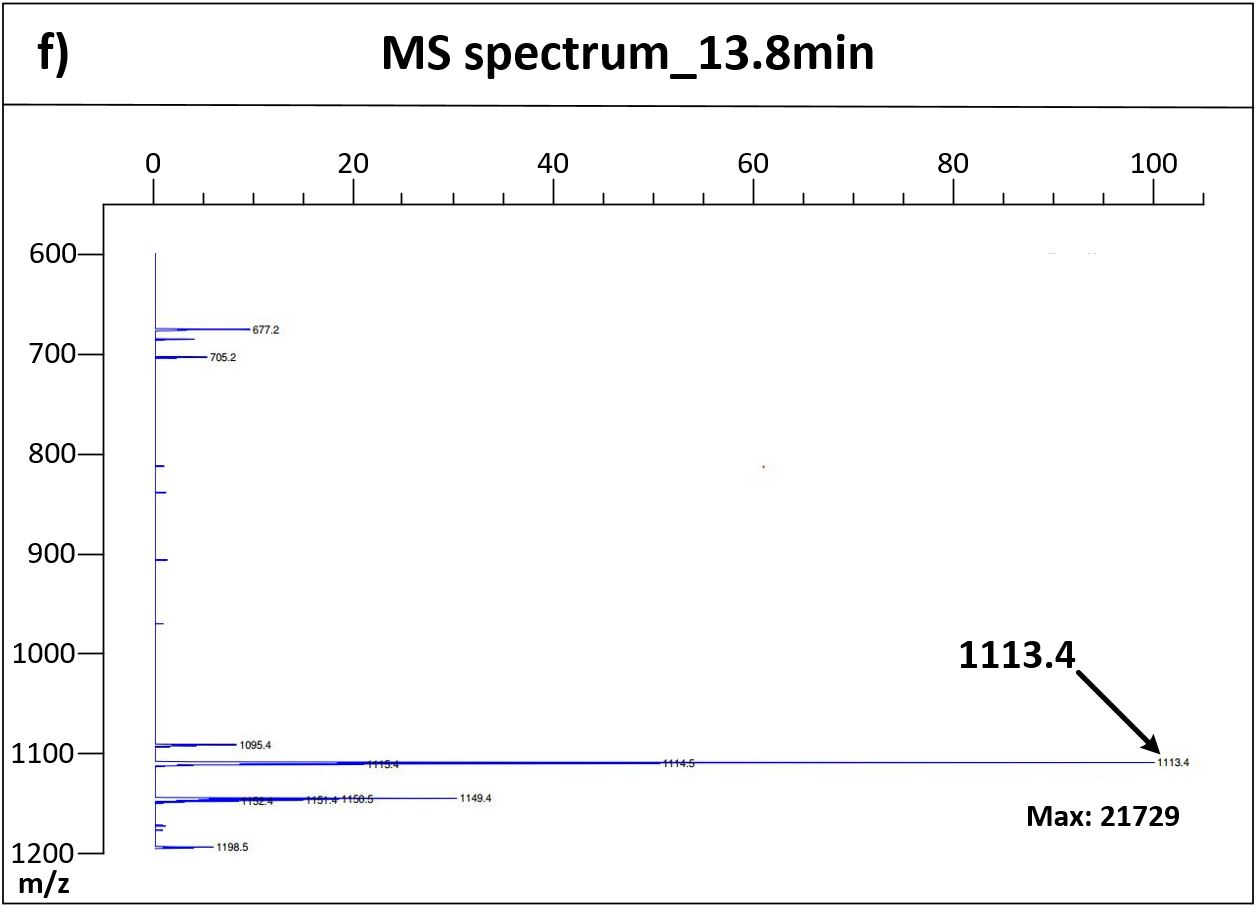


f). Mass spectrum of fractions of peak at RT of 13.8min indicated tetra-Ac bola SL (C18:1) with molecular mass of 1114.


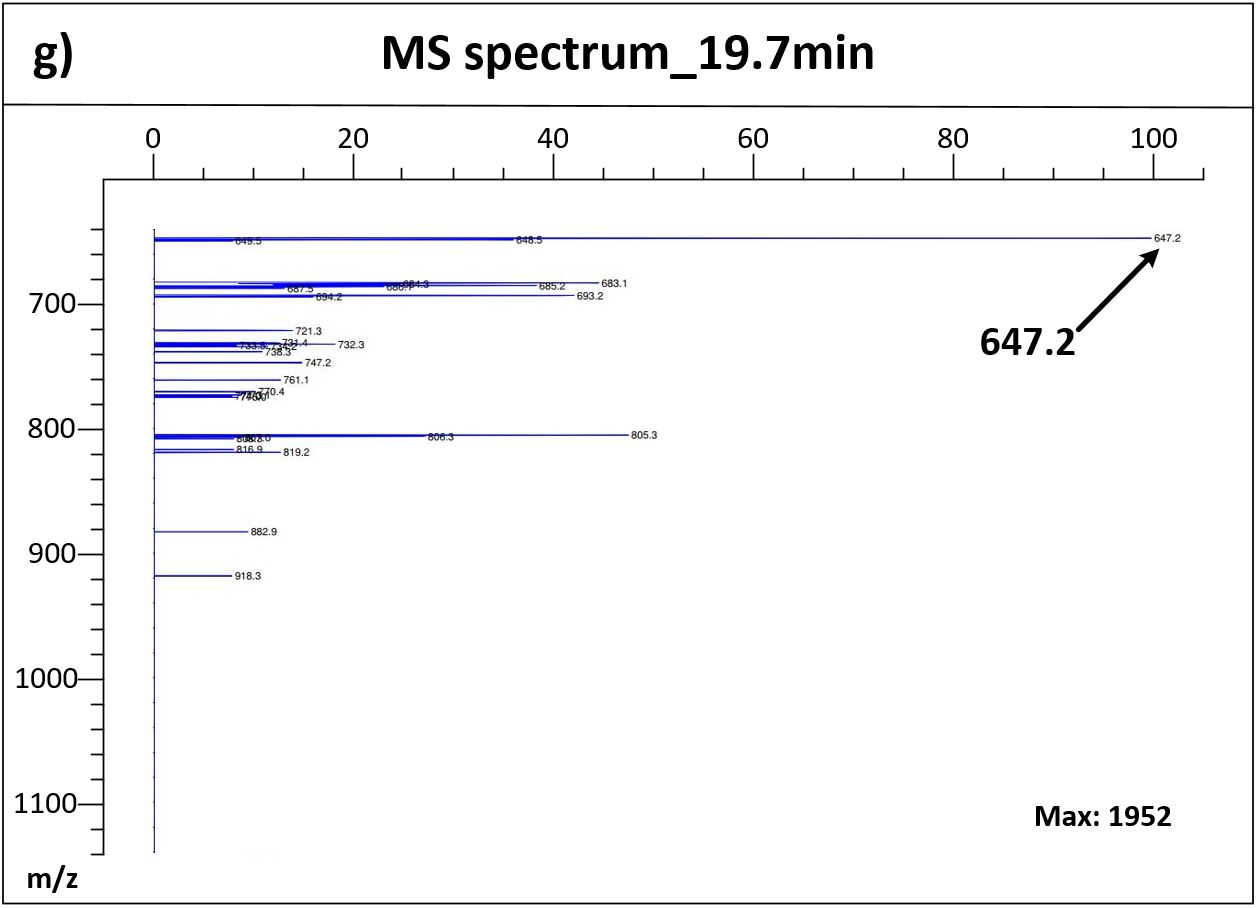


g). Mass spectrum of fractions of peak at RT of 19.7min indicated mono-Ac lactonic SL (C18:0) with molecular mass of 648.


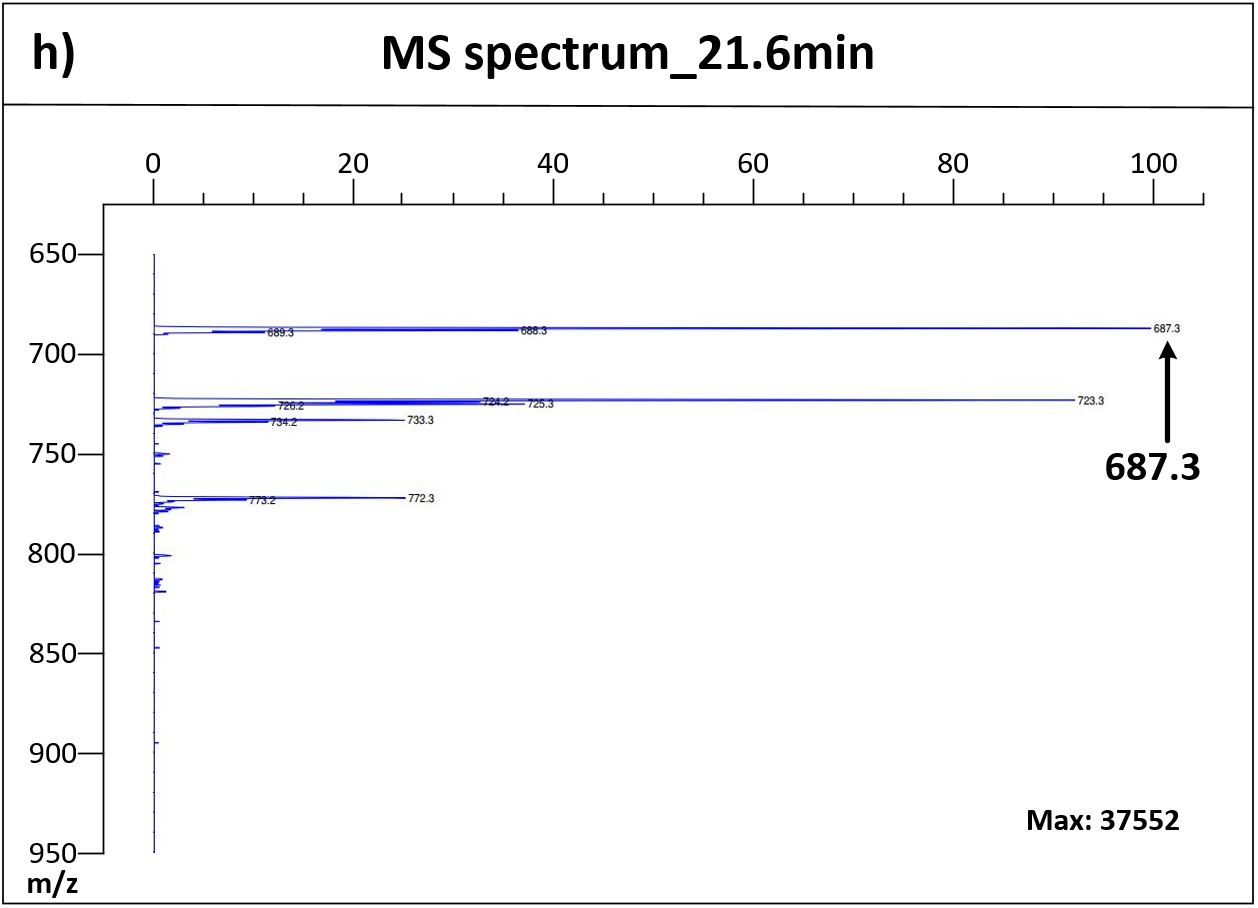


h). Mass spectrum of fractions of peak at RT of 21.6min indicated di-Ac lactonic SL (C18:1) with molecular mass of 688.


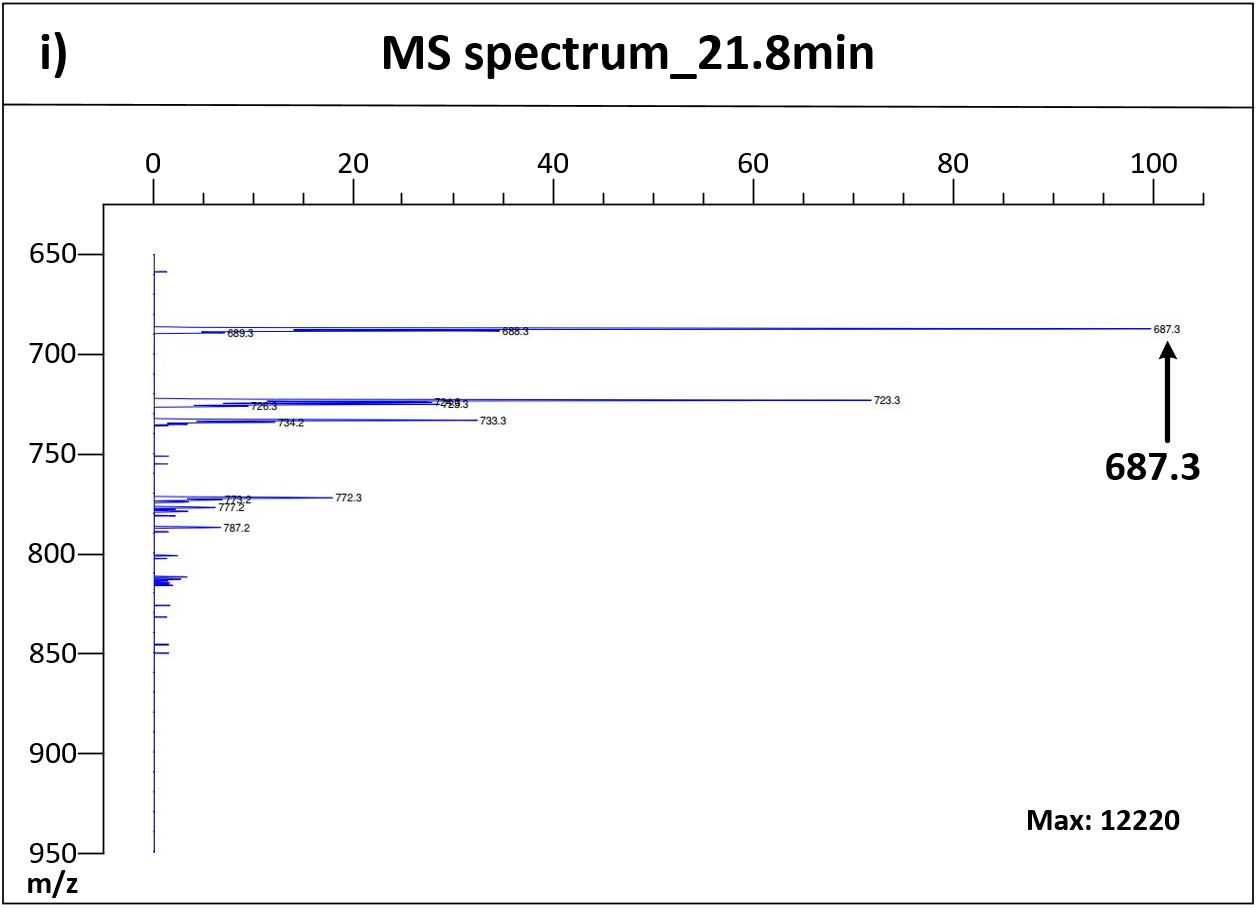


i). Mass spectrum of fractions of peak at RT of 21.8min indicated di-Ac lactonic SL (C18:1) with molecular mass of 688.


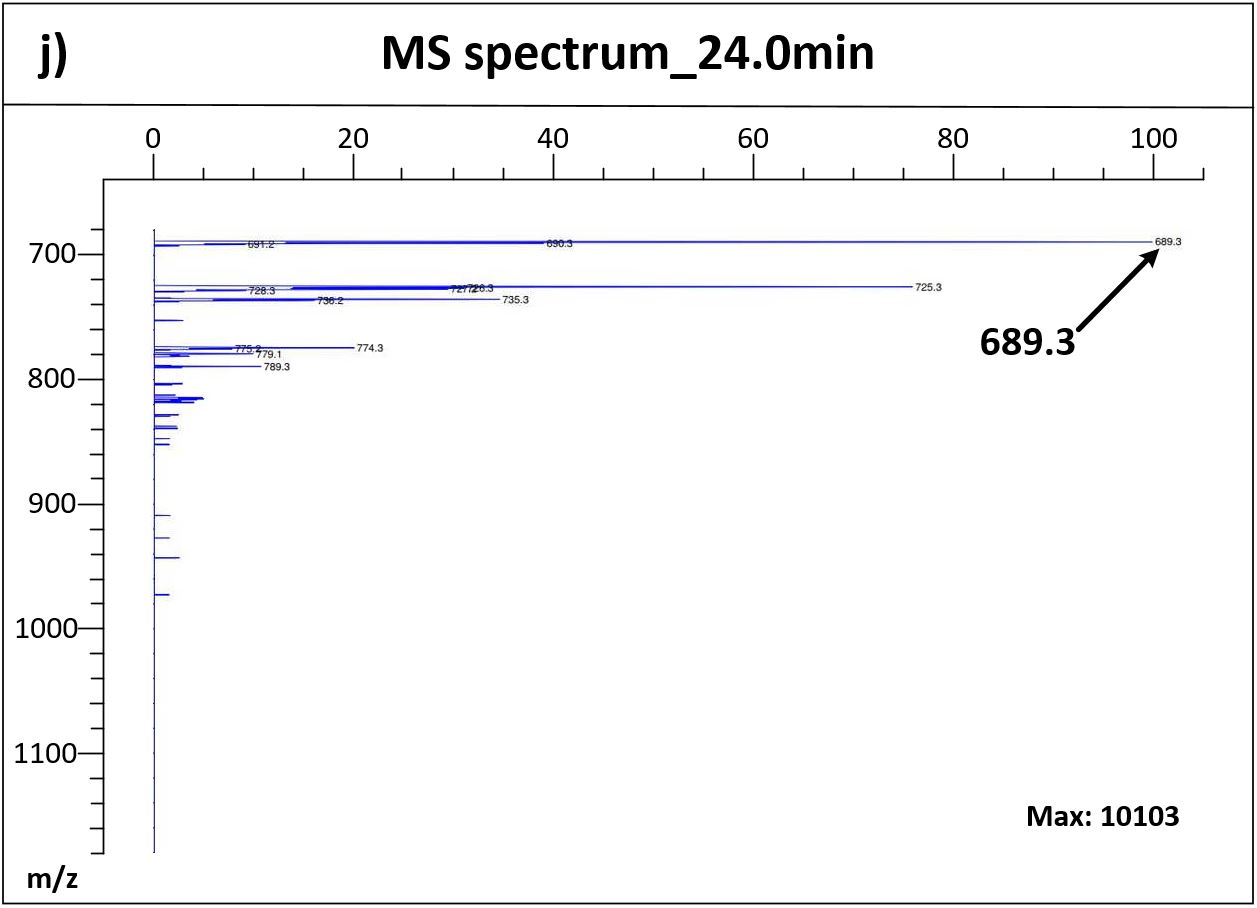


j). Mass spectrum of fractions of peak at RT of 24.0min indicated di-Ac lactonic SL (C18:0) with molecular mass of 690.

Fig S3.2 LC-MS spectra of significantly decreased bola SLs (see Fig 3.8a), produced lactonic SLs and obviously accumulated non-acetylated acidic SL (see Fig 3.8b) from an activity assay of rSBLE using the substrate of bola SL sample 2 (code: INV-22).


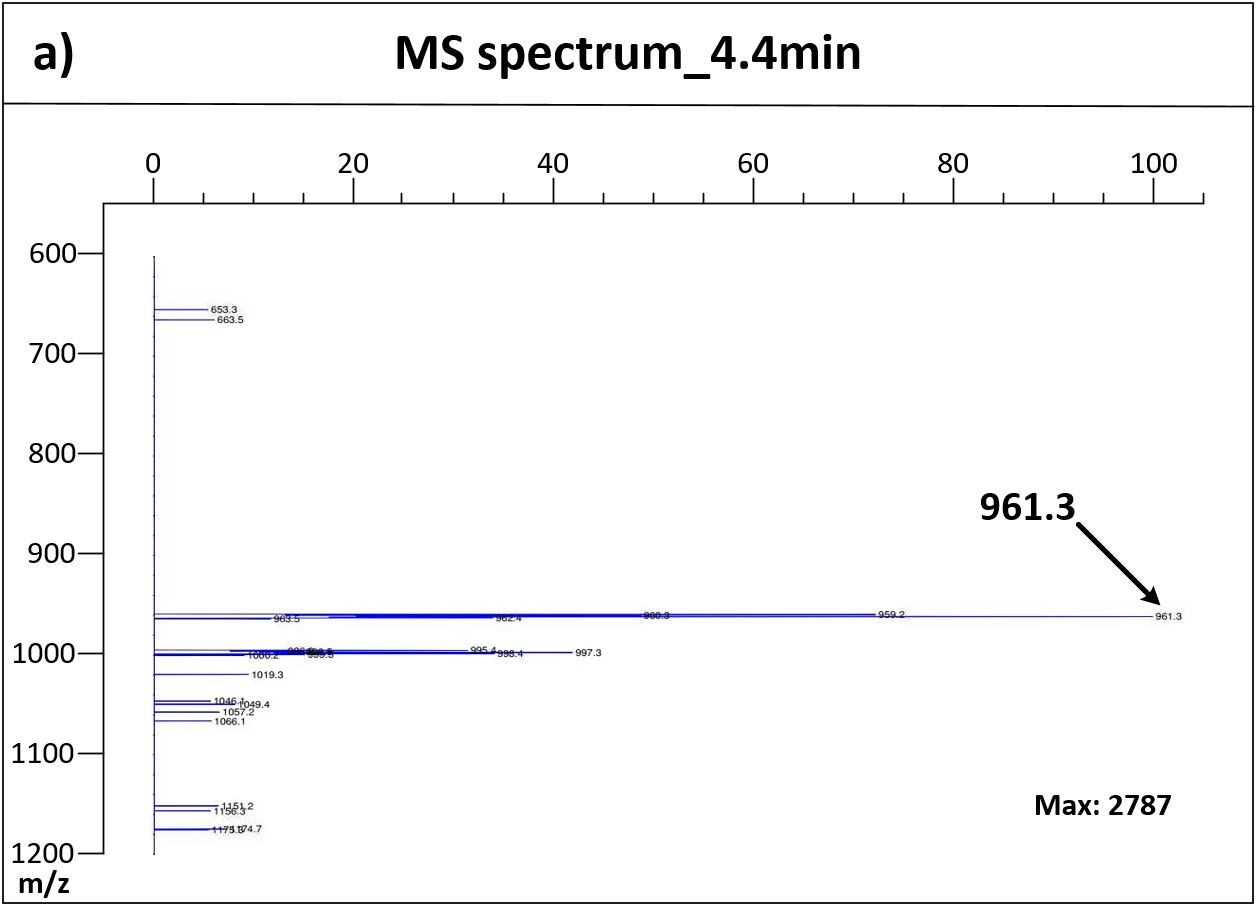


a). Mass spectrum of fractions of peak at RT of 4.4min indicated mono-Ac bola SL (C16:0) with molecular mass of 962.


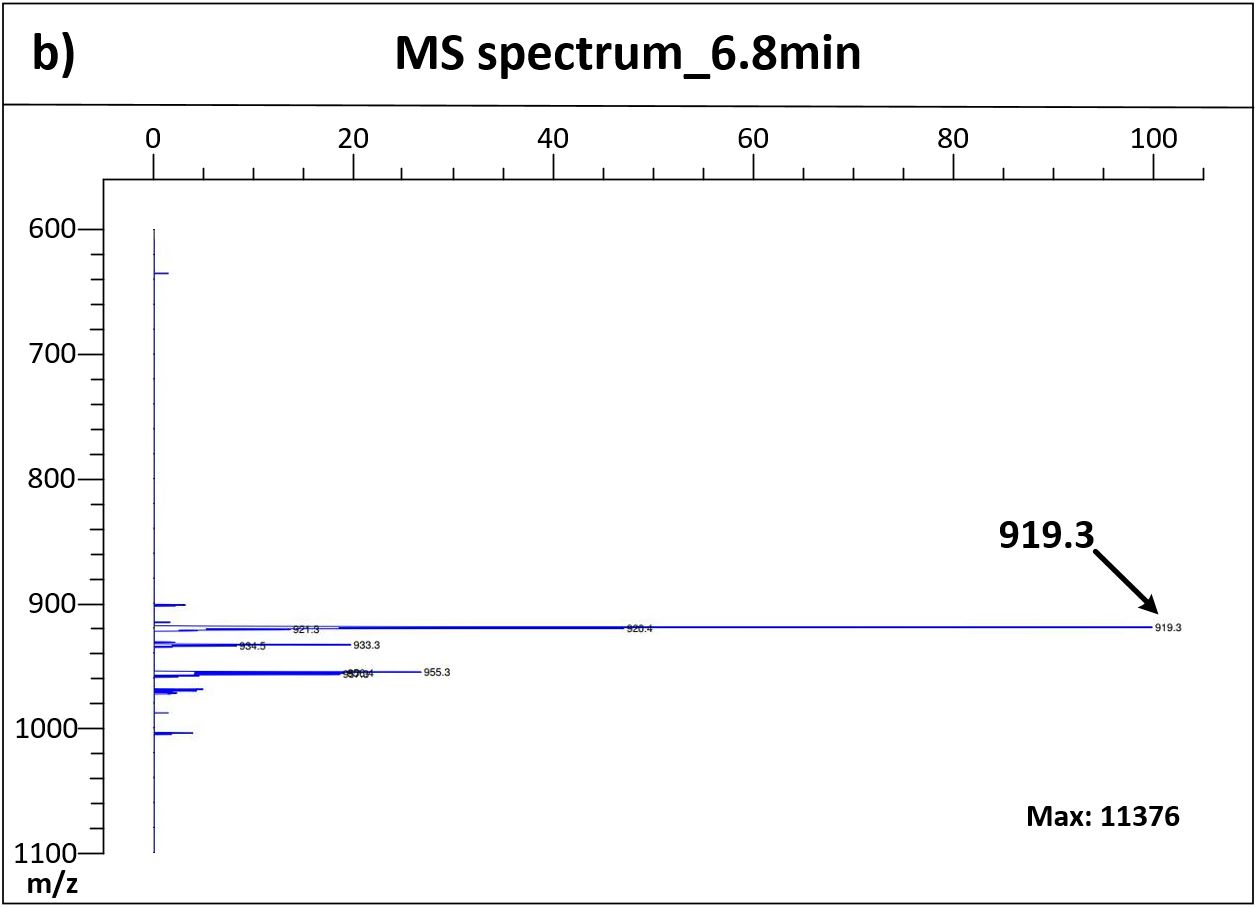


b). Mass spectrum of fractions of peak at RT of 6.8min indicated non-Ac bola SL (C16:0) with molecular mass of 920.


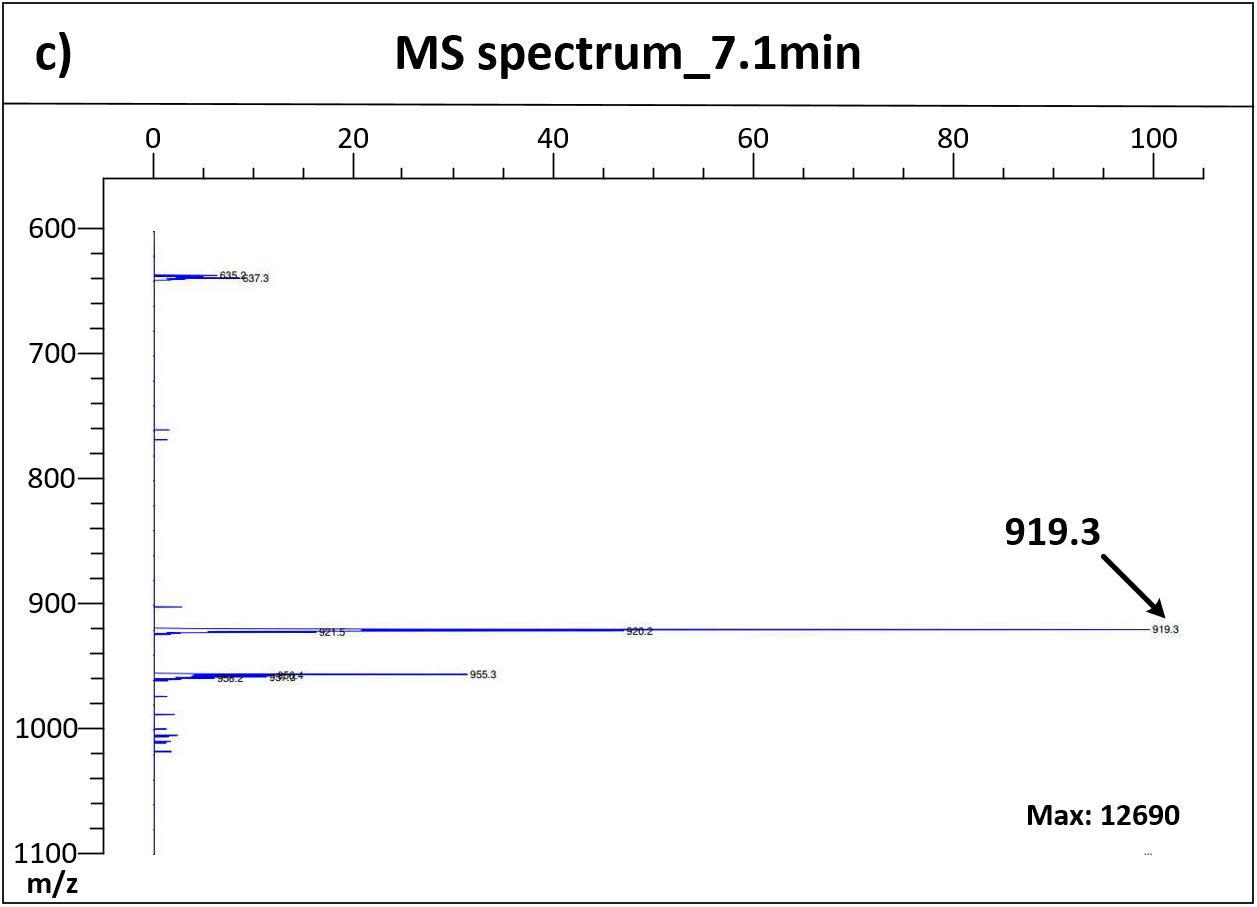


c). Mass spectrum of fractions of peak at RT of 7.1min indicated non-Ac bola SL (C16:0) with molecular mass of 920.


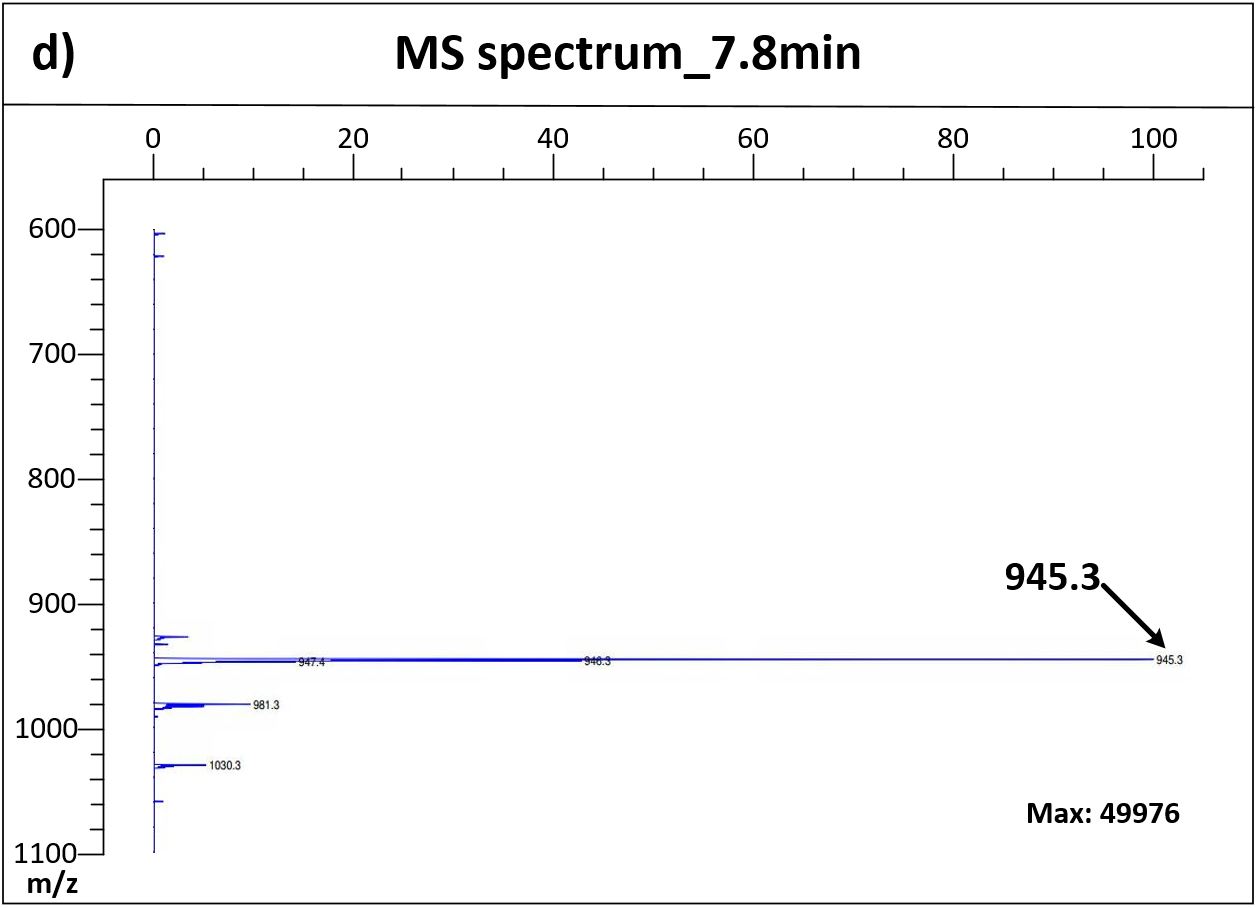


d). Mass spectrum of fractions of peak at RT of 7.8min indicated non-Ac bola SL (C18:1) with molecular mass of 946.


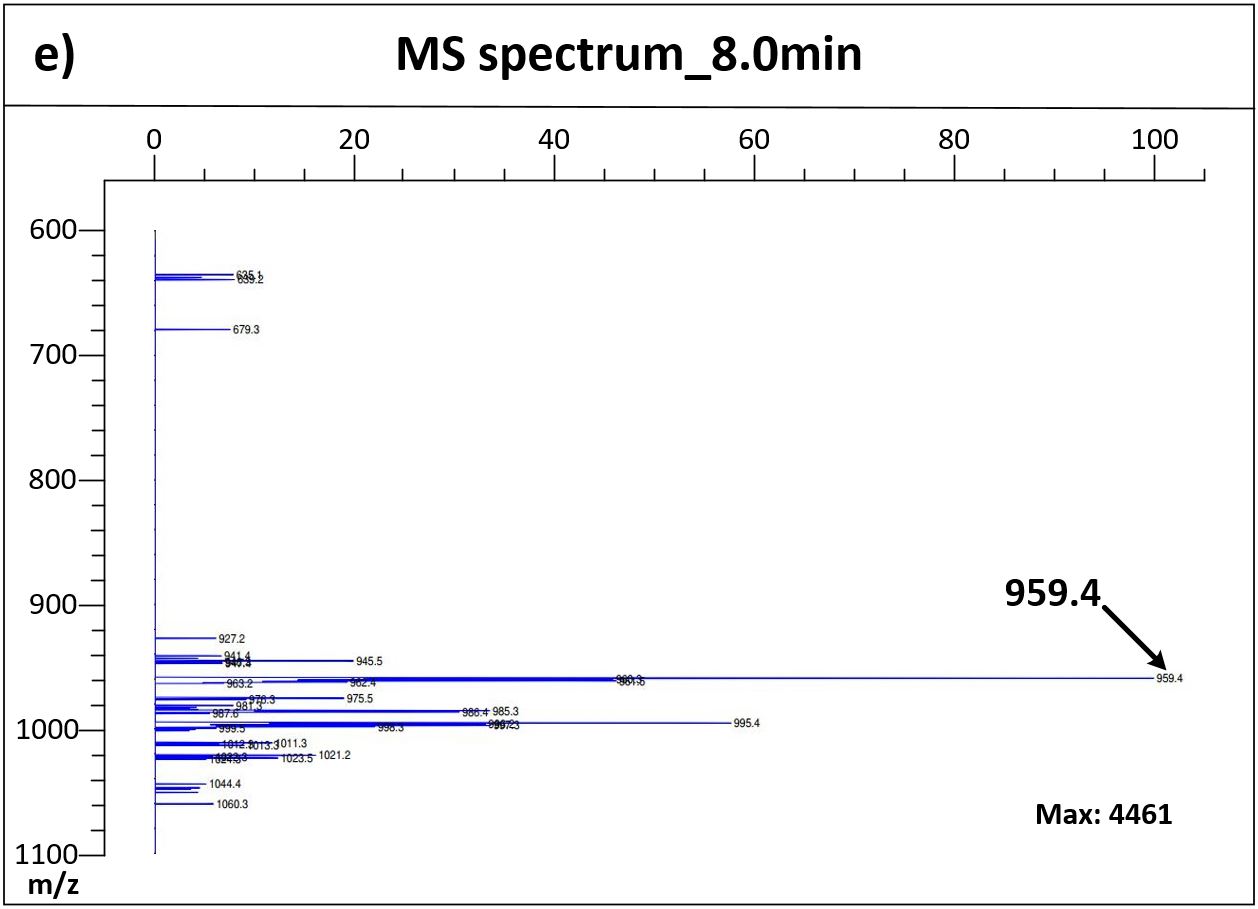


e). Mass spectrum of fractions of peak at RT of 8.0min indicated mono-Ac bola SL (C16:1) with molecular mass of 960.


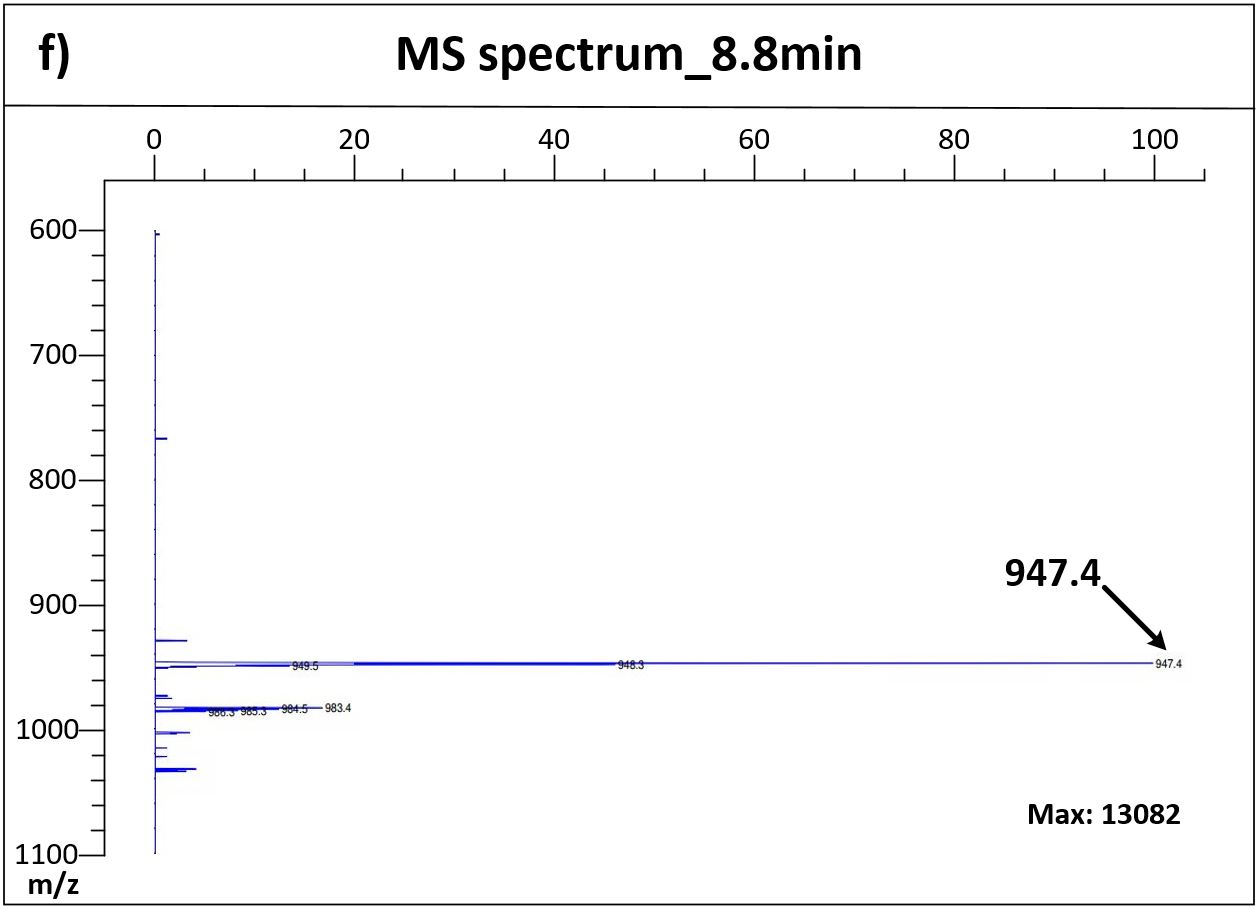


f). Mass spectrum of fractions of peak at RT of 8.8min indicated non-Ac bola SL (C18:0) with molecular mass of 948.


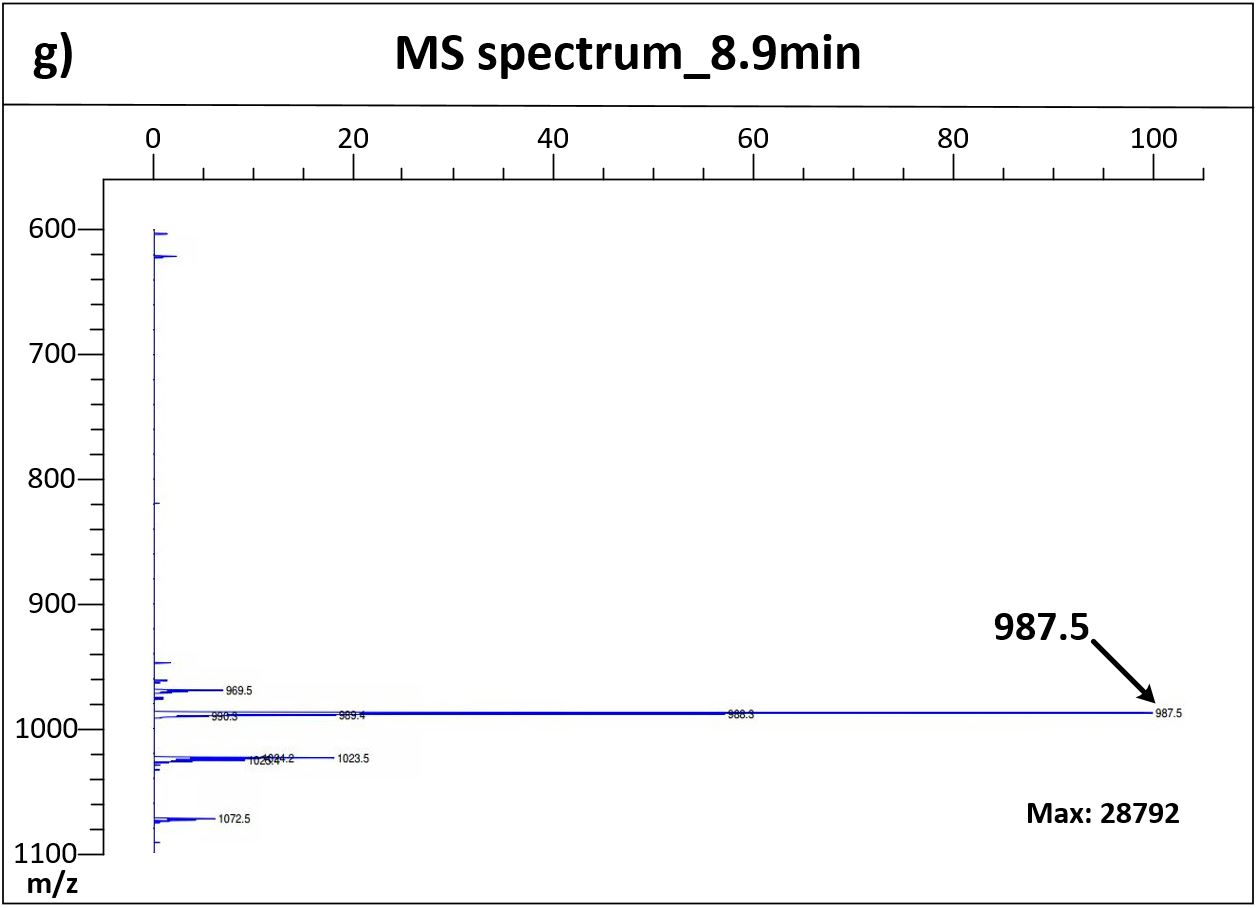


g). Mass spectrum of fractions of peak at RT of 8.9min indicated mono-Ac bola SL (C18:1) with molecular mass of 988.


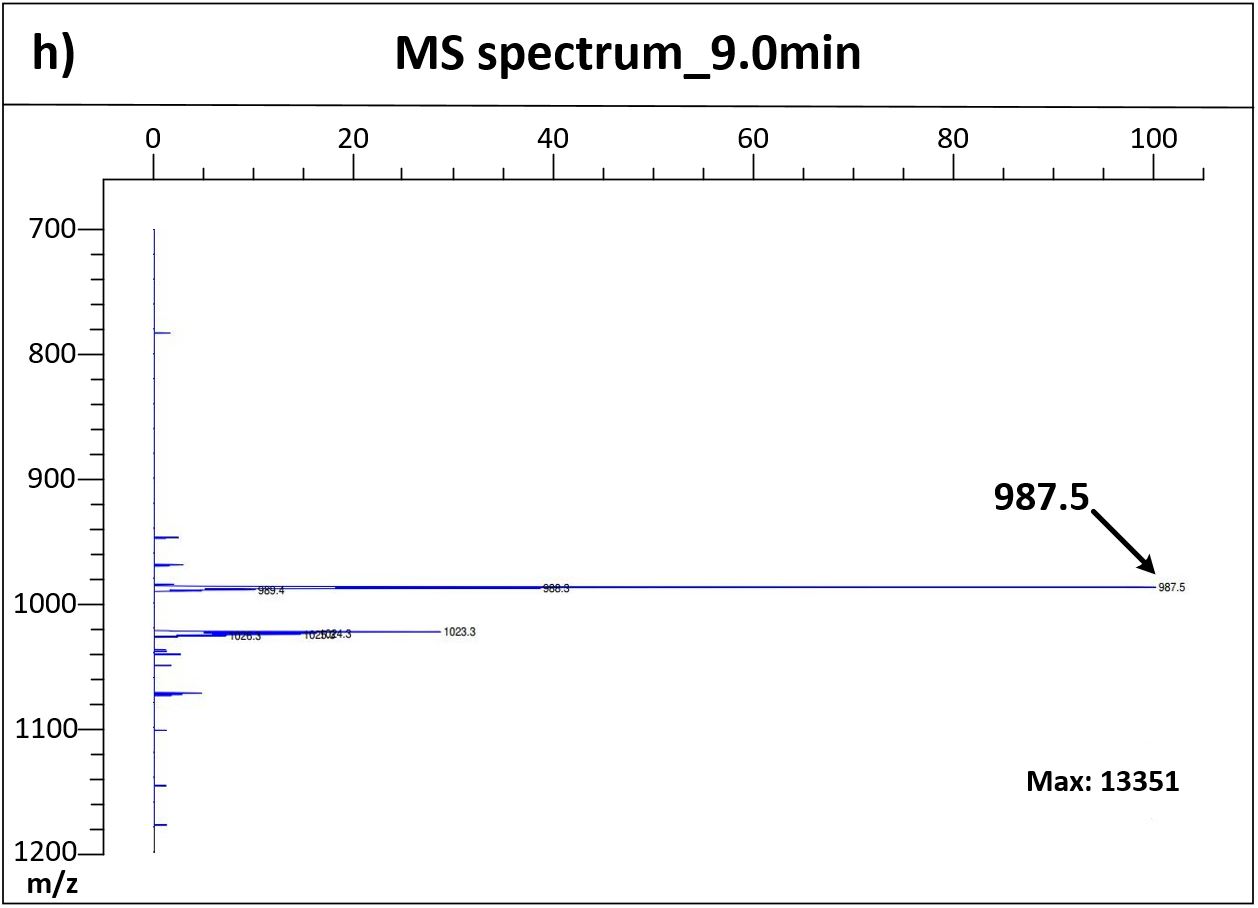


h). Mass spectrum of fractions of peak at RT of 9.0min indicated mono-Ac bola SL (C18:1) with molecular mass of 988.


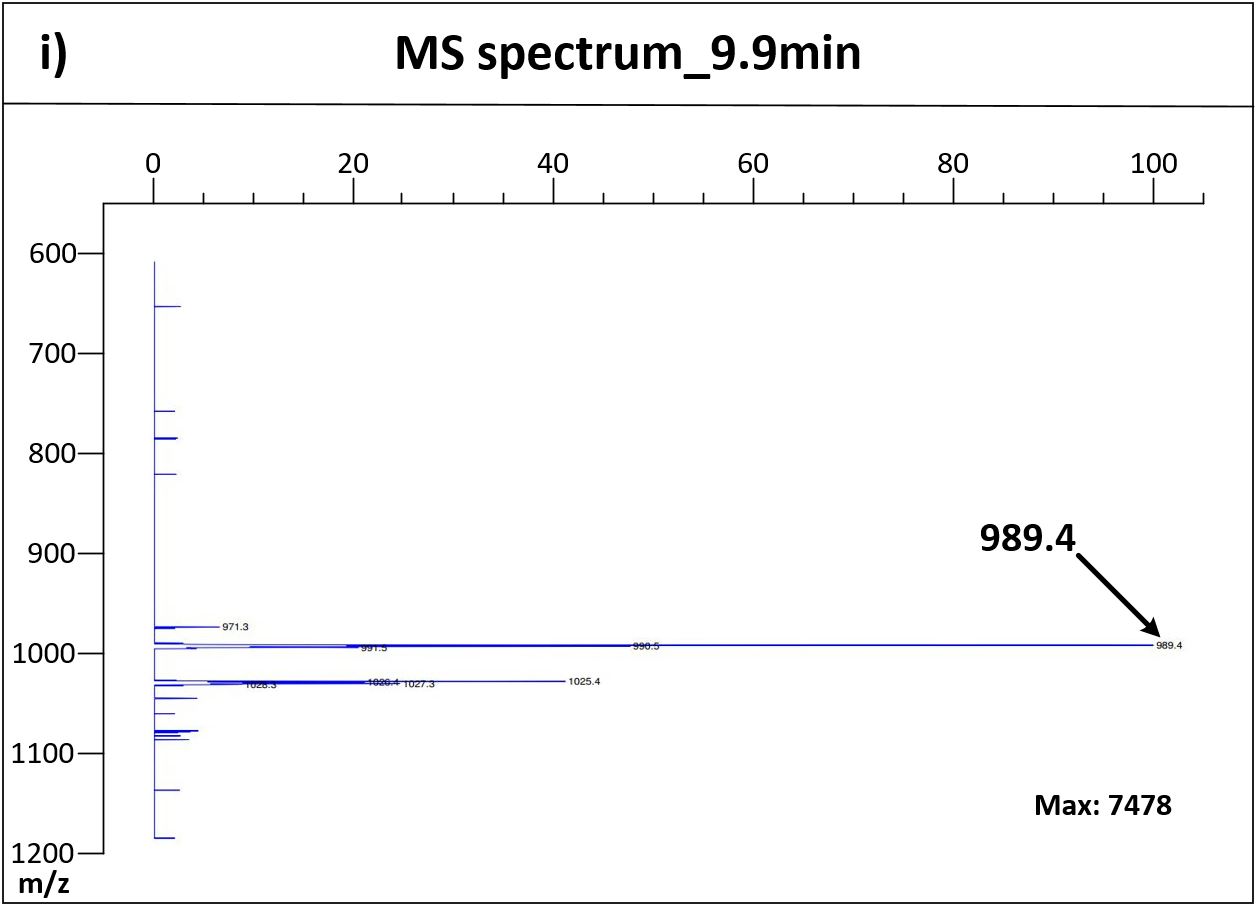


i). Mass spectrum of fractions of peak at RT of 9.9min indicated mono-Ac bola SL (C18:0) with molecular mass of 990.


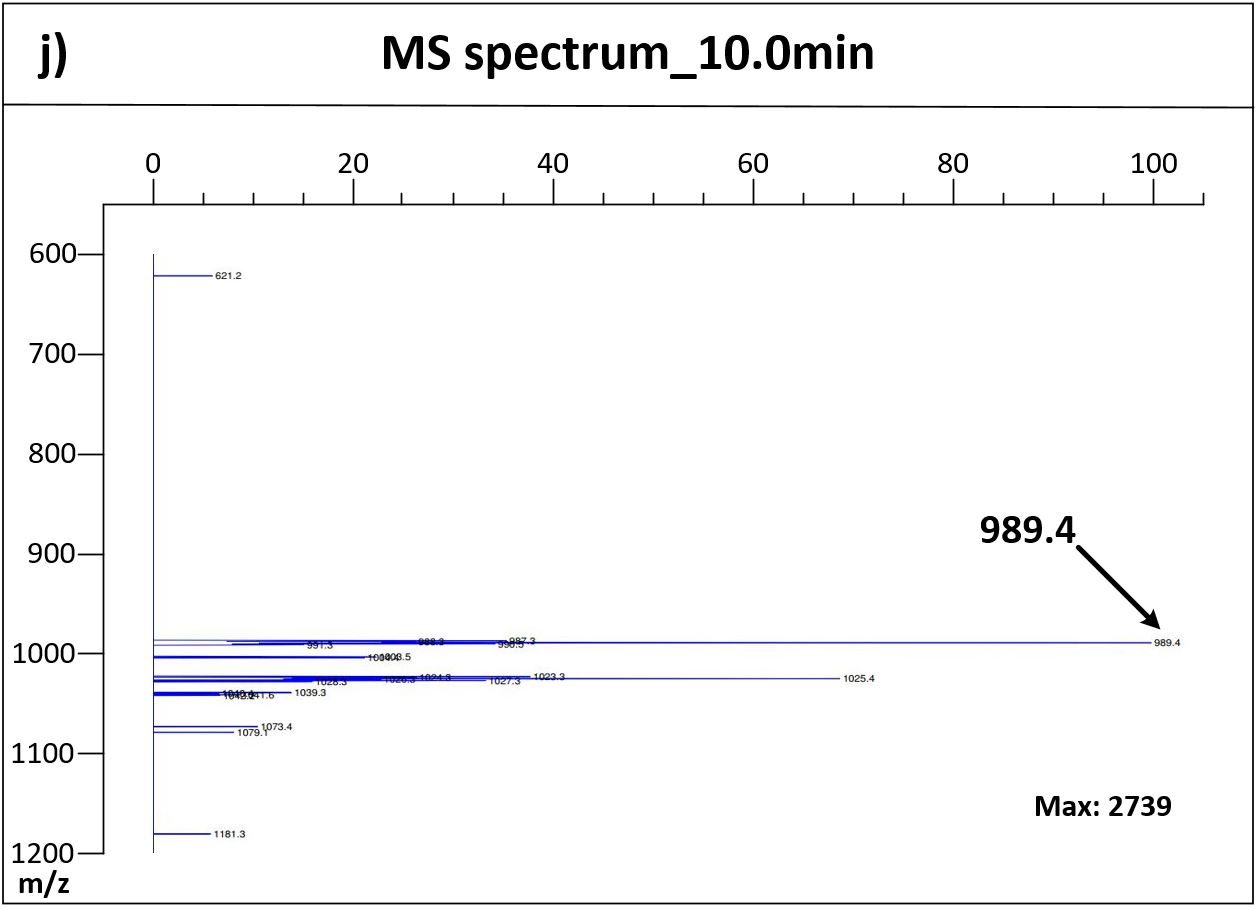


j). Mass spectrum of fractions of peak at RT of 10.0min indicated mono-Ac bola SL (C18:0) with molecular mass of 990.


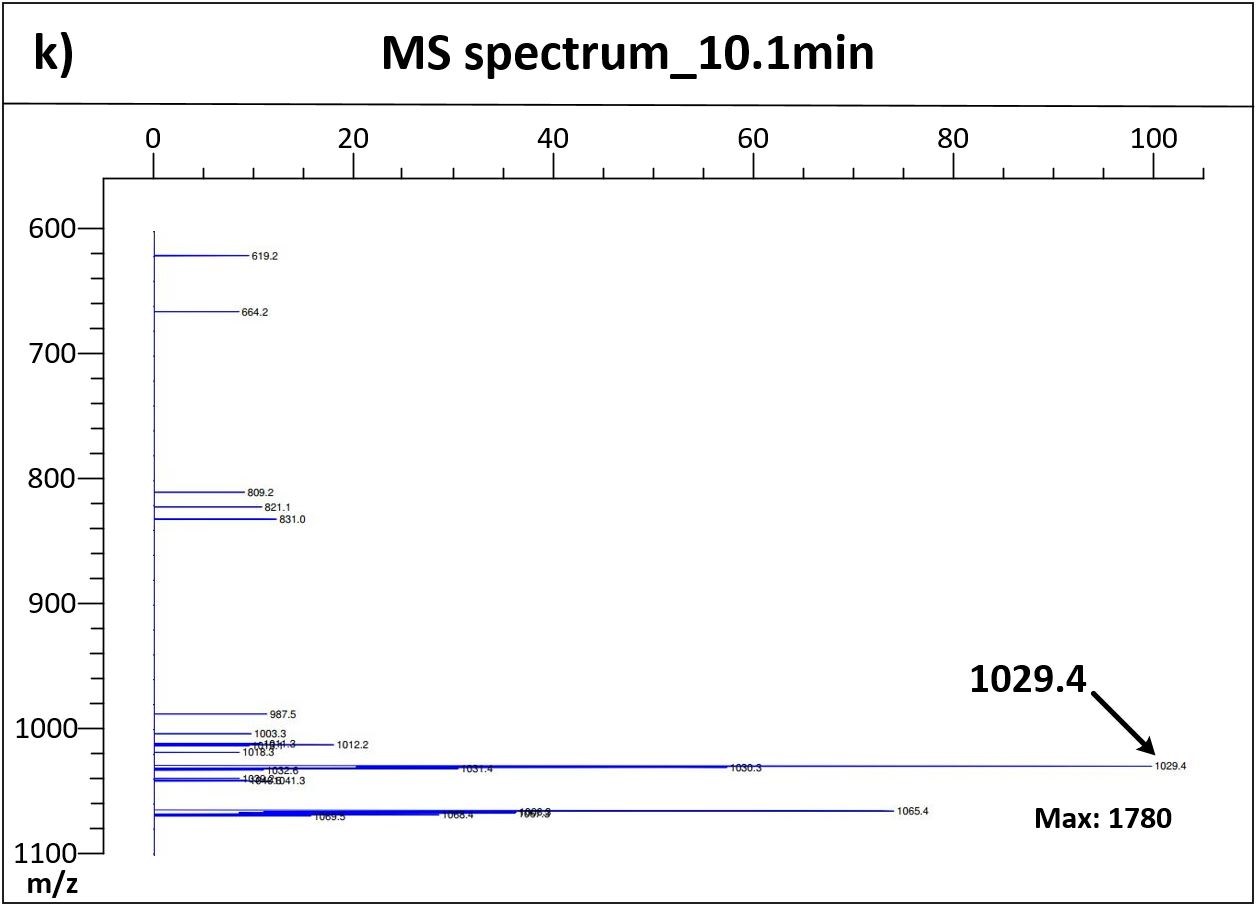


k). Mass spectrum of fractions of peak at RT of 10.1min indicated di-Ac bola SL (C18:1) with molecular mass of 1030.


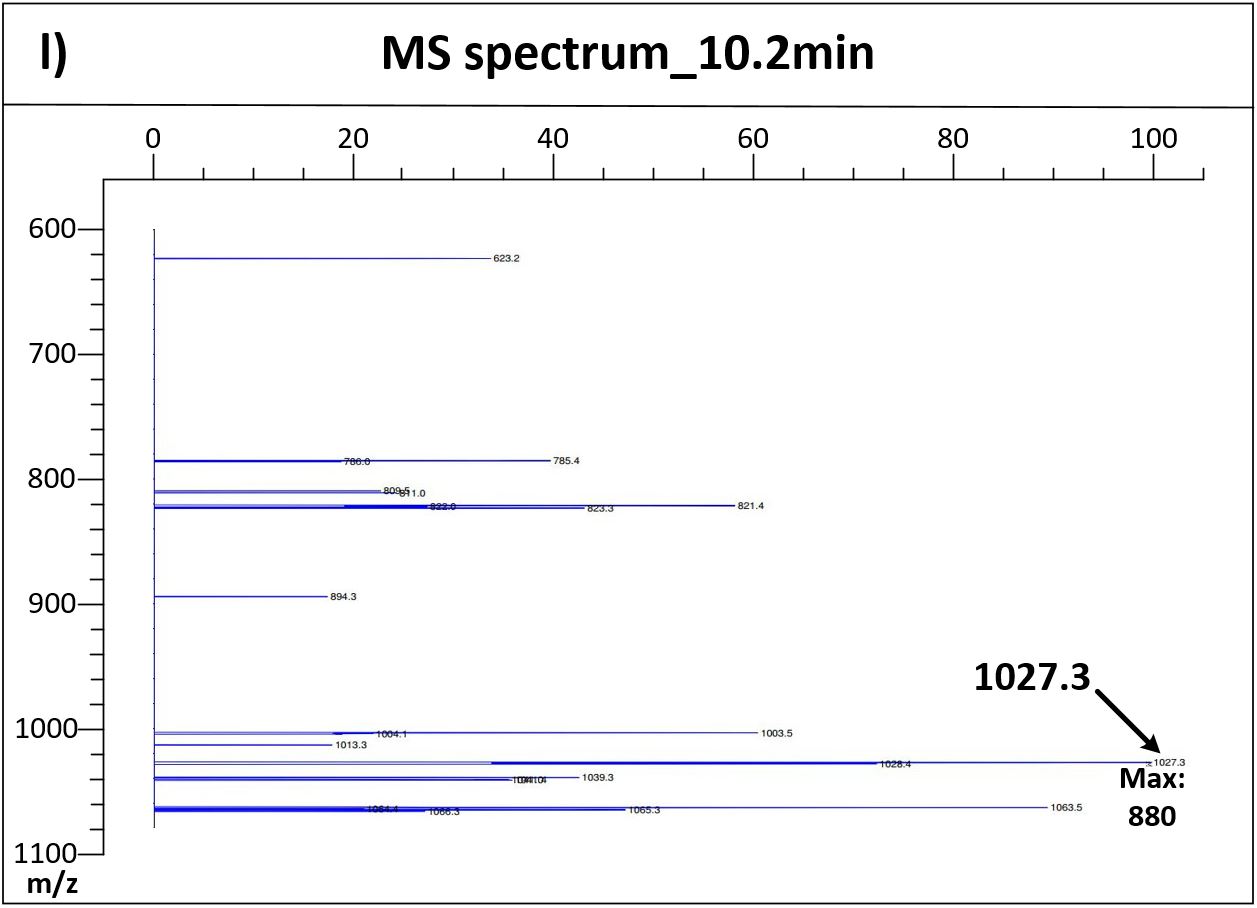


l). Mass spectrum of fractions of peak at RT of 10.2min indicated di-Ac bola SL (C18:2) with molecular mass of 1028.


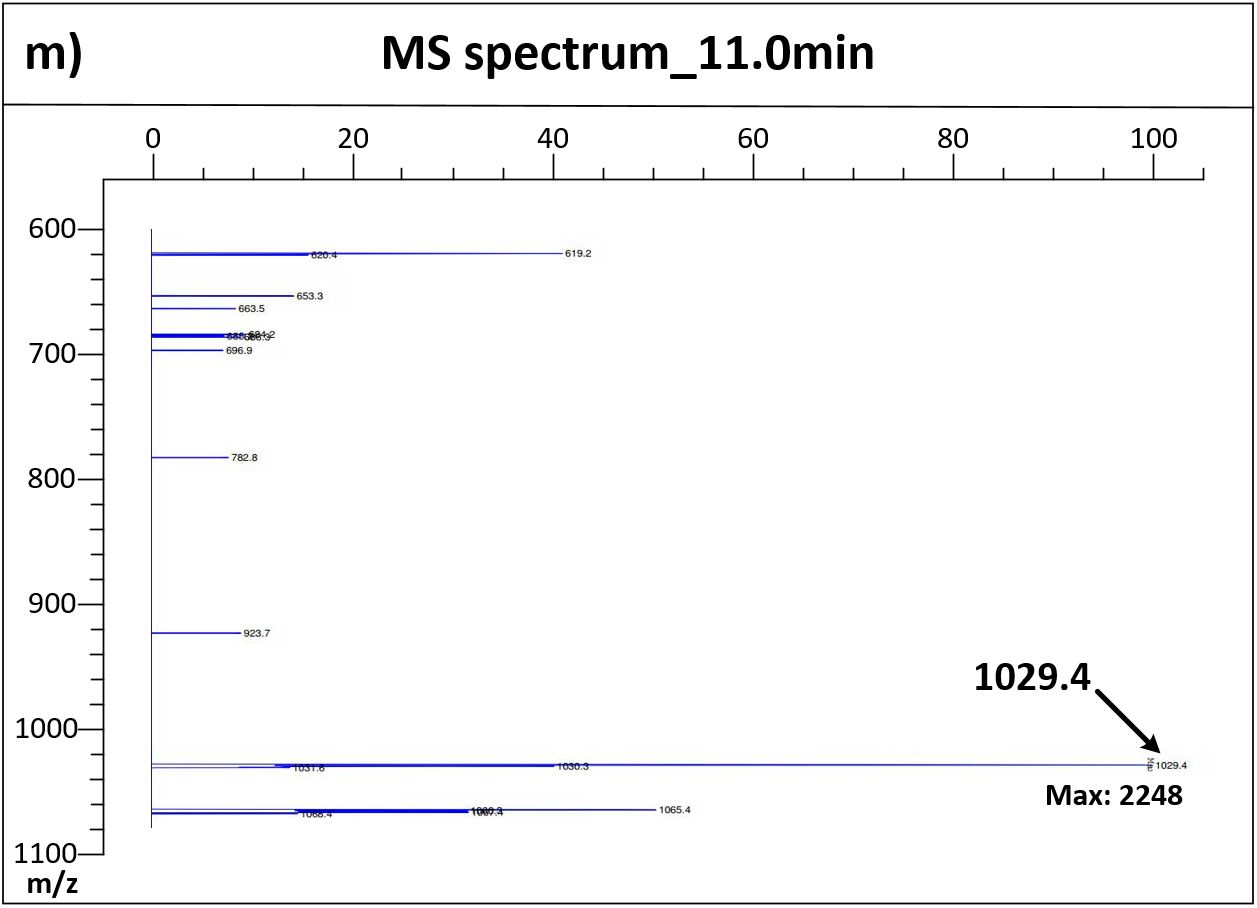


m). Mass spectrum of fractions of peak at RT of 11.0min indicated di-Ac bola SL (C18:1) with molecular mass of 1030.


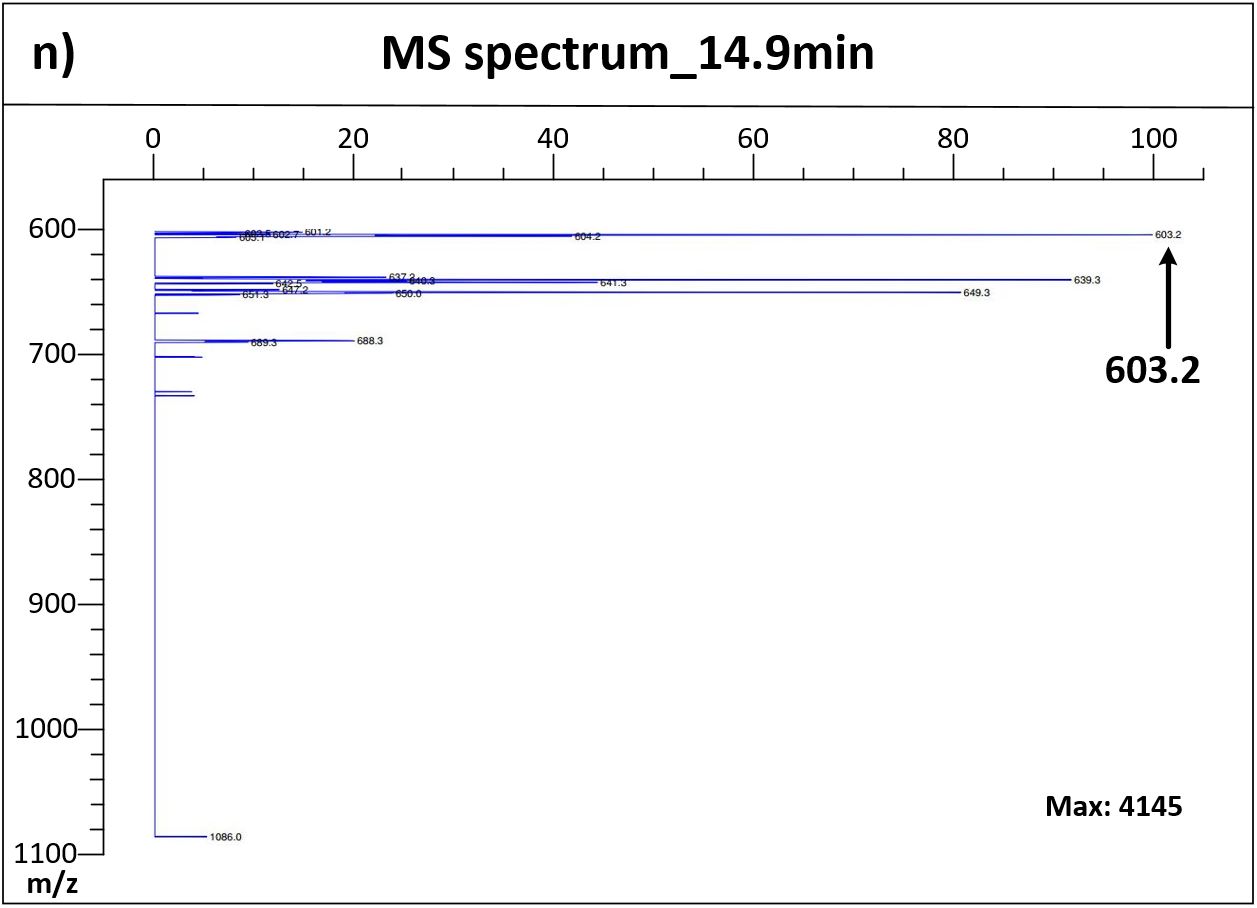


n): Mass spectrum of fractions of peak at RT of 14.9min indicated non-Ac lactonic SL (C18:1) with molecular mass of 604.


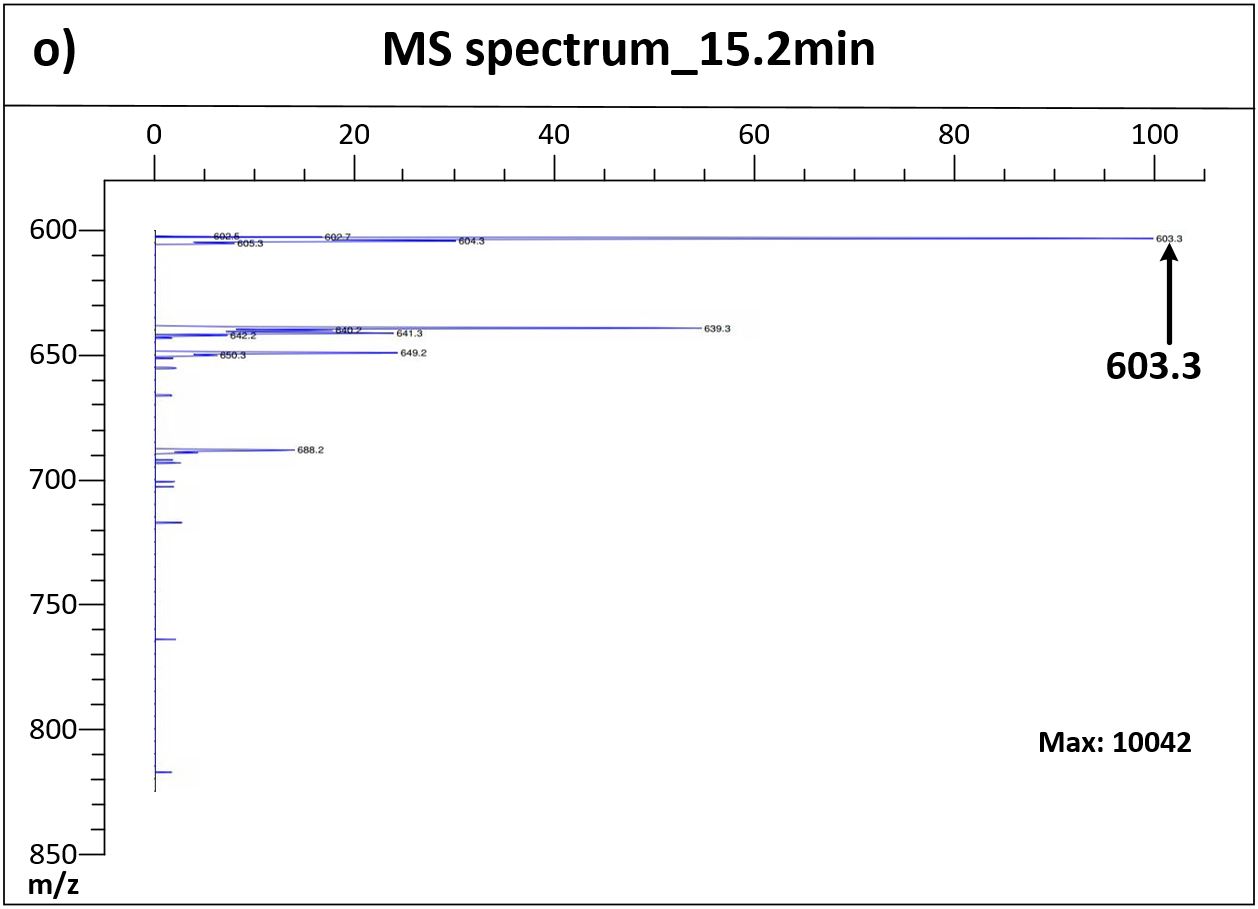


o). Mass spectrum of fractions of peak at RT of 15.2min indicated non-Ac lactonic SL (C18:1) with molecular mass of 604.


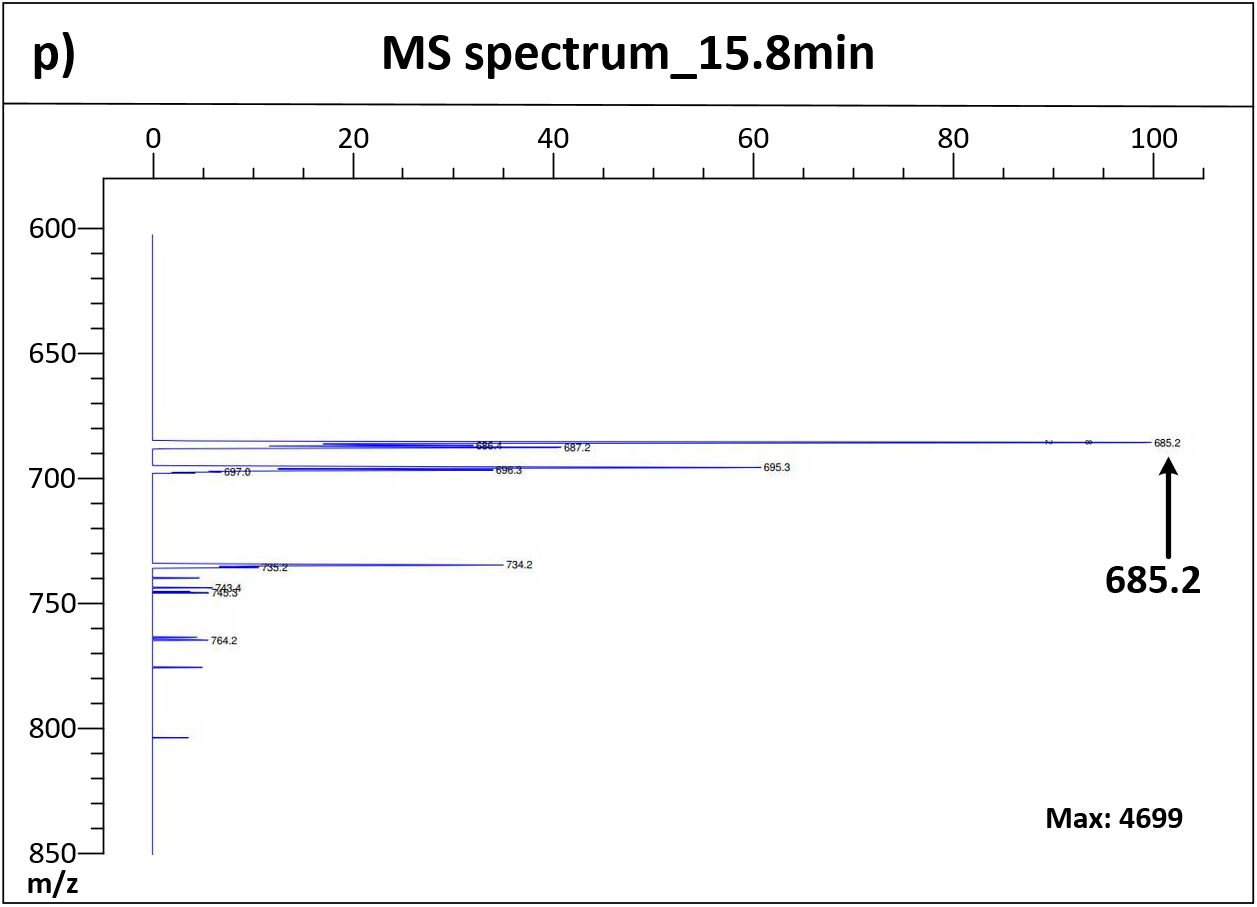


p). Mass spectrum of fractions of peak at RT of 15.8min indicated di-Ac lactonic SL (C18:2) with molecular mass of 686.


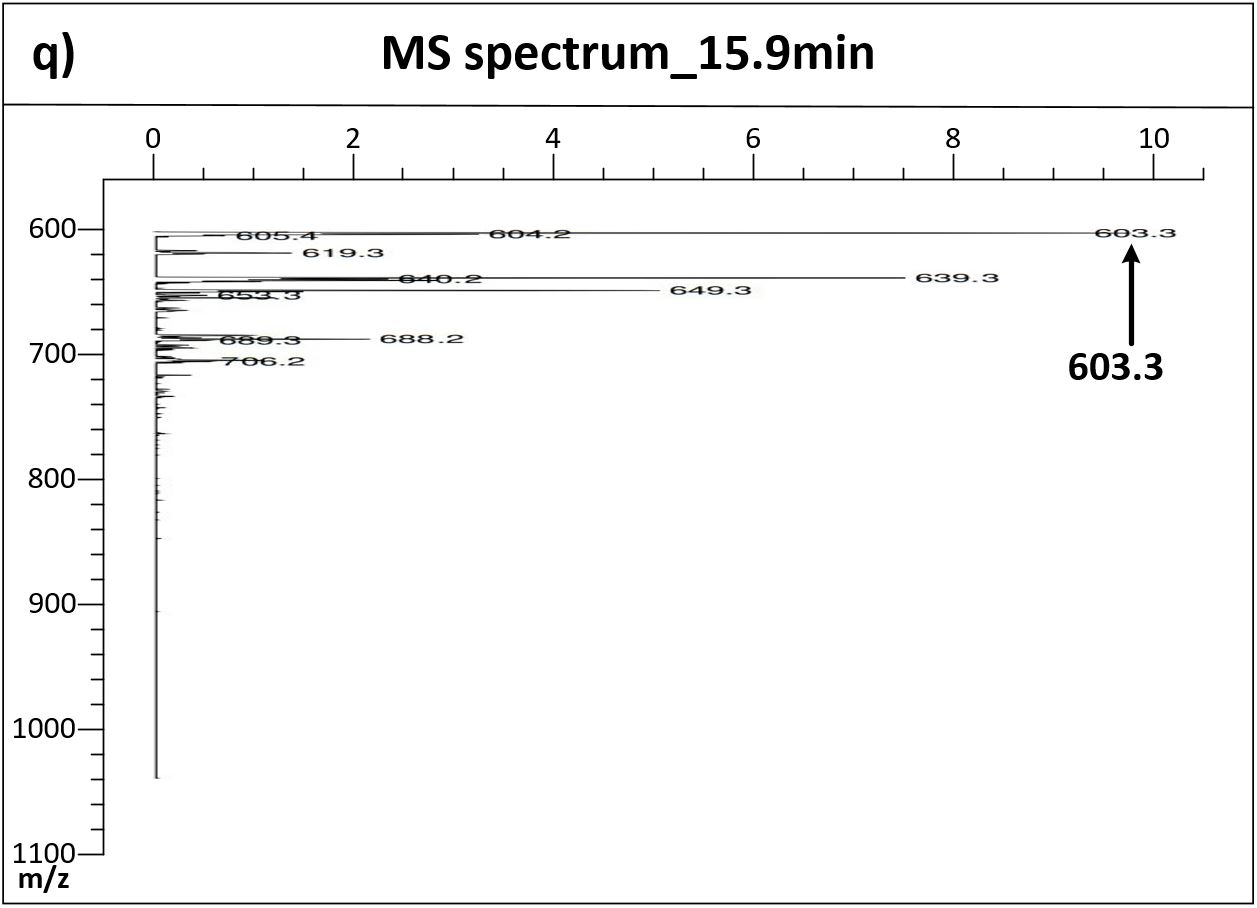


q). Mass spectrum of fractions of peak at RT of 15.9min indicated non-Ac lactonic SL (C18:1) with molecular mass of 604.


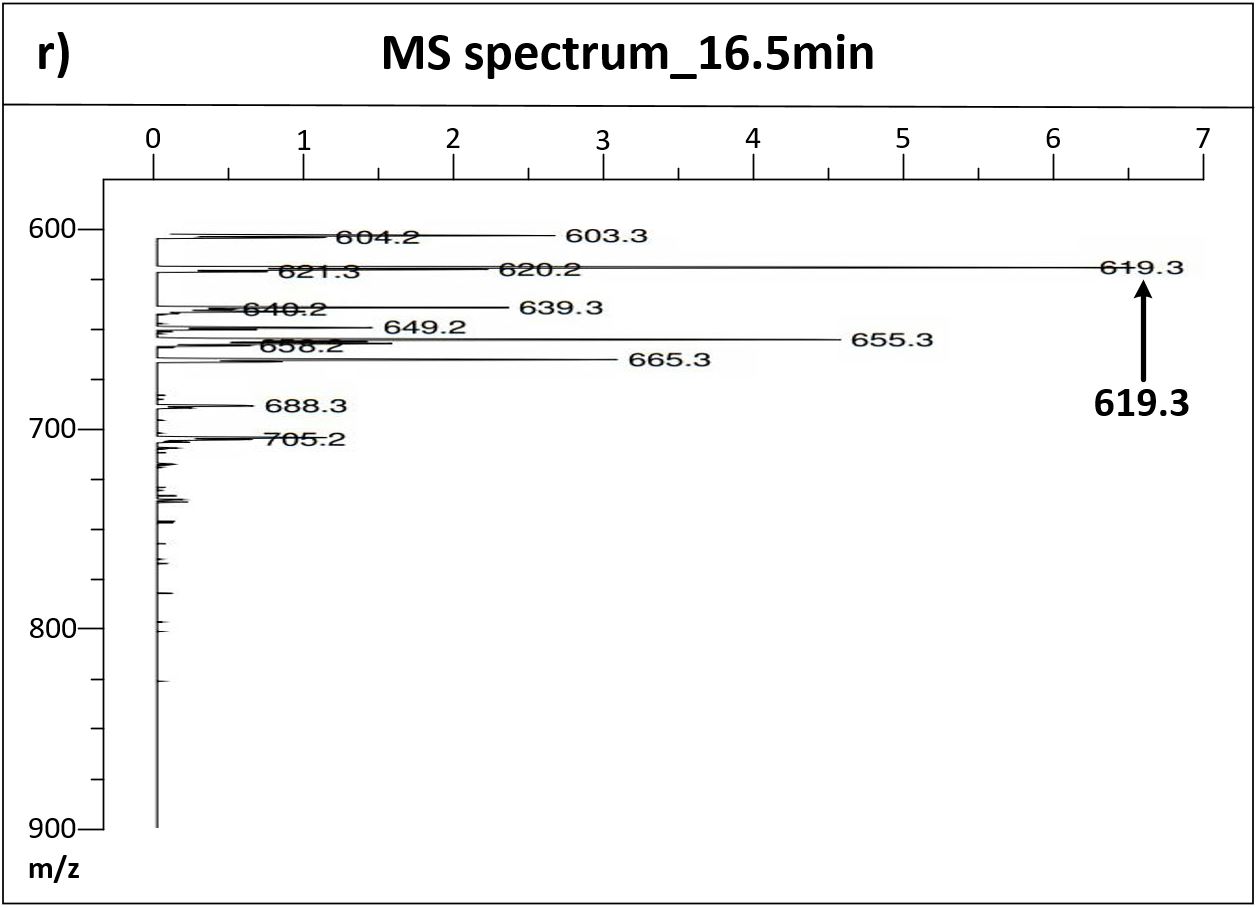


r). Mass spectrum of fractions of peak at RT of 16.5min indicated mono-Ac lactonic SL (C16:0) with molecular mass of 620.


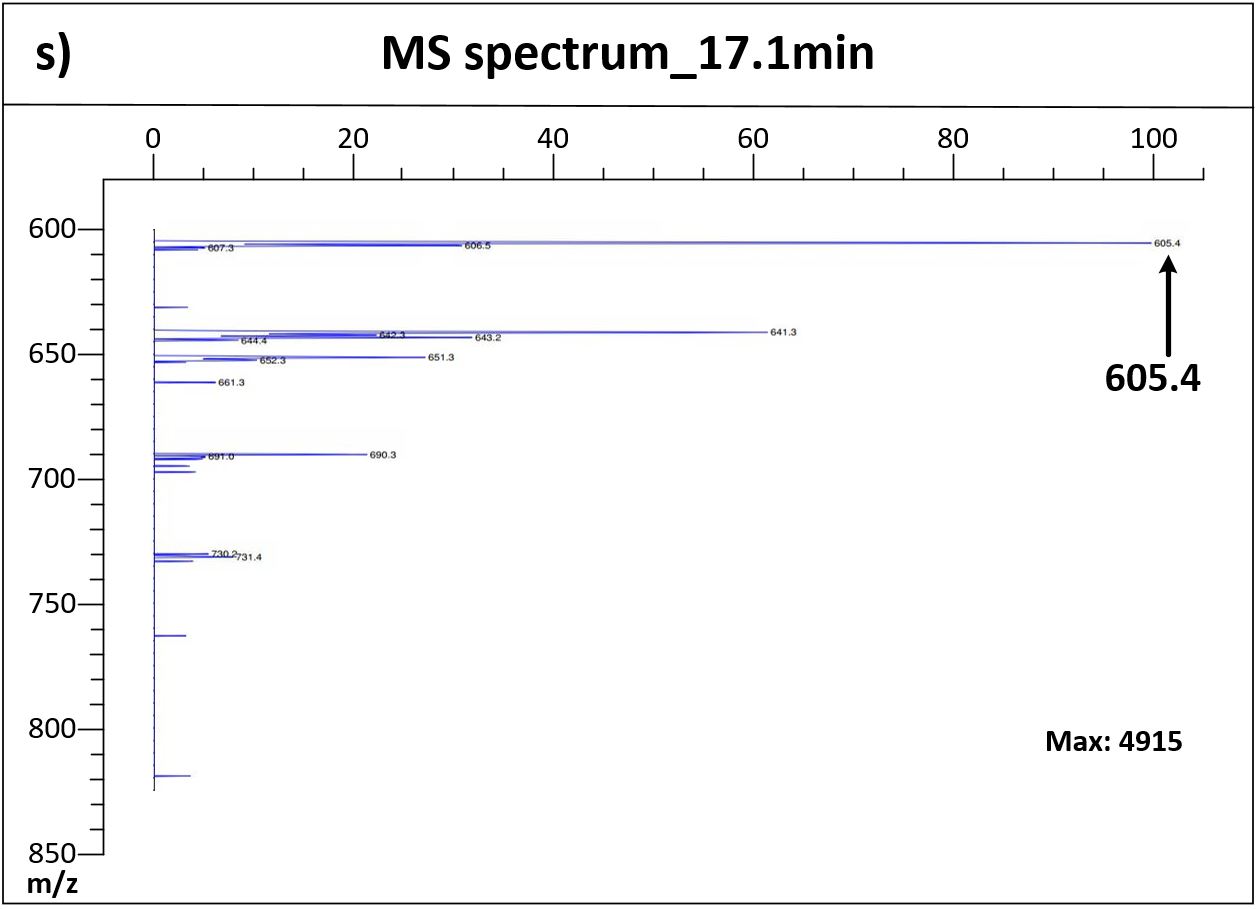


s). Mass spectrum of fractions of peak at RT of 17.1min indicated non-Ac lactonic SL (C18:0) with molecular mass of 606.


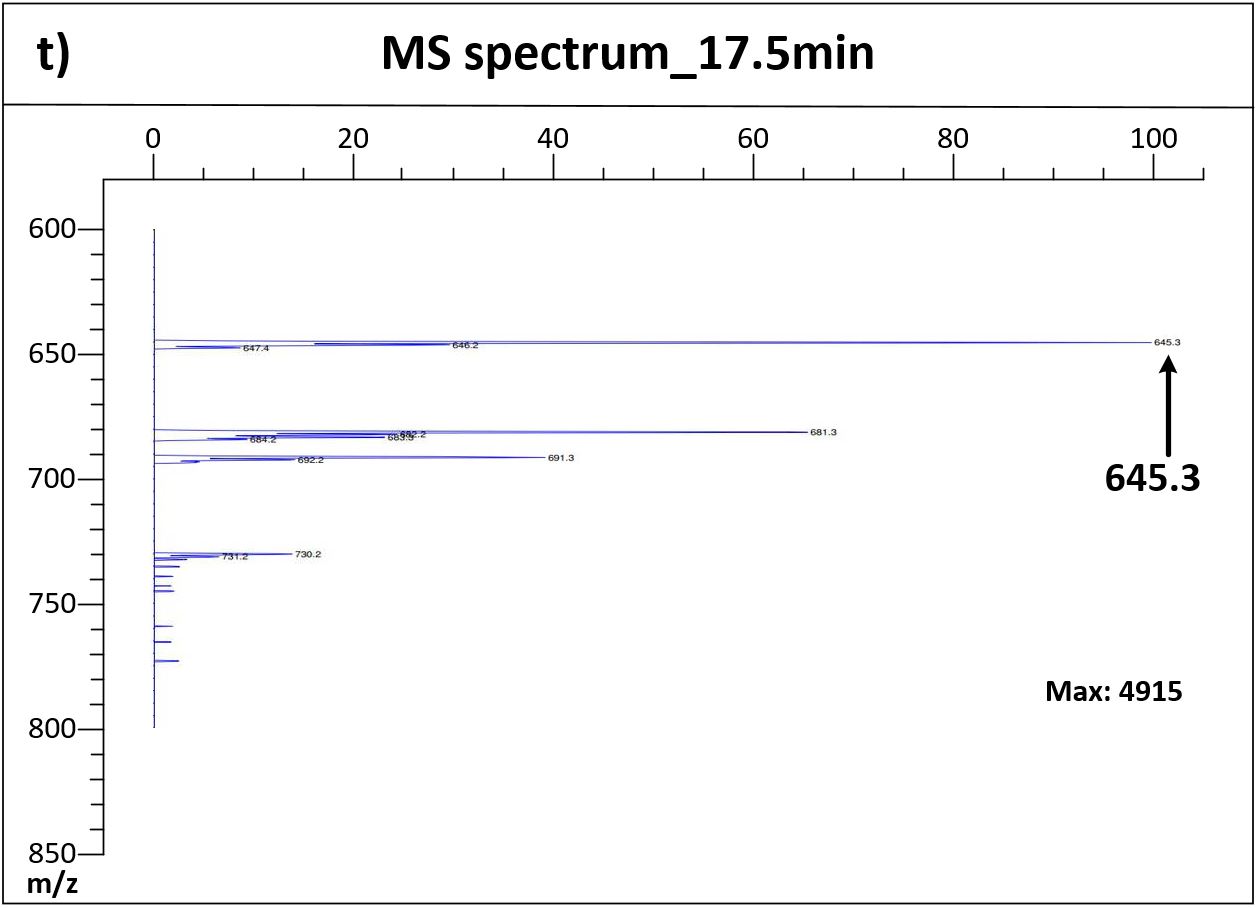


t). Mass spectrum of fractions of peak at RT of 17.5min indicated mono-Ac lactonic SL (C18:1) with molecular mass of 646.


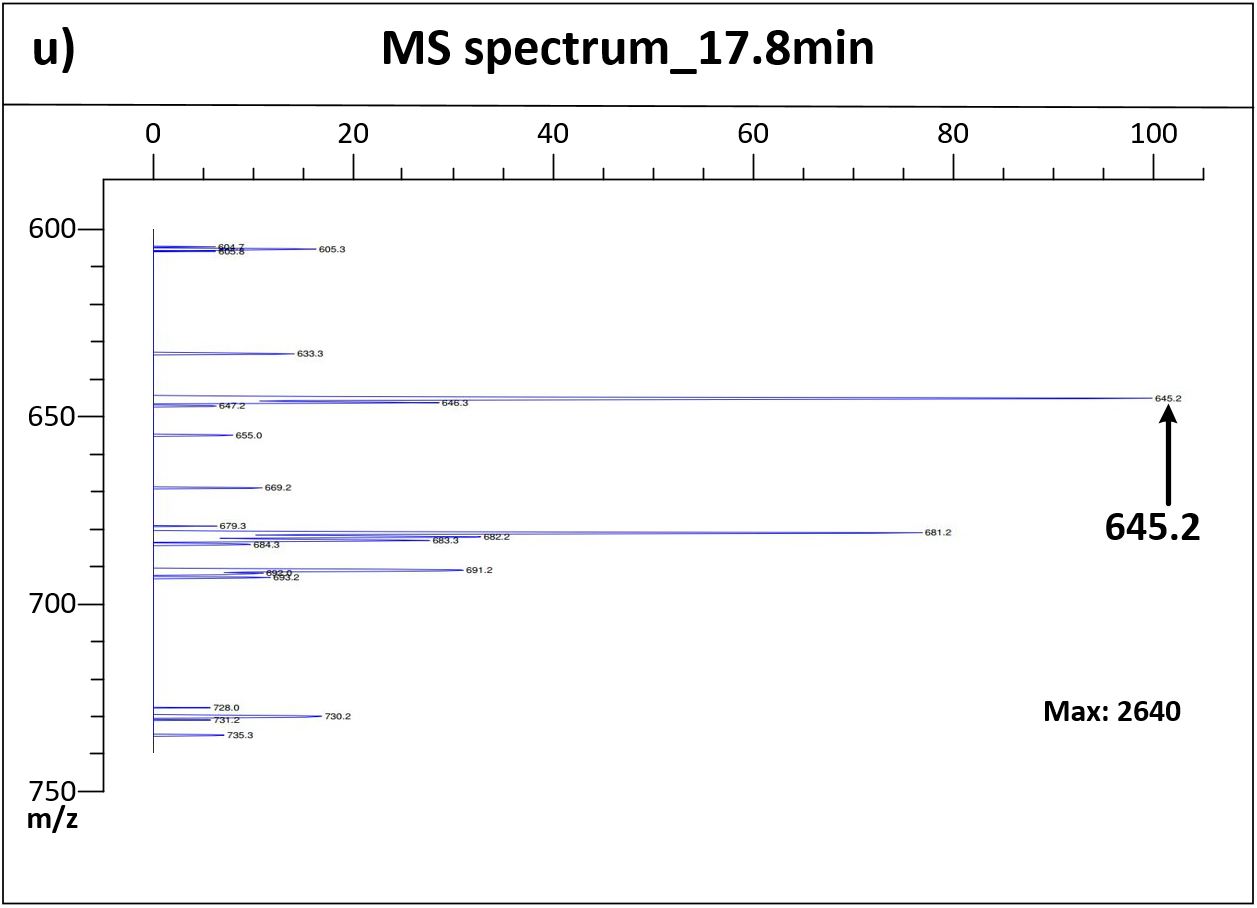


u). Mass spectrum of fractions of peak at RT of 17.8min indicated mono-Ac lactonic SL (C18:1) with molecular mass of 646.


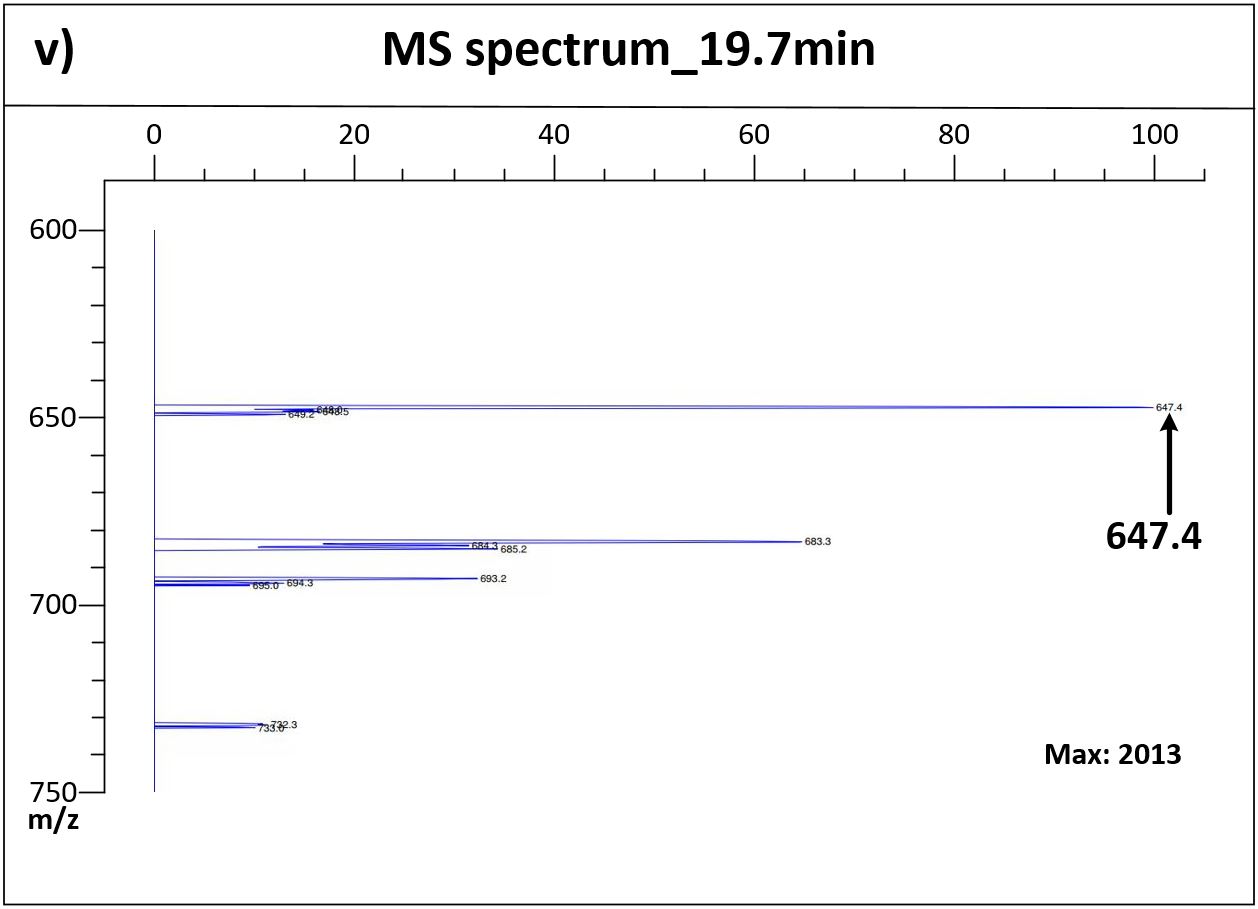


v). Mass spectrum of fractions of peak at RT of 19.7min indicated mono-Ac lactonic SL (C18:0) with molecular mass of 648.


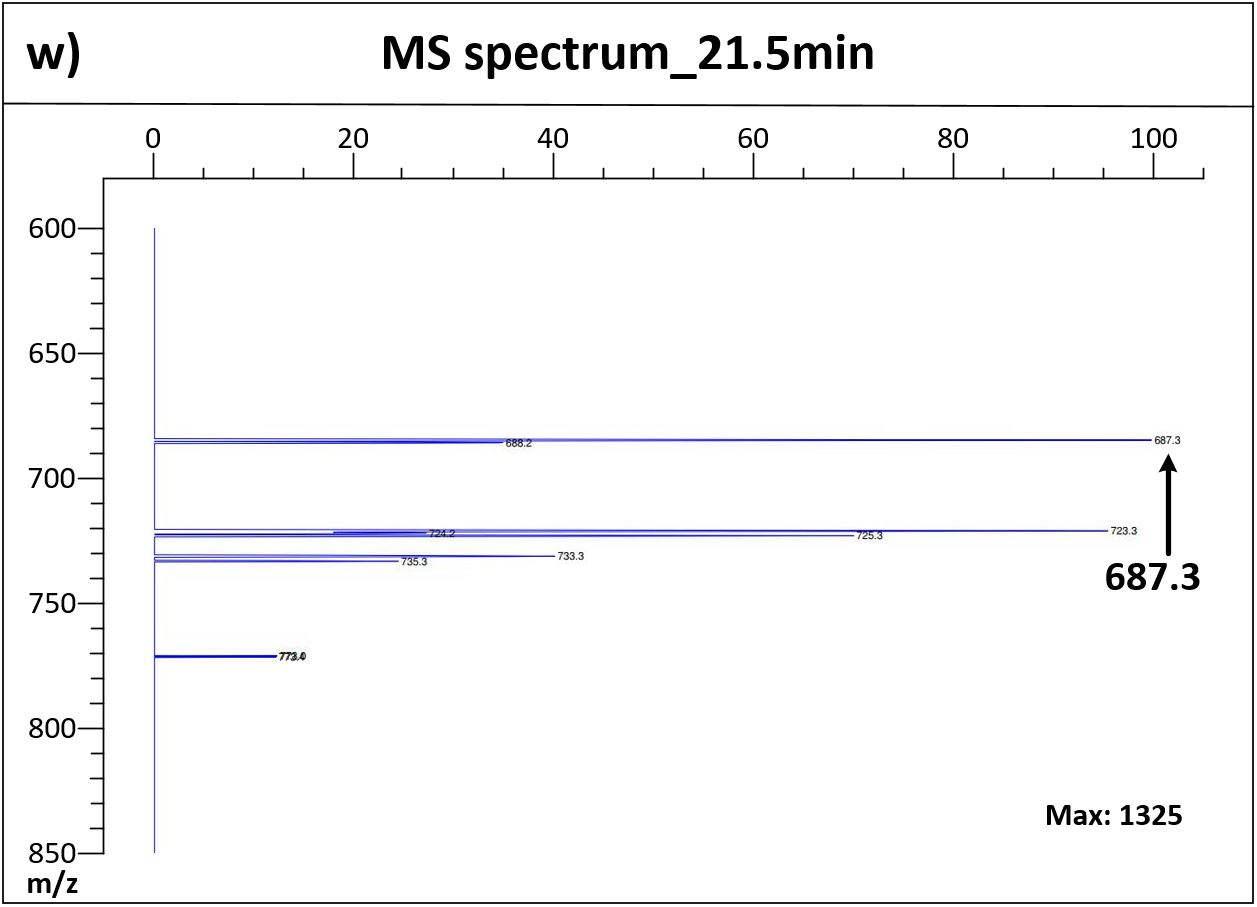


w). Mass spectrum of fractions of peak at RT of 21.5min indicated di-Ac lactonic SL (C18:1) with molecular mass of 688.


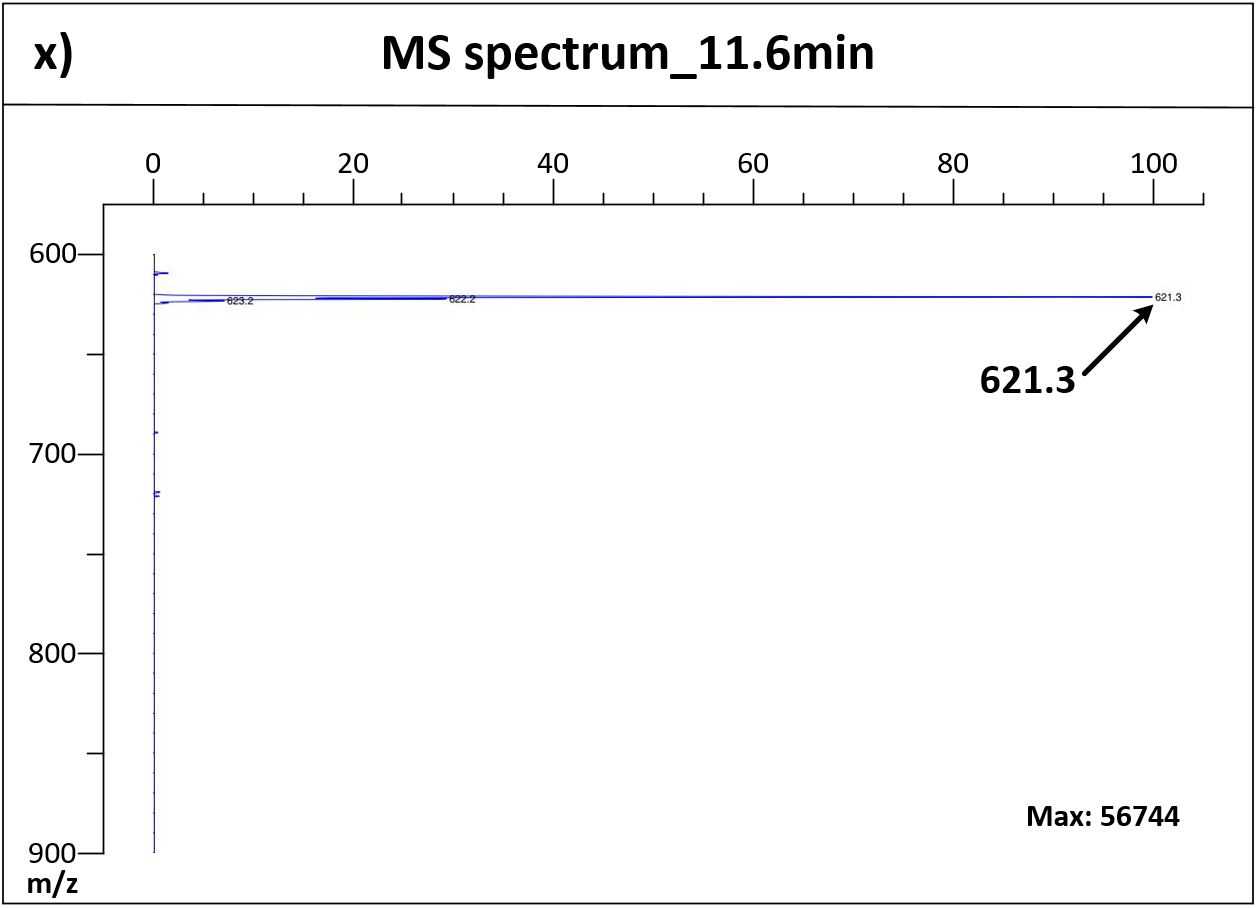


x) Mass spectrum of fractions of peak at RT of 11.6min indicated non-Ac acidic SL (C18:1) with molecular mass of 622.
