# Supplementary figures and images for: Revision of the sophorolipid biosynthetic pathway in Starmerella bombicola based on new insights in the substrate profile of its lactone esterase
Source: Biotechnol Biofuels Bioprod. 2024 Jun 27;17:89. doi: 10.1186/s13068-024-02533-1 (PMC11210130; doi:10.1186/s13068-024-02533-1)

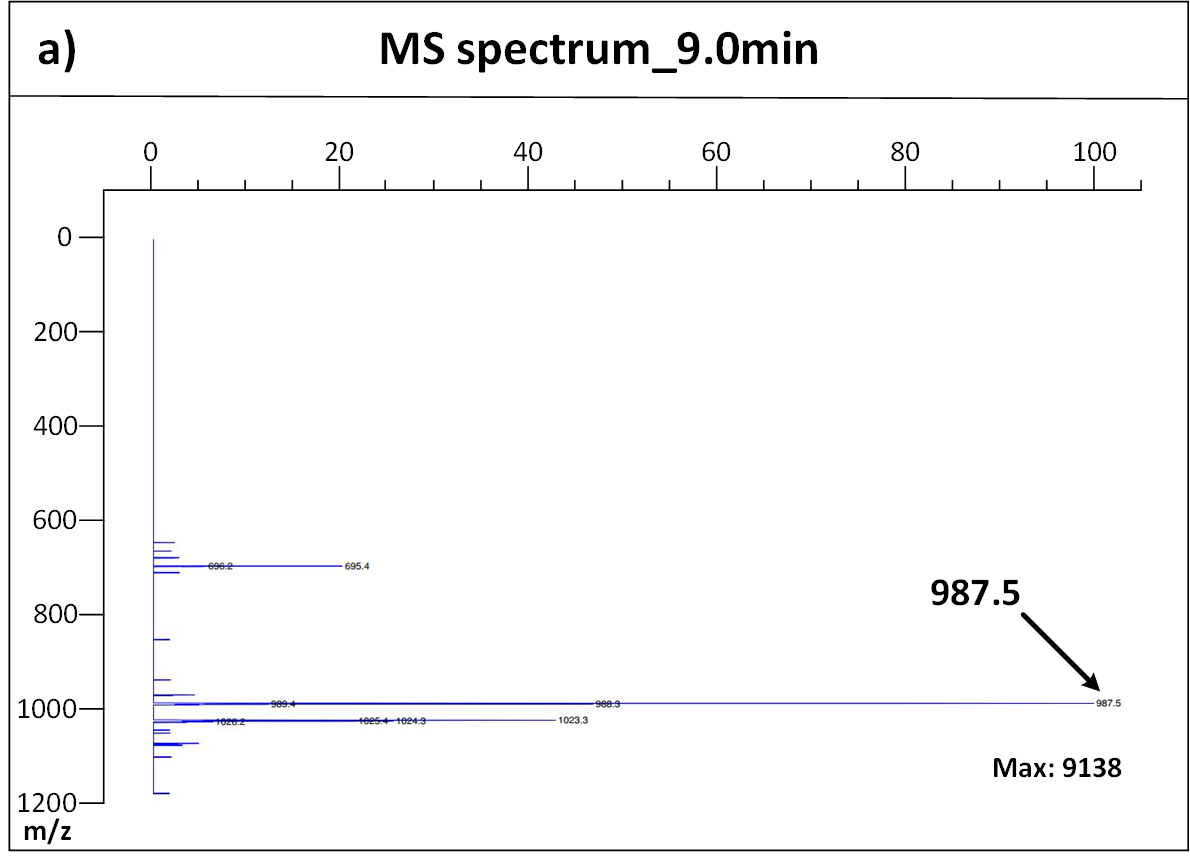

Supplement: Supplementary file 3 — Supplementary Material 3. Figure S3. LC–MS spectra of sophorolipid products obtained after incubation of bola SLs with rSBLE. [file 13068_2024_2533_MOESM3_ESM.zip › Fig. S3/Fig S3 a).png]

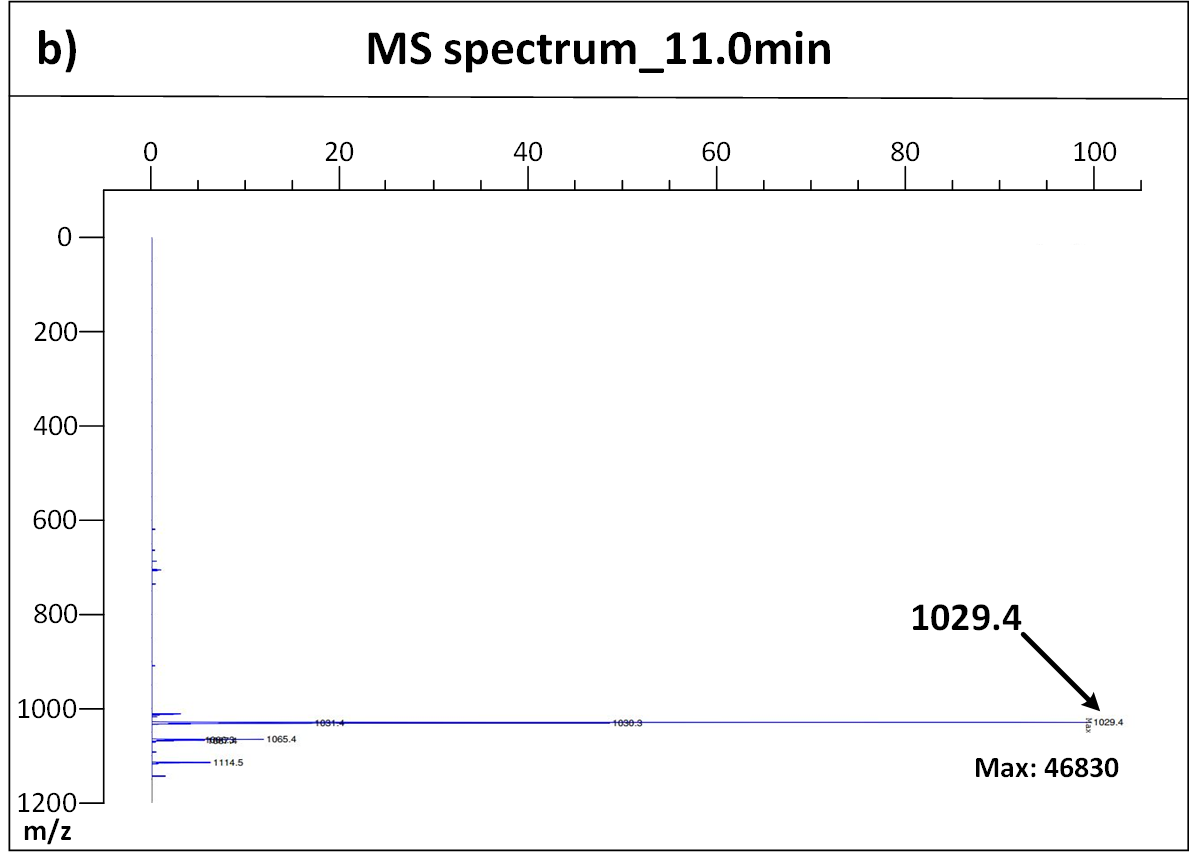

Supplement: Supplementary file 3 — Supplementary Material 3. Figure S3. LC–MS spectra of sophorolipid products obtained after incubation of bola SLs with rSBLE. [file 13068_2024_2533_MOESM3_ESM.zip › Fig. S3/Fig S3 b).png]

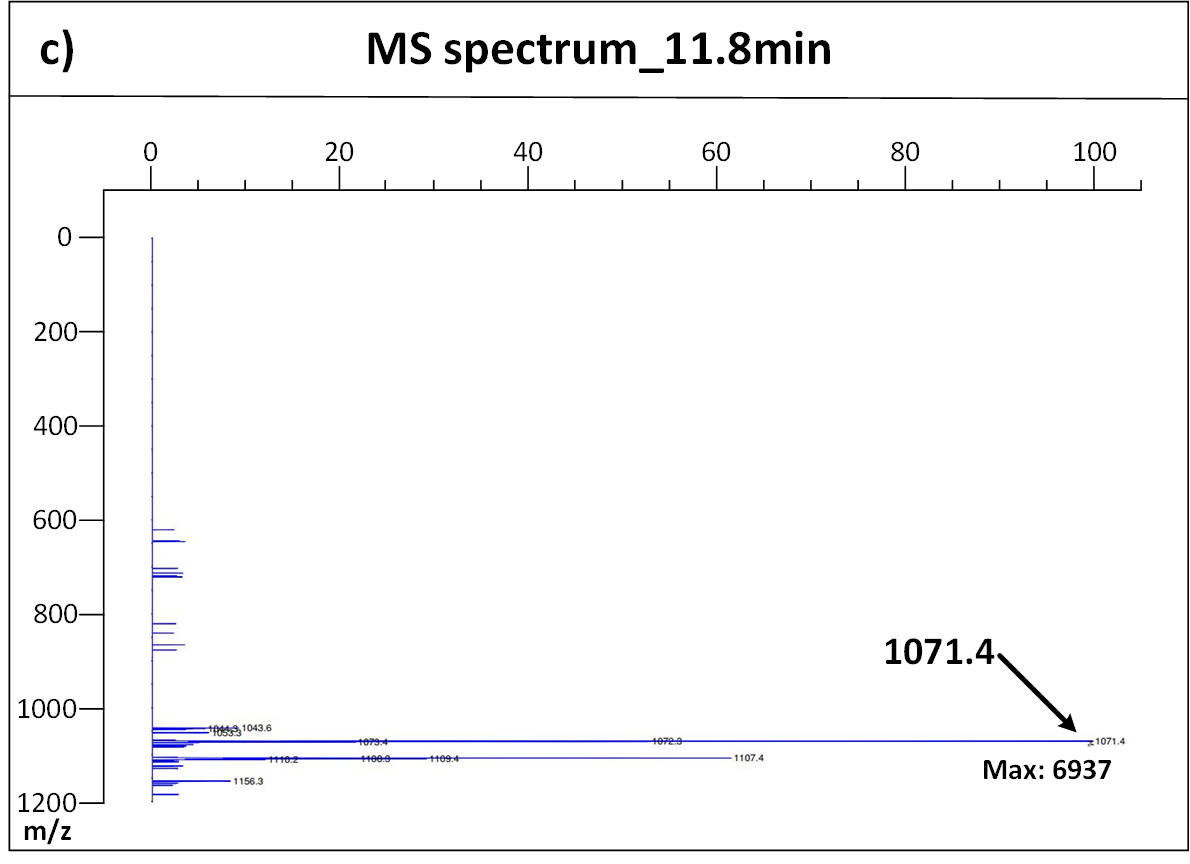

Supplement: Supplementary file 3 — Supplementary Material 3. Figure S3. LC–MS spectra of sophorolipid products obtained after incubation of bola SLs with rSBLE. [file 13068_2024_2533_MOESM3_ESM.zip › Fig. S3/Fig S3 c).png]

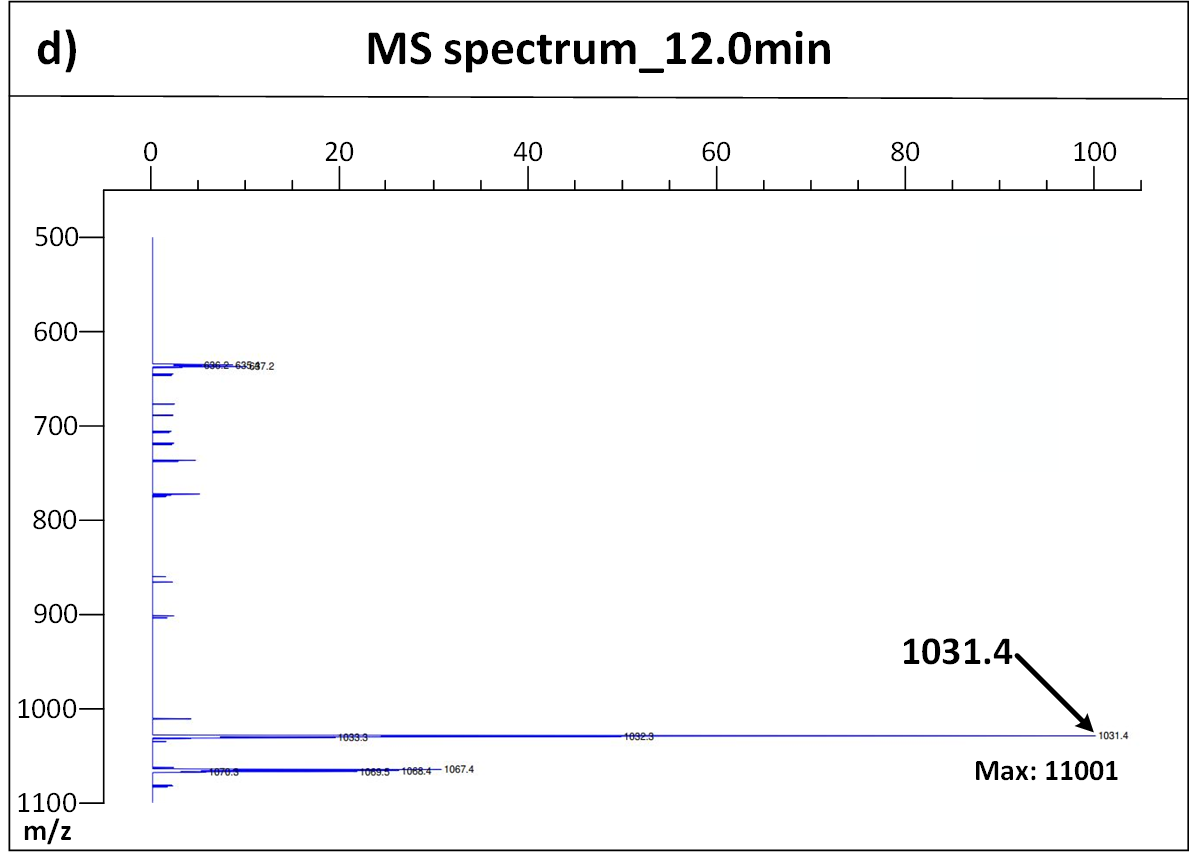

Supplement: Supplementary file 3 — Supplementary Material 3. Figure S3. LC–MS spectra of sophorolipid products obtained after incubation of bola SLs with rSBLE. [file 13068_2024_2533_MOESM3_ESM.zip › Fig. S3/Fig S3 d).png]

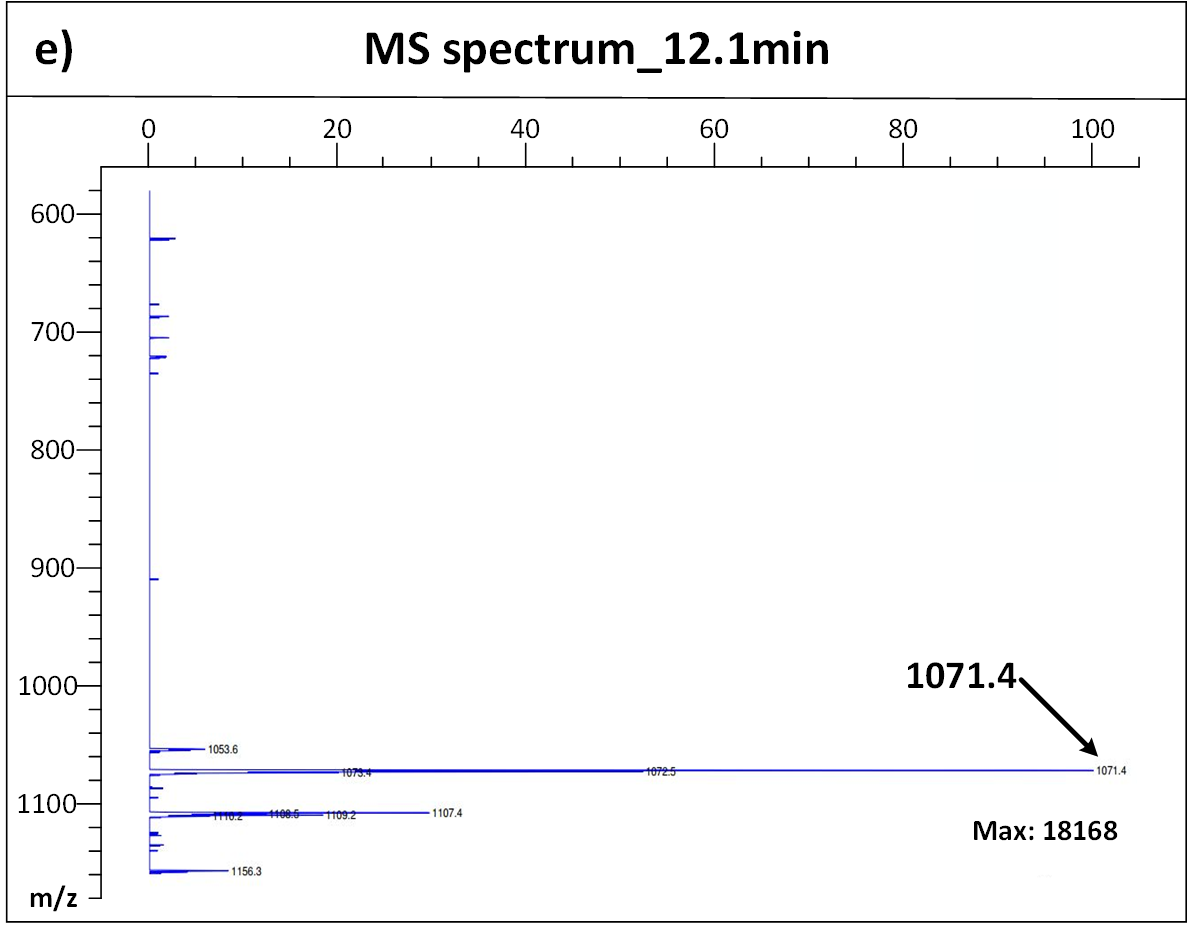

Supplement: Supplementary file 3 — Supplementary Material 3. Figure S3. LC–MS spectra of sophorolipid products obtained after incubation of bola SLs with rSBLE. [file 13068_2024_2533_MOESM3_ESM.zip › Fig. S3/Fig S3 e).png]

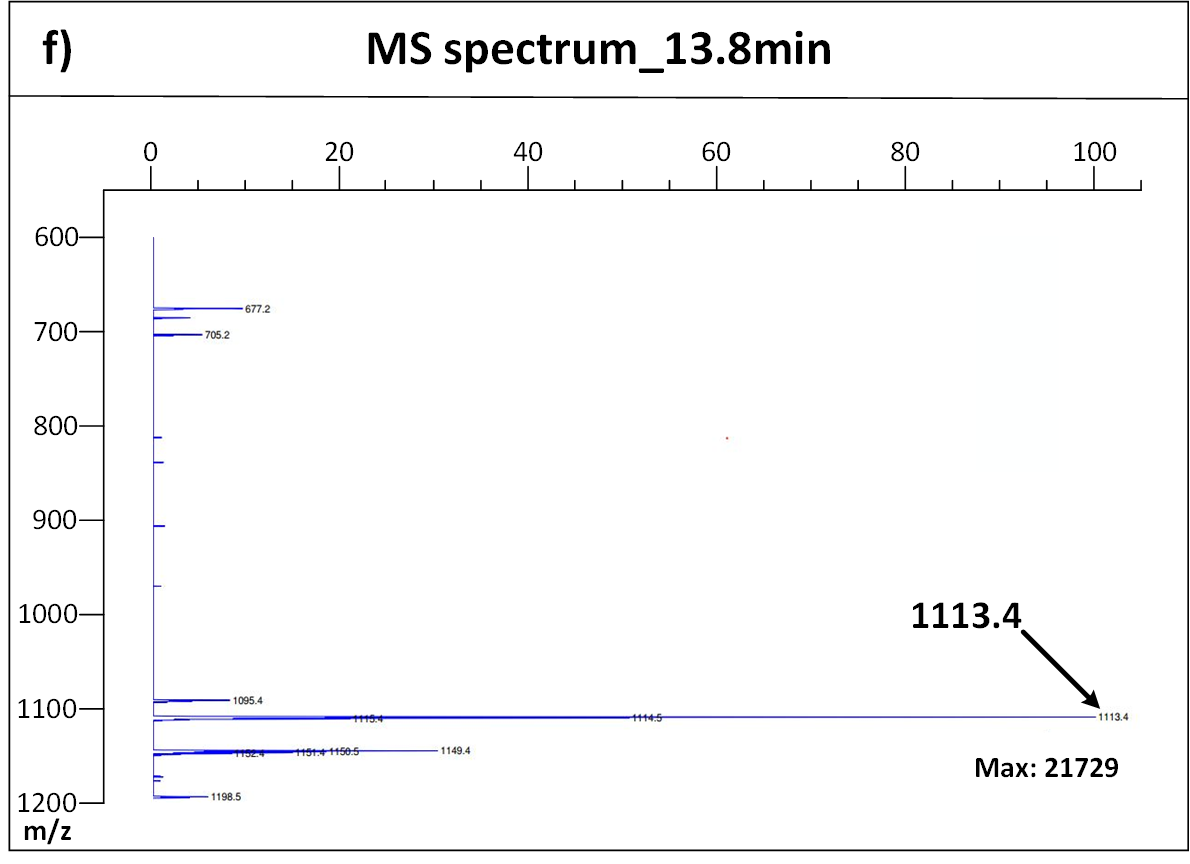

Supplement: Supplementary file 3 — Supplementary Material 3. Figure S3. LC–MS spectra of sophorolipid products obtained after incubation of bola SLs with rSBLE. [file 13068_2024_2533_MOESM3_ESM.zip › Fig. S3/Fig S3 f).png]

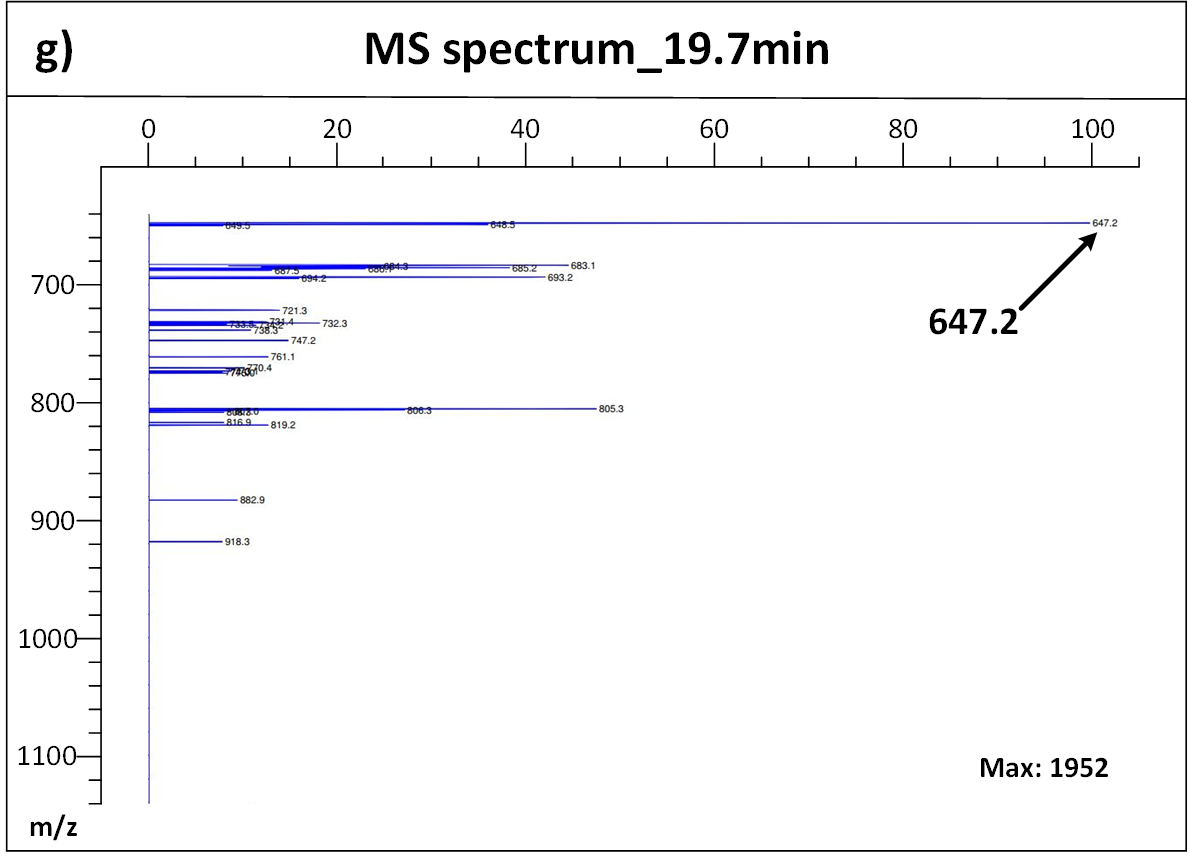

Supplement: Supplementary file 3 — Supplementary Material 3. Figure S3. LC–MS spectra of sophorolipid products obtained after incubation of bola SLs with rSBLE. [file 13068_2024_2533_MOESM3_ESM.zip › Fig. S3/Fig S3 g).png]

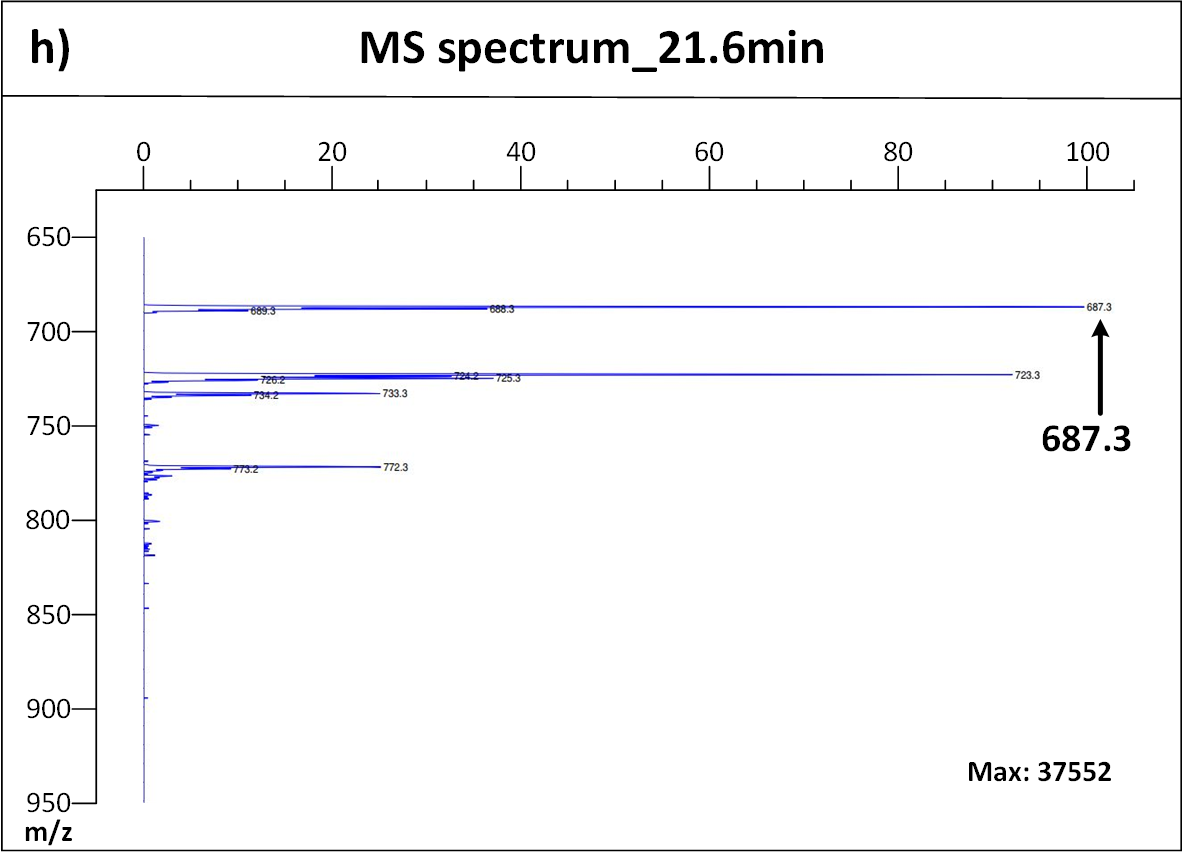

Supplement: Supplementary file 3 — Supplementary Material 3. Figure S3. LC–MS spectra of sophorolipid products obtained after incubation of bola SLs with rSBLE. [file 13068_2024_2533_MOESM3_ESM.zip › Fig. S3/Fig S3 h).png]

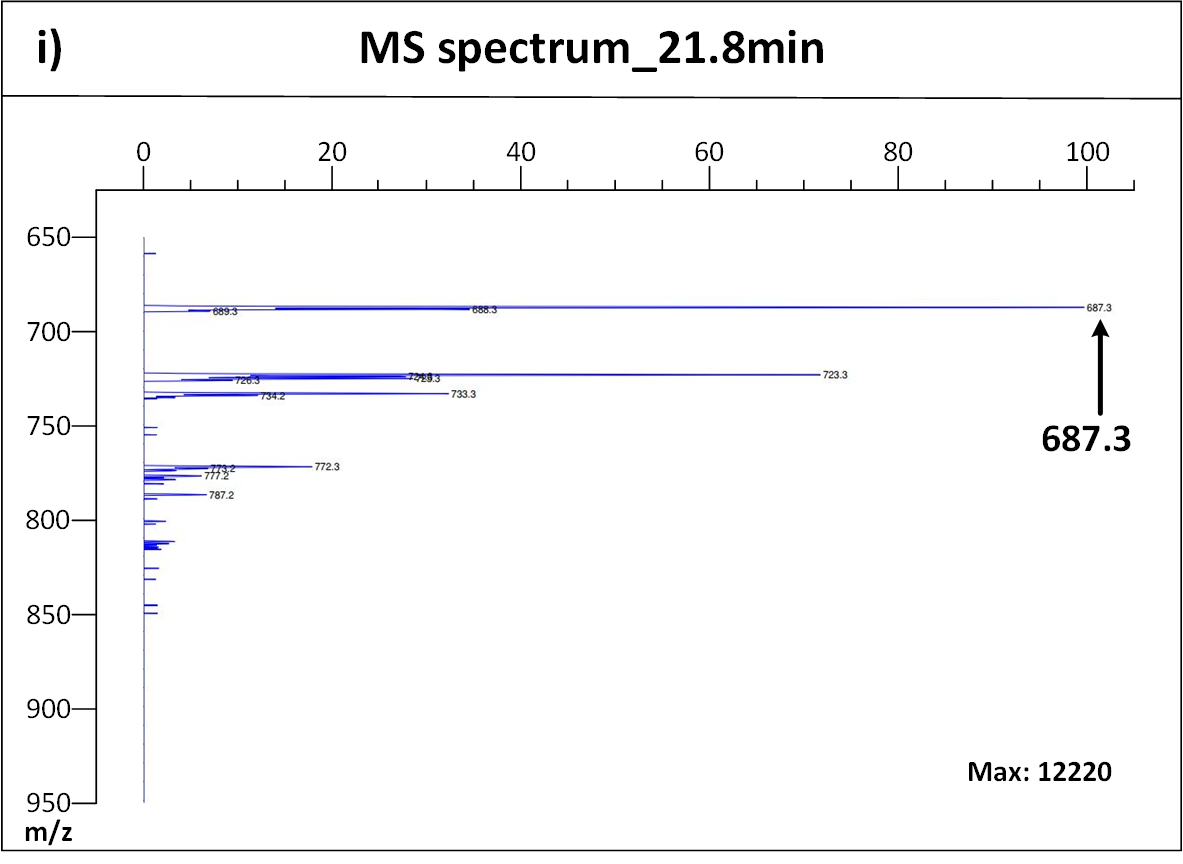

Supplement: Supplementary file 3 — Supplementary Material 3. Figure S3. LC–MS spectra of sophorolipid products obtained after incubation of bola SLs with rSBLE. [file 13068_2024_2533_MOESM3_ESM.zip › Fig. S3/Fig S3 i).png]

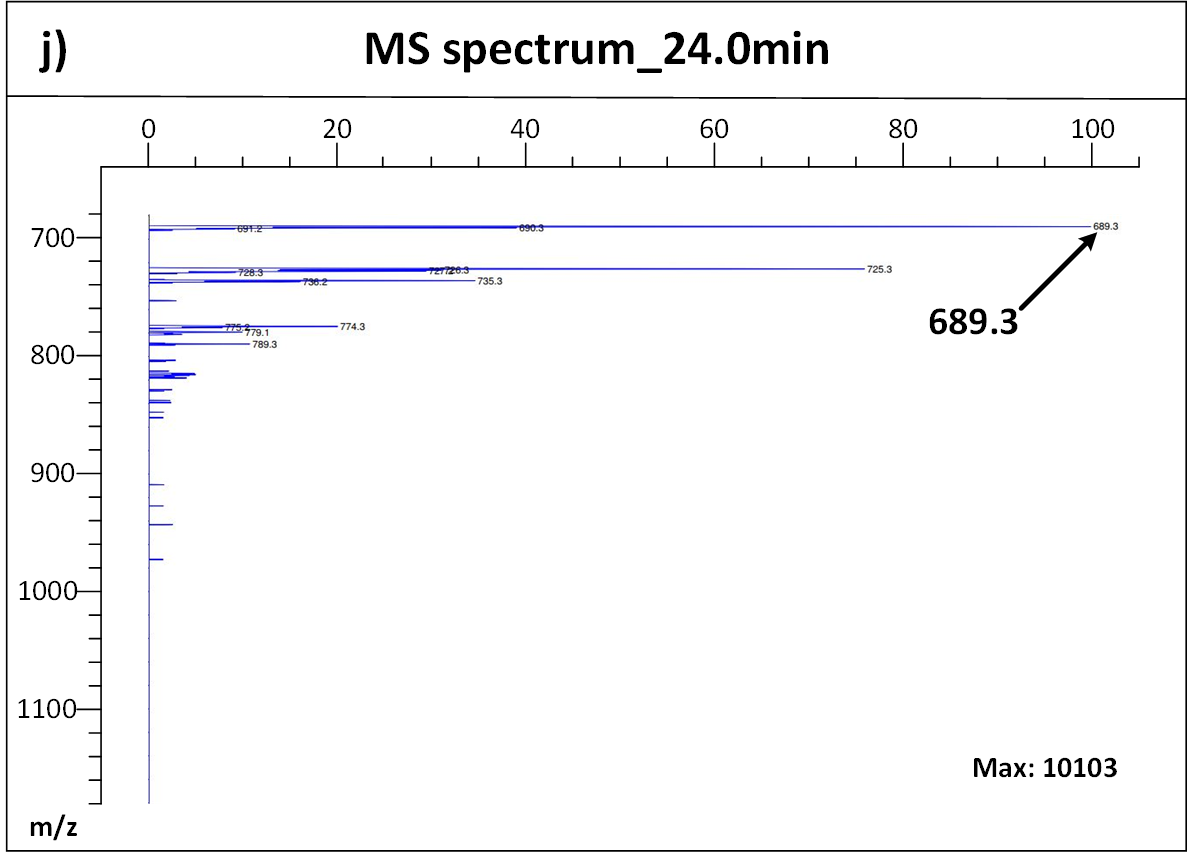

Supplement: Supplementary file 3 — Supplementary Material 3. Figure S3. LC–MS spectra of sophorolipid products obtained after incubation of bola SLs with rSBLE. [file 13068_2024_2533_MOESM3_ESM.zip › Fig. S3/Fig S3 j).png]

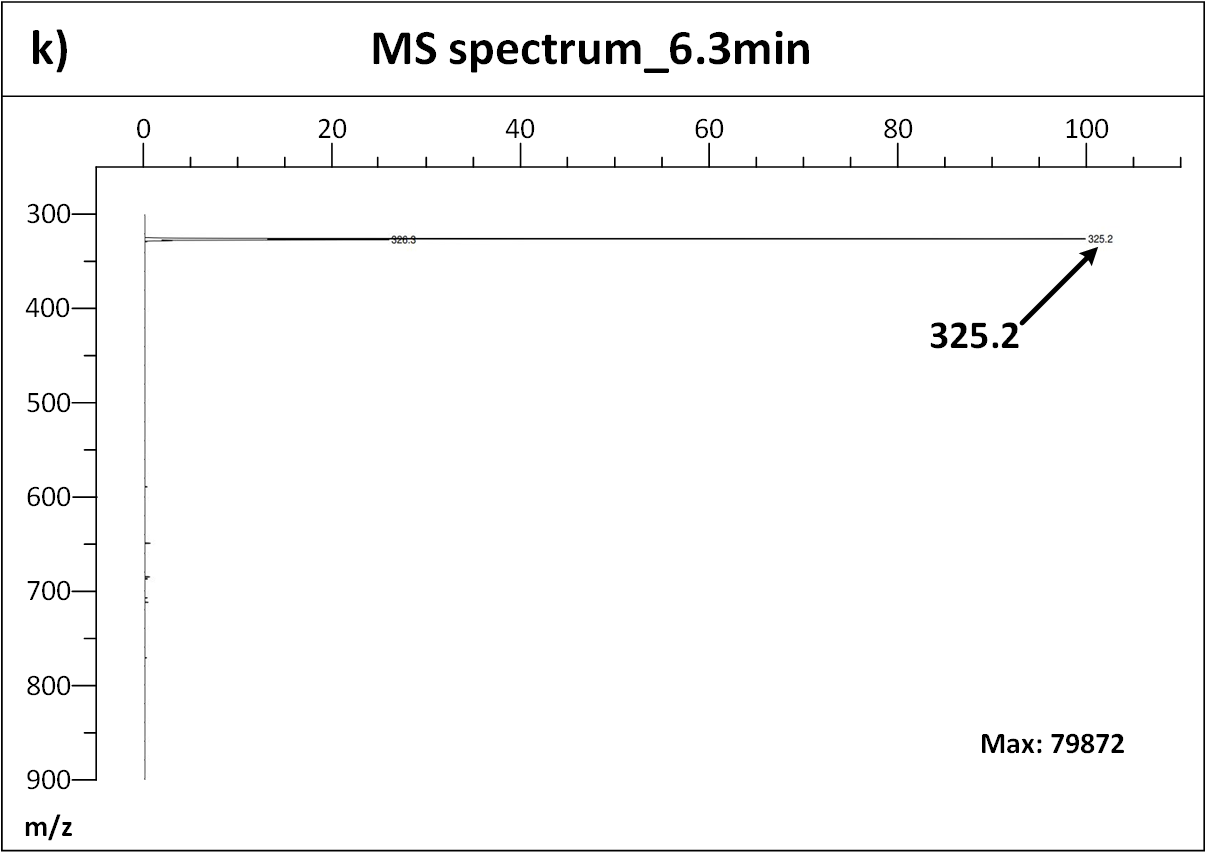

Supplement: Supplementary file 3 — Supplementary Material 3. Figure S3. LC–MS spectra of sophorolipid products obtained after incubation of bola SLs with rSBLE. [file 13068_2024_2533_MOESM3_ESM.zip › Fig. S3/Fig S3 k).png]

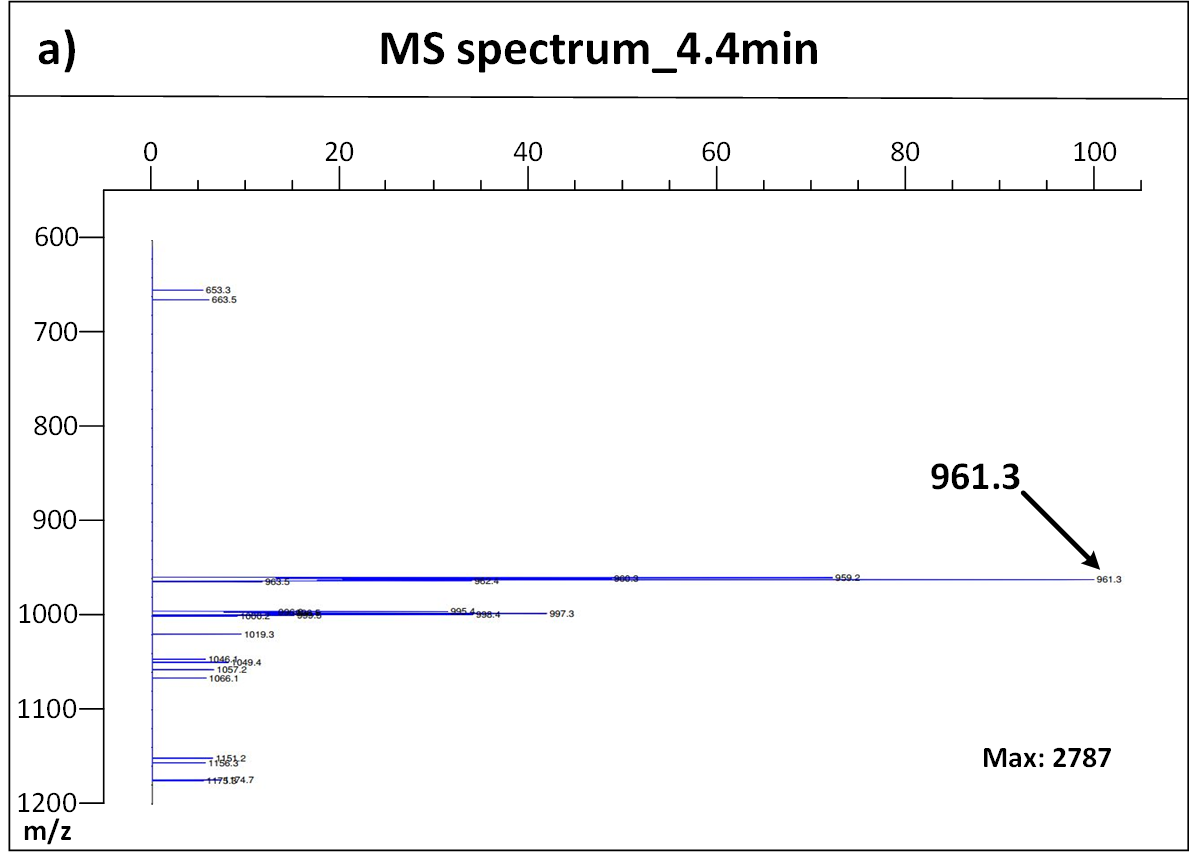

Supplement: Supplementary file 4 — Supplementary Material 4. Figure S4. MS/MS spectra of bola SLs and formed lactonic SLs after enzymatic conversion with rSBLE. [file 13068_2024_2533_MOESM4_ESM.zip › Fig. S4/Fig S4 a).png]

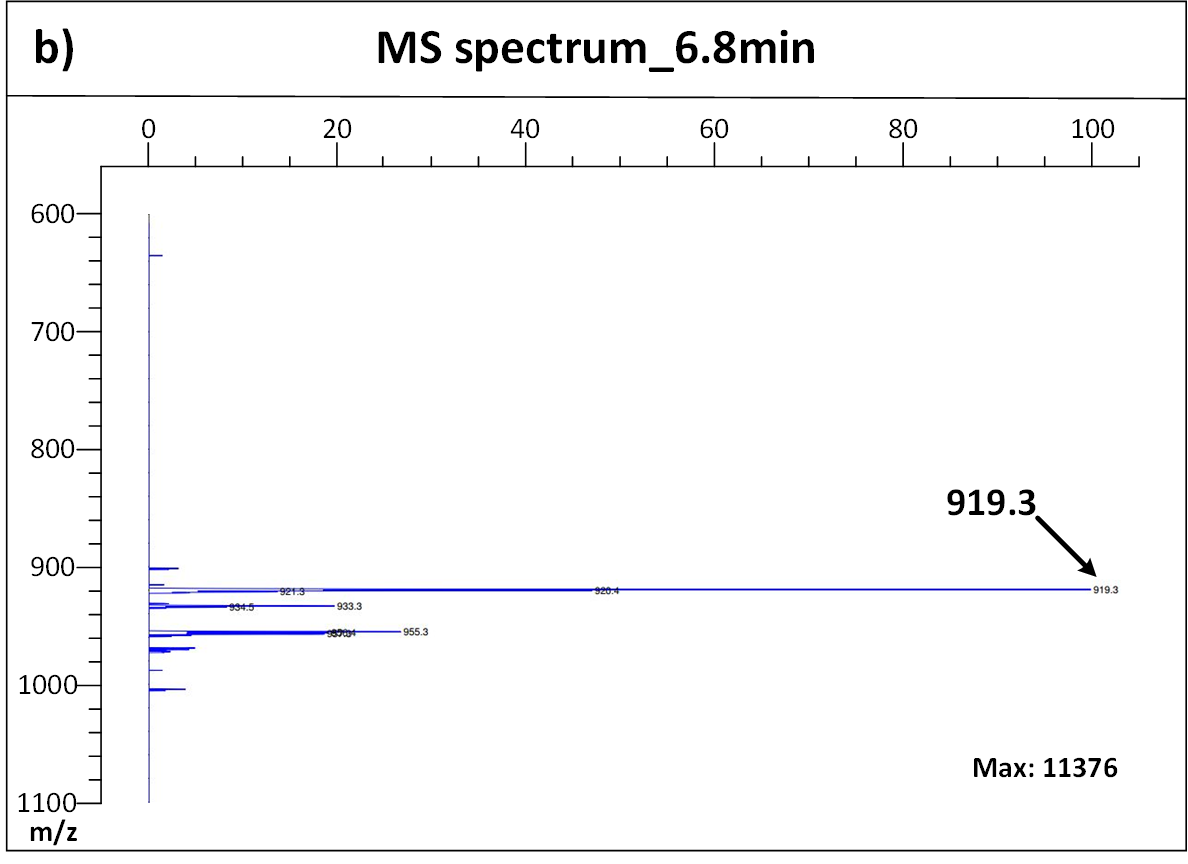

Supplement: Supplementary file 4 — Supplementary Material 4. Figure S4. MS/MS spectra of bola SLs and formed lactonic SLs after enzymatic conversion with rSBLE. [file 13068_2024_2533_MOESM4_ESM.zip › Fig. S4/Fig S4 b).png]

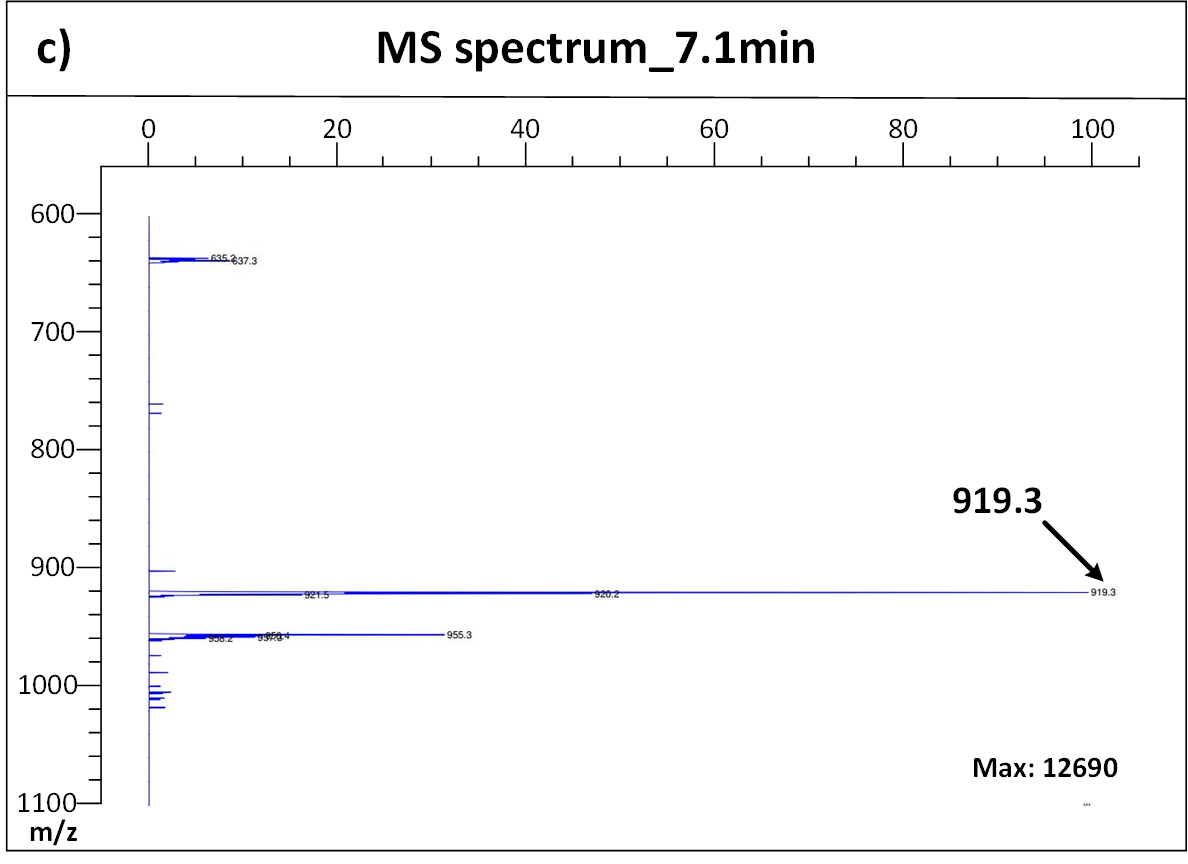

Supplement: Supplementary file 4 — Supplementary Material 4. Figure S4. MS/MS spectra of bola SLs and formed lactonic SLs after enzymatic conversion with rSBLE. [file 13068_2024_2533_MOESM4_ESM.zip › Fig. S4/Fig S4 c).png]

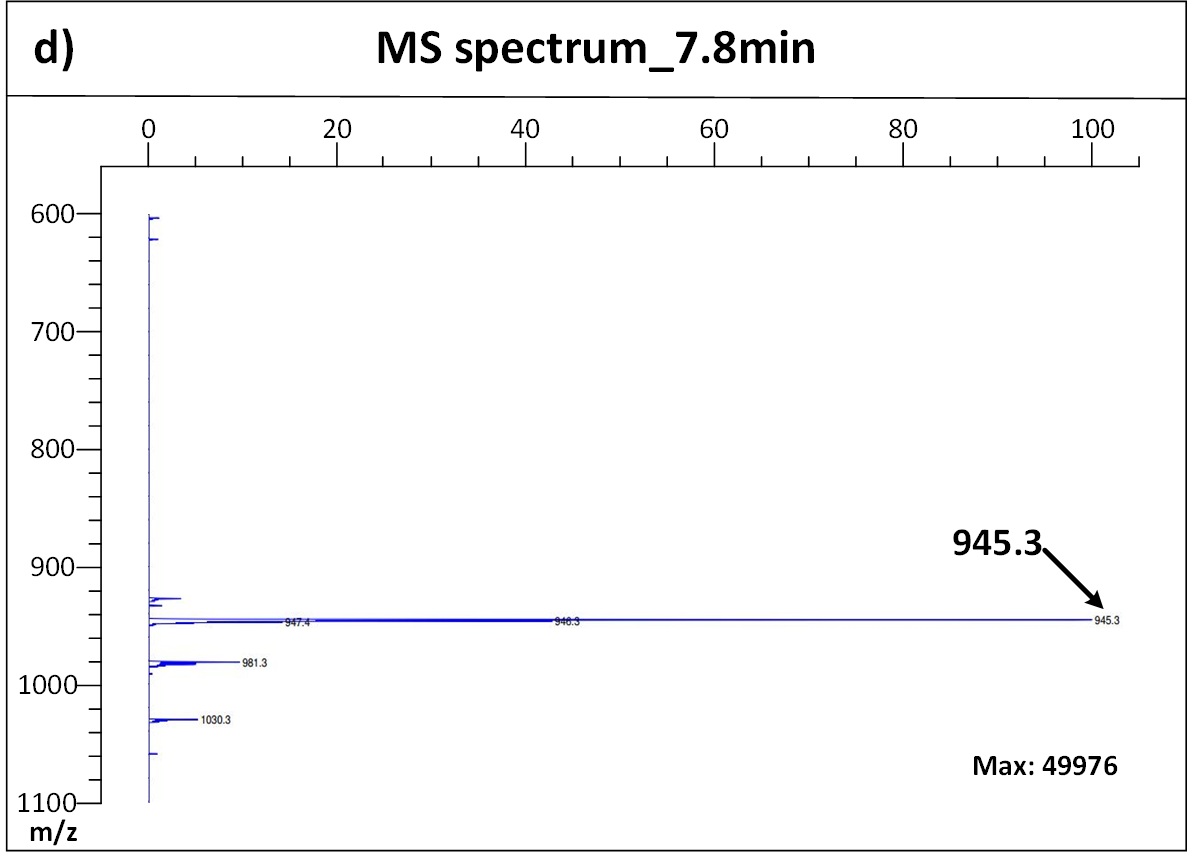

Supplement: Supplementary file 4 — Supplementary Material 4. Figure S4. MS/MS spectra of bola SLs and formed lactonic SLs after enzymatic conversion with rSBLE. [file 13068_2024_2533_MOESM4_ESM.zip › Fig. S4/Fig S4 d).png]

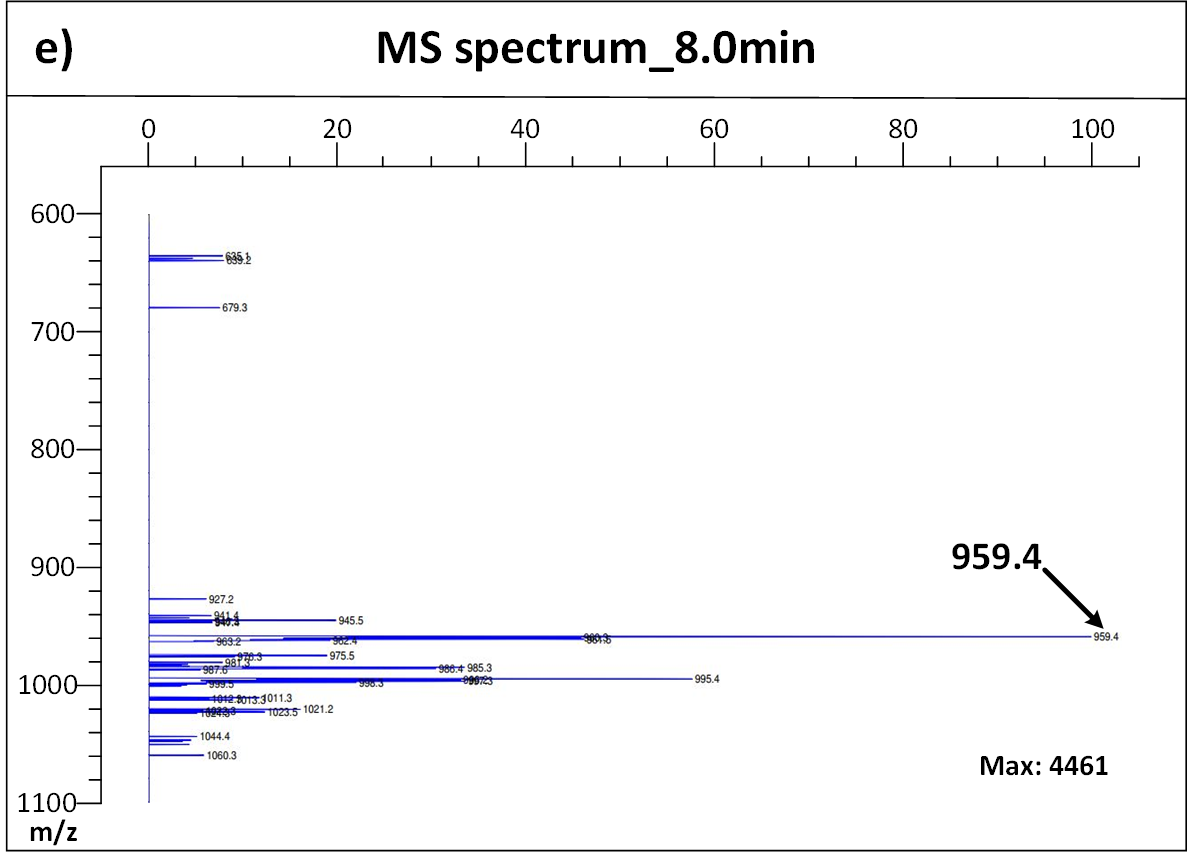

Supplement: Supplementary file 4 — Supplementary Material 4. Figure S4. MS/MS spectra of bola SLs and formed lactonic SLs after enzymatic conversion with rSBLE. [file 13068_2024_2533_MOESM4_ESM.zip › Fig. S4/Fig S4 e).png]

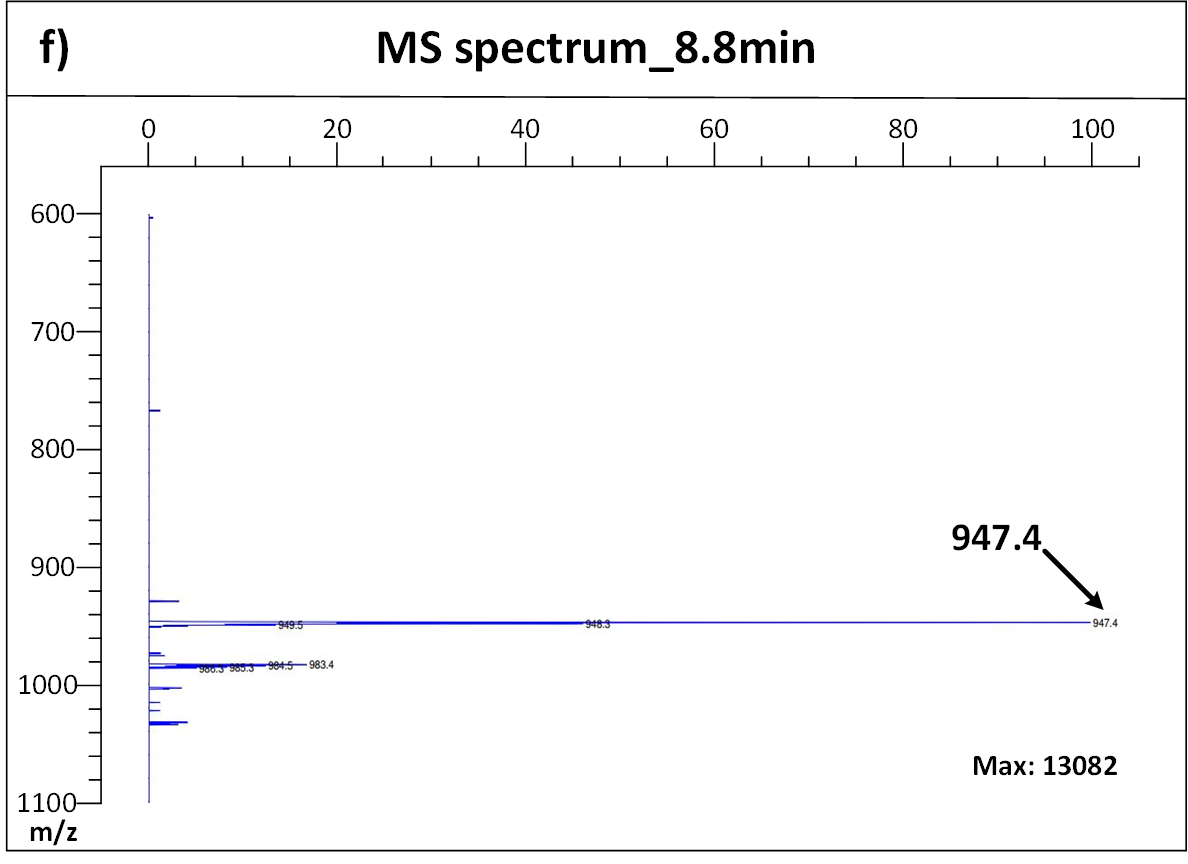

Supplement: Supplementary file 4 — Supplementary Material 4. Figure S4. MS/MS spectra of bola SLs and formed lactonic SLs after enzymatic conversion with rSBLE. [file 13068_2024_2533_MOESM4_ESM.zip › Fig. S4/Fig S4 f).png]

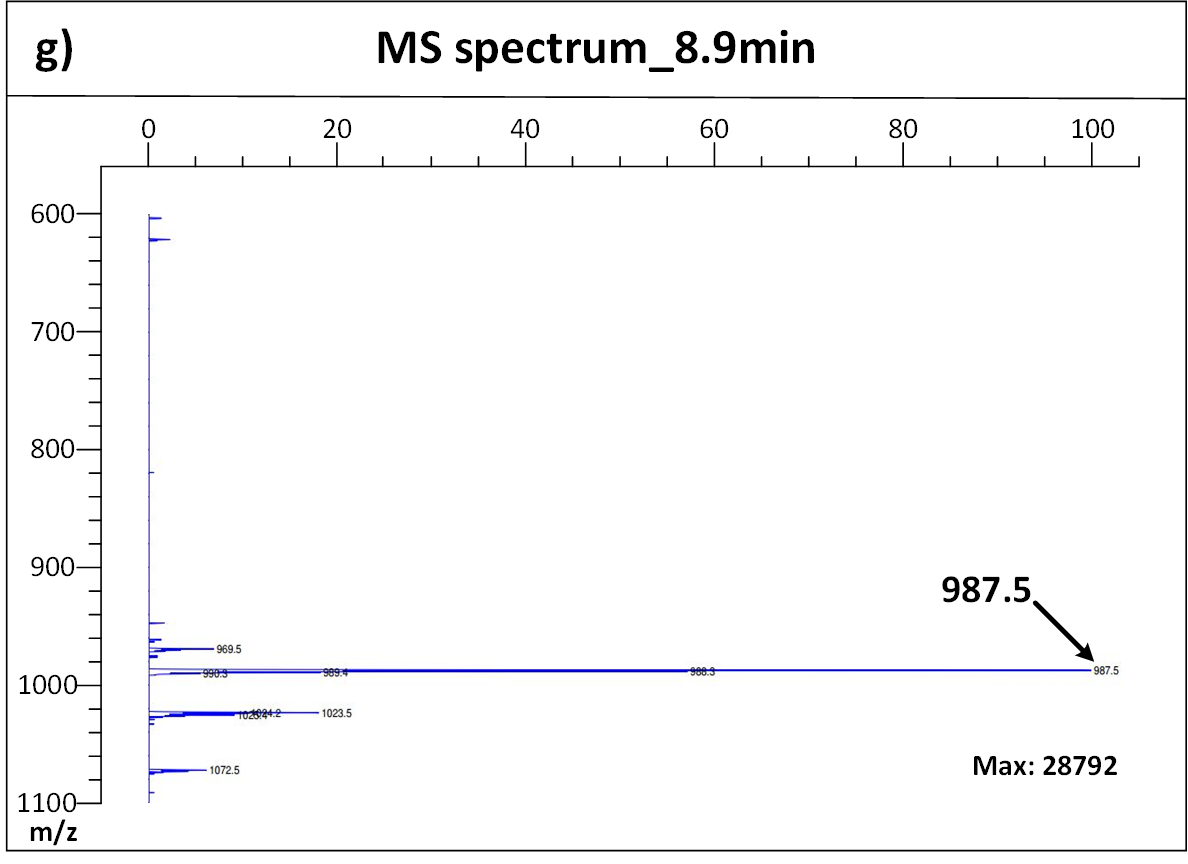

Supplement: Supplementary file 4 — Supplementary Material 4. Figure S4. MS/MS spectra of bola SLs and formed lactonic SLs after enzymatic conversion with rSBLE. [file 13068_2024_2533_MOESM4_ESM.zip › Fig. S4/Fig S4 g).png]

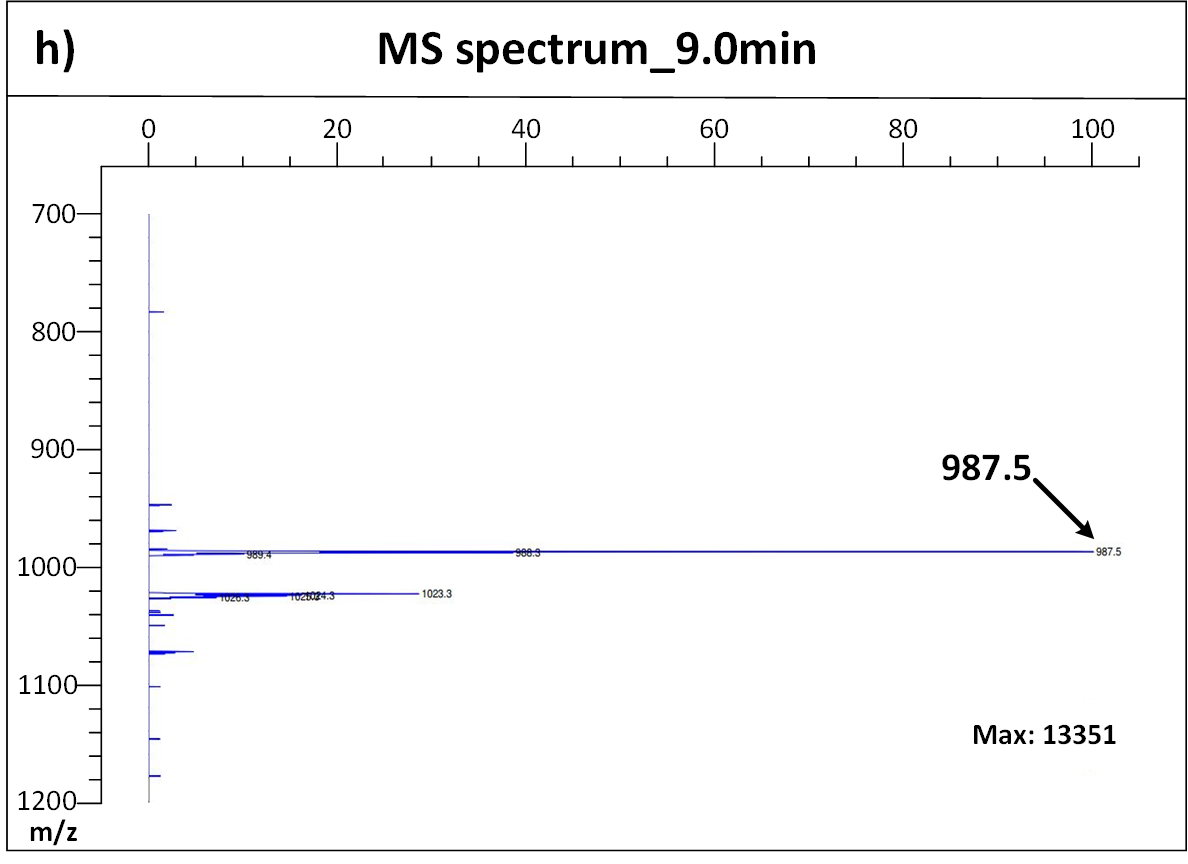

Supplement: Supplementary file 4 — Supplementary Material 4. Figure S4. MS/MS spectra of bola SLs and formed lactonic SLs after enzymatic conversion with rSBLE. [file 13068_2024_2533_MOESM4_ESM.zip › Fig. S4/Fig S4 h).png]

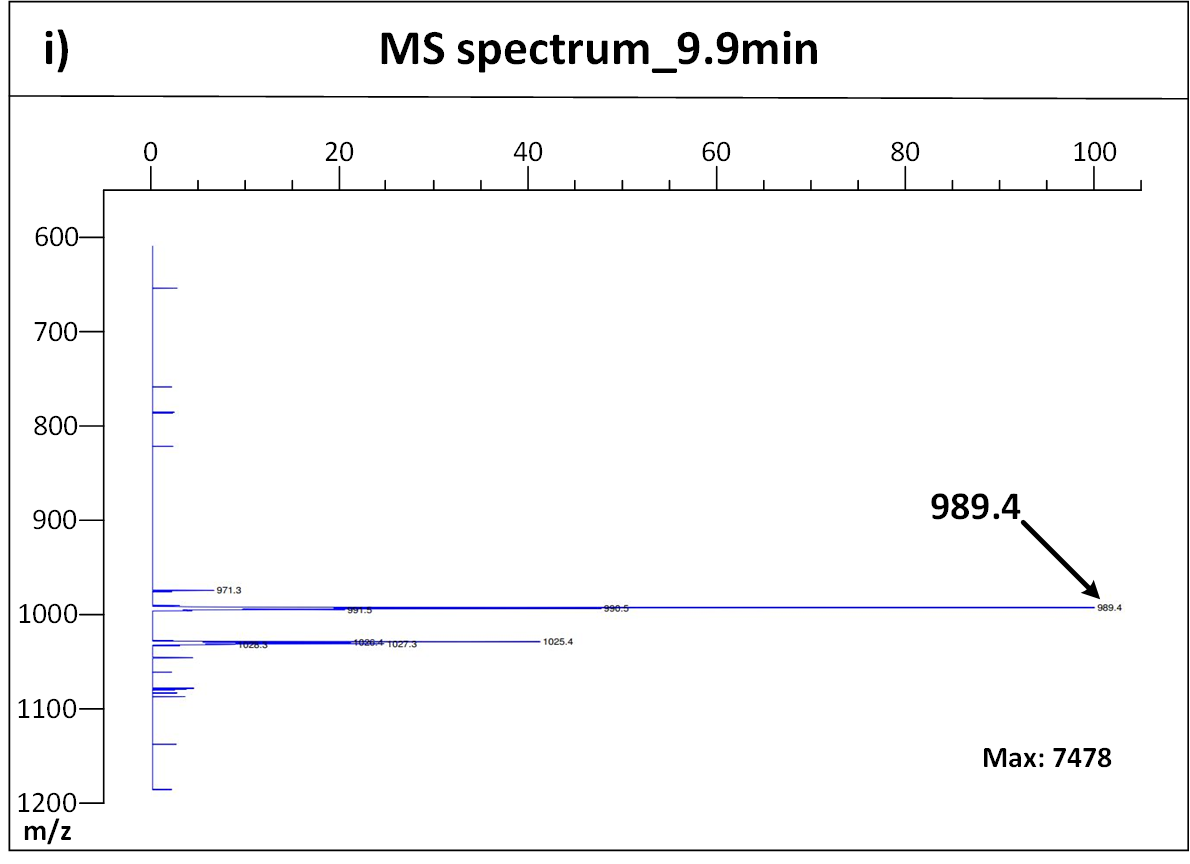

Supplement: Supplementary file 4 — Supplementary Material 4. Figure S4. MS/MS spectra of bola SLs and formed lactonic SLs after enzymatic conversion with rSBLE. [file 13068_2024_2533_MOESM4_ESM.zip › Fig. S4/Fig S4 i).png]

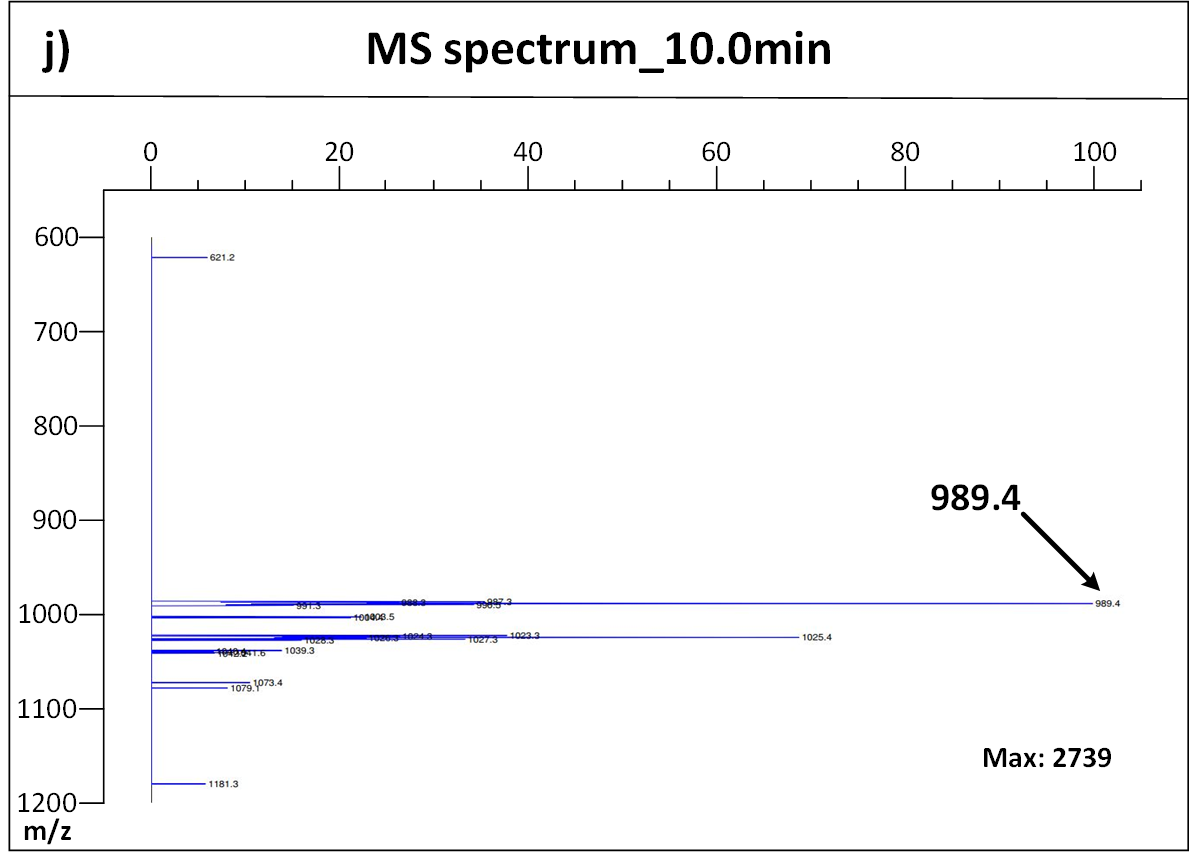

Supplement: Supplementary file 4 — Supplementary Material 4. Figure S4. MS/MS spectra of bola SLs and formed lactonic SLs after enzymatic conversion with rSBLE. [file 13068_2024_2533_MOESM4_ESM.zip › Fig. S4/Fig S4 j).png]

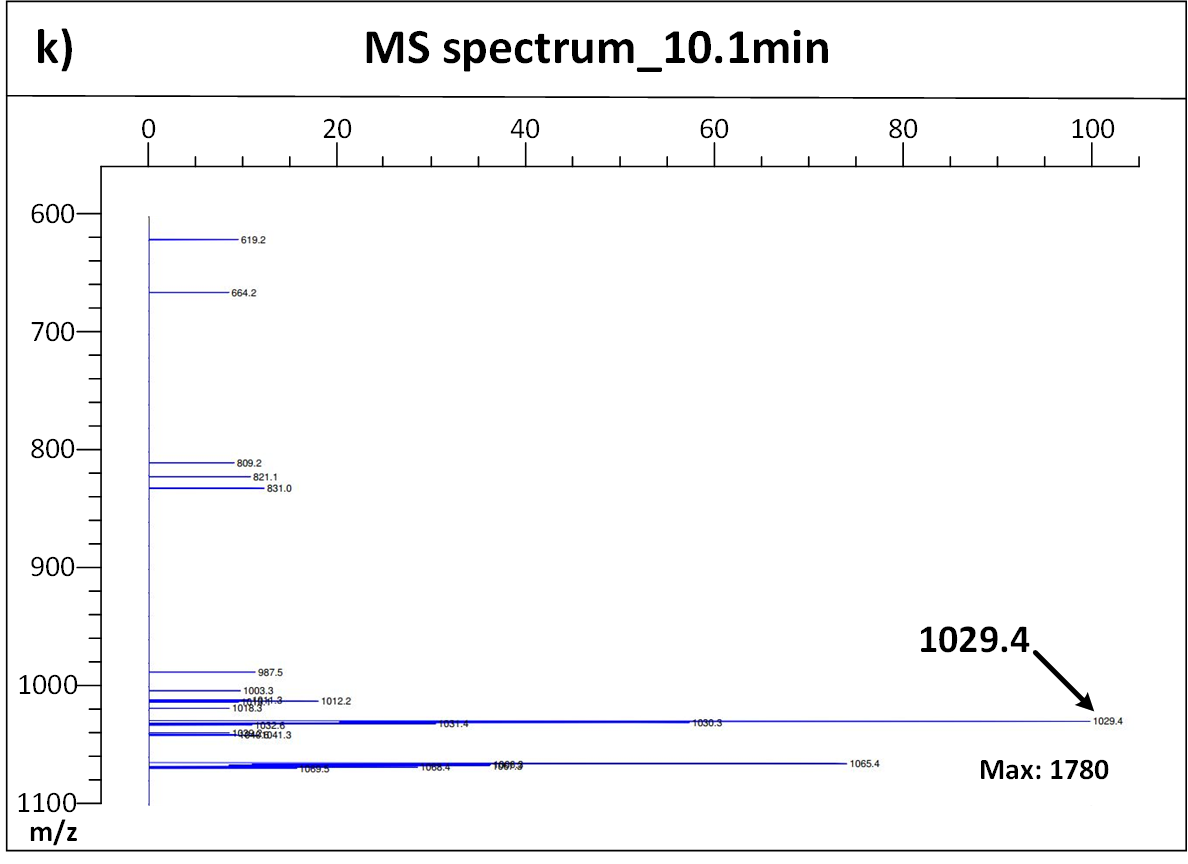

Supplement: Supplementary file 4 — Supplementary Material 4. Figure S4. MS/MS spectra of bola SLs and formed lactonic SLs after enzymatic conversion with rSBLE. [file 13068_2024_2533_MOESM4_ESM.zip › Fig. S4/Fig S4 k).png]

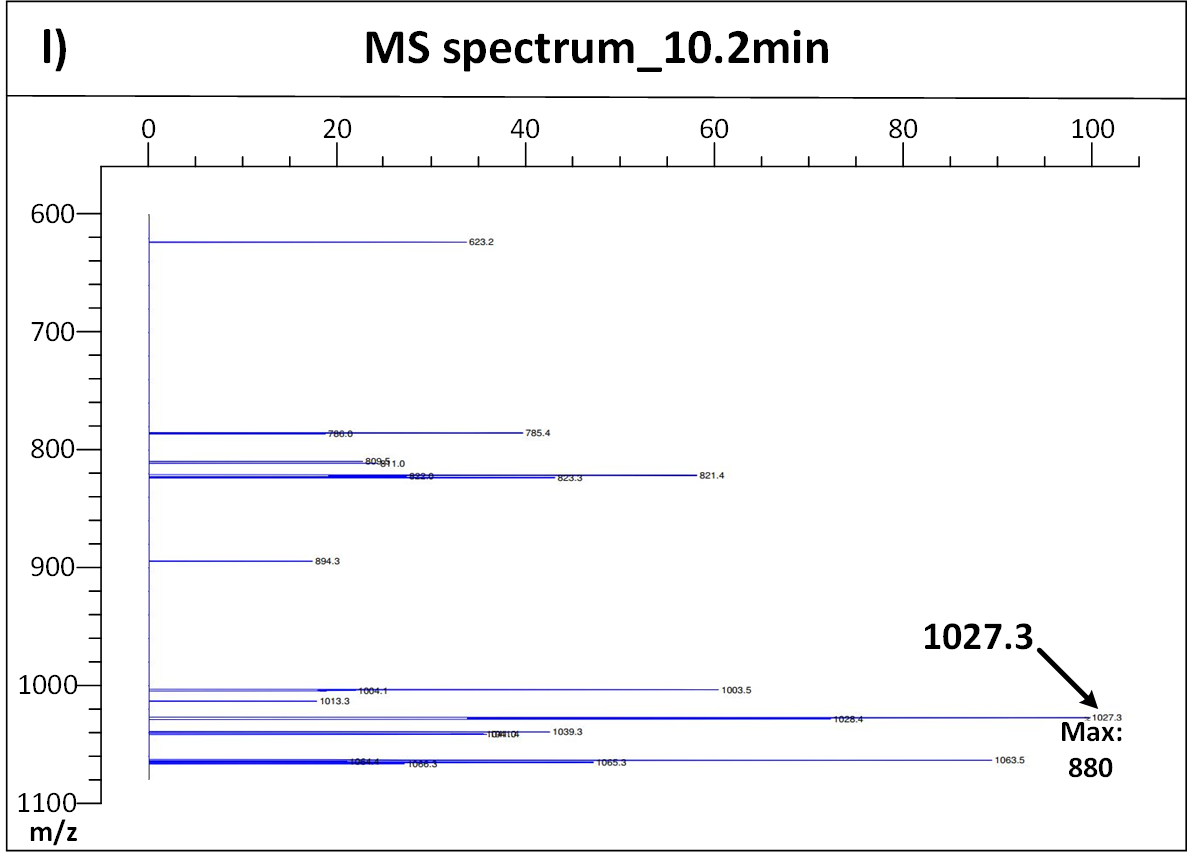

Supplement: Supplementary file 4 — Supplementary Material 4. Figure S4. MS/MS spectra of bola SLs and formed lactonic SLs after enzymatic conversion with rSBLE. [file 13068_2024_2533_MOESM4_ESM.zip › Fig. S4/Fig S4 l).png]

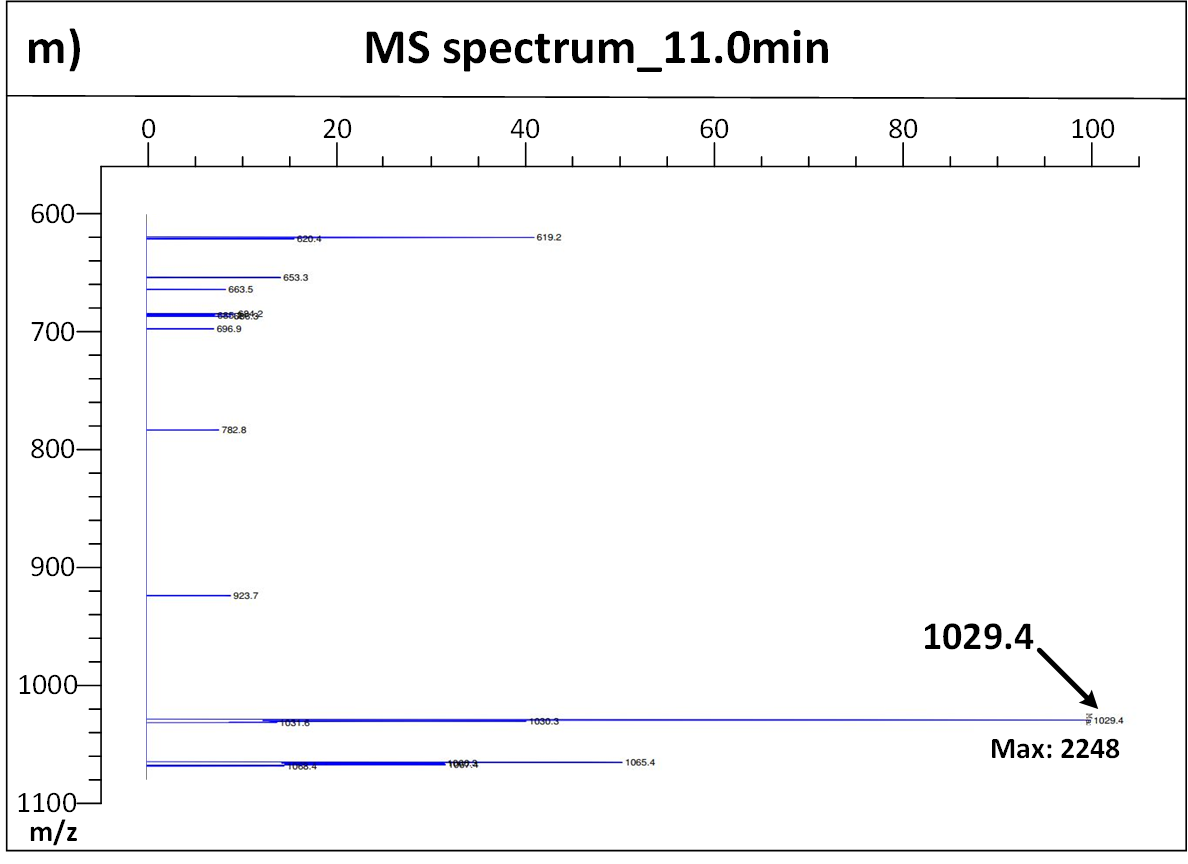

Supplement: Supplementary file 4 — Supplementary Material 4. Figure S4. MS/MS spectra of bola SLs and formed lactonic SLs after enzymatic conversion with rSBLE. [file 13068_2024_2533_MOESM4_ESM.zip › Fig. S4/Fig S4 m).png]

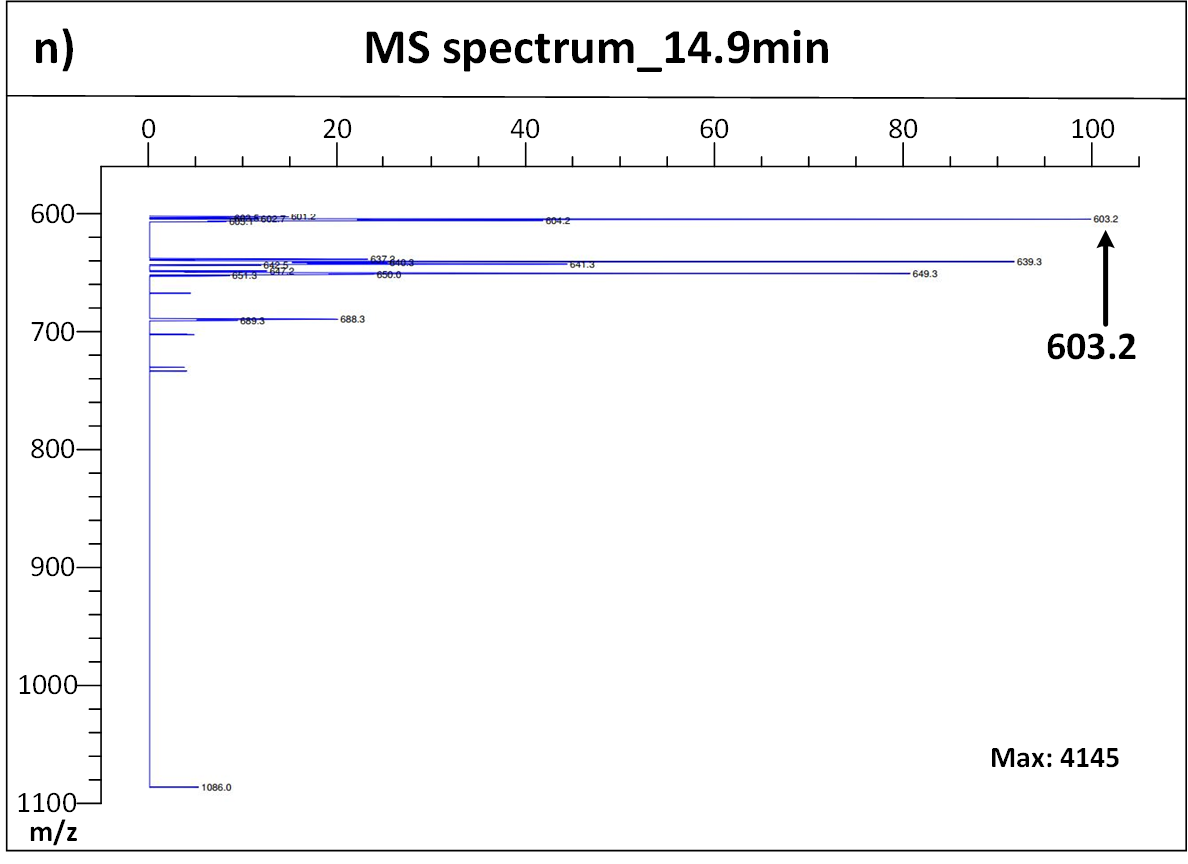

Supplement: Supplementary file 4 — Supplementary Material 4. Figure S4. MS/MS spectra of bola SLs and formed lactonic SLs after enzymatic conversion with rSBLE. [file 13068_2024_2533_MOESM4_ESM.zip › Fig. S4/Fig S4 n).png]

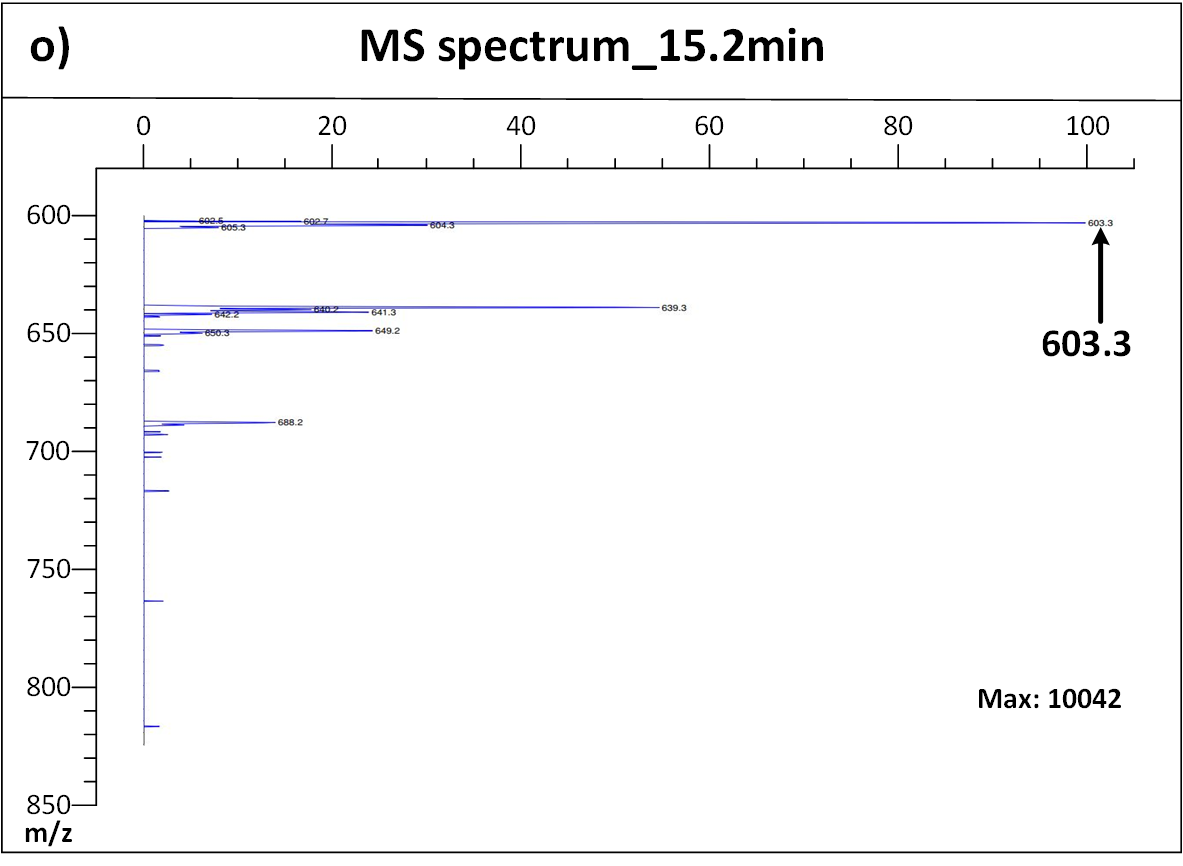

Supplement: Supplementary file 4 — Supplementary Material 4. Figure S4. MS/MS spectra of bola SLs and formed lactonic SLs after enzymatic conversion with rSBLE. [file 13068_2024_2533_MOESM4_ESM.zip › Fig. S4/Fig S4 o).png]

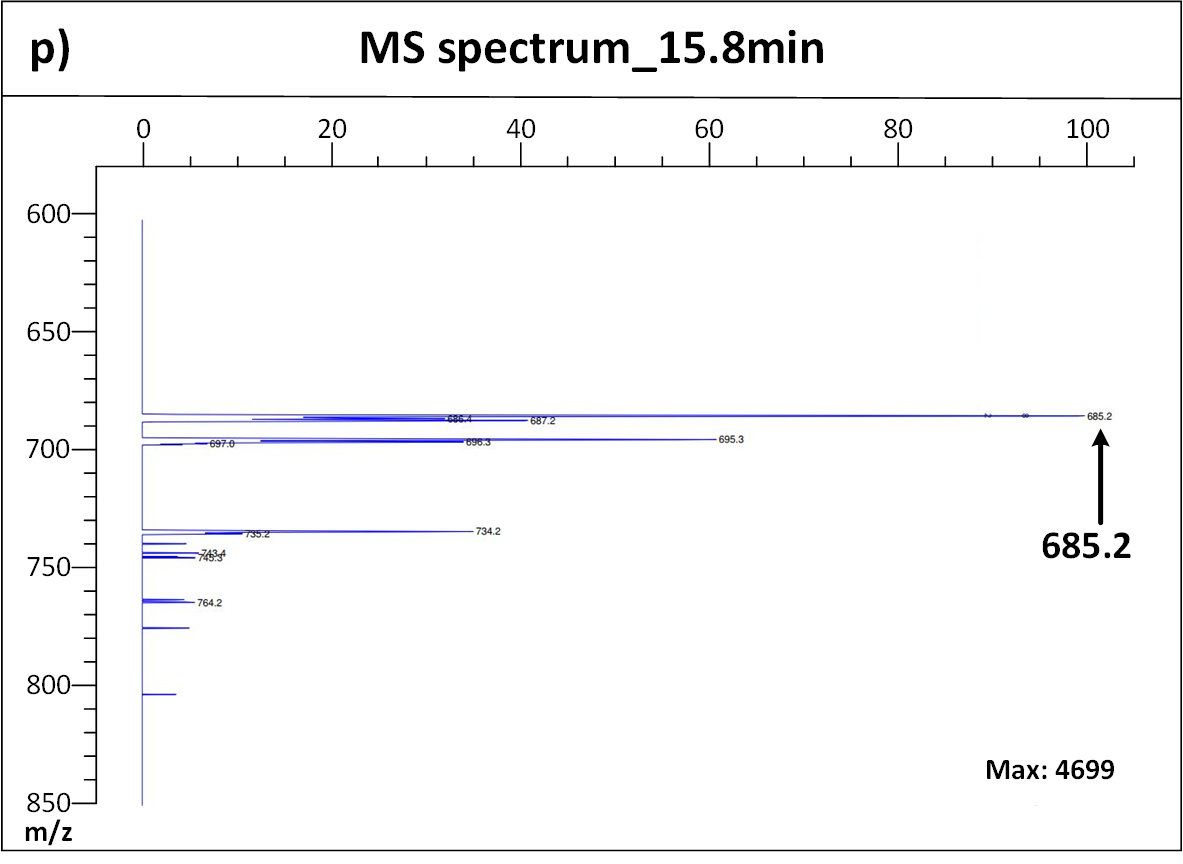

Supplement: Supplementary file 4 — Supplementary Material 4. Figure S4. MS/MS spectra of bola SLs and formed lactonic SLs after enzymatic conversion with rSBLE. [file 13068_2024_2533_MOESM4_ESM.zip › Fig. S4/Fig S4 p).png]

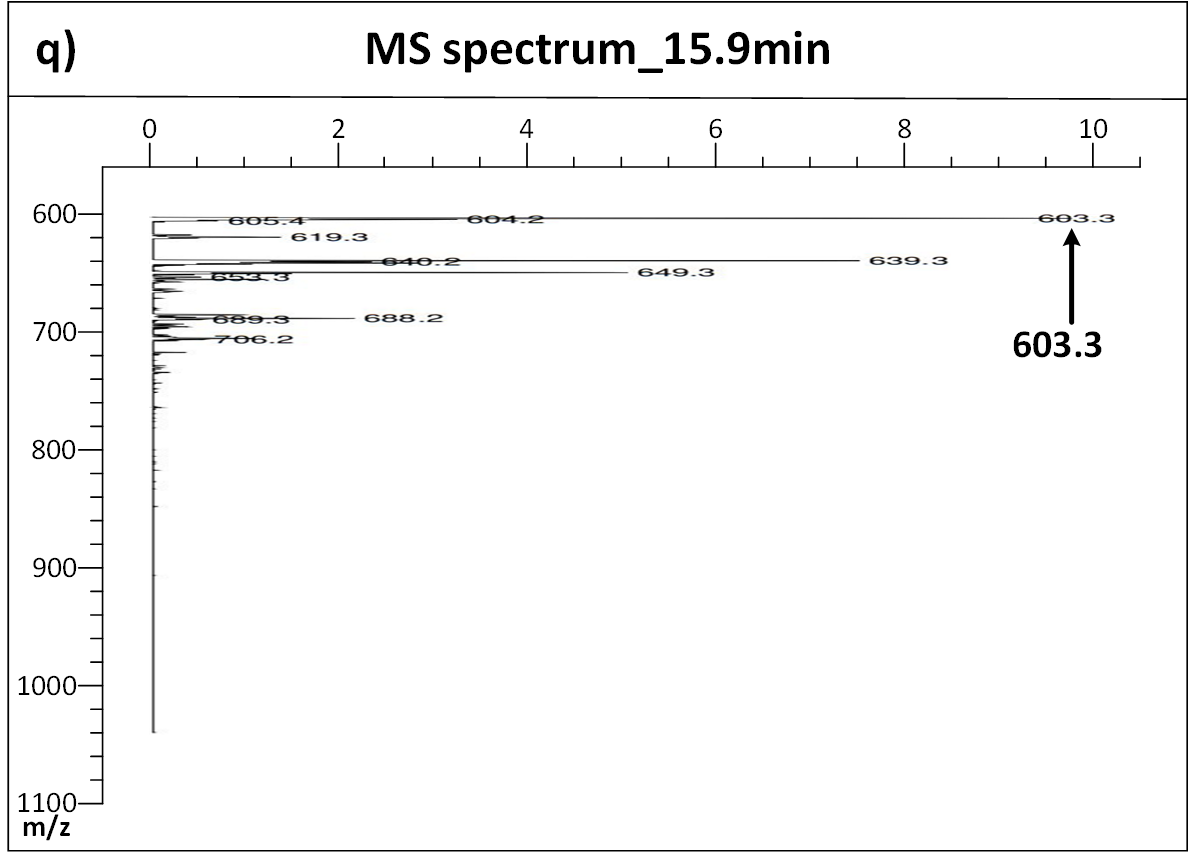

Supplement: Supplementary file 4 — Supplementary Material 4. Figure S4. MS/MS spectra of bola SLs and formed lactonic SLs after enzymatic conversion with rSBLE. [file 13068_2024_2533_MOESM4_ESM.zip › Fig. S4/Fig S4 q).png]

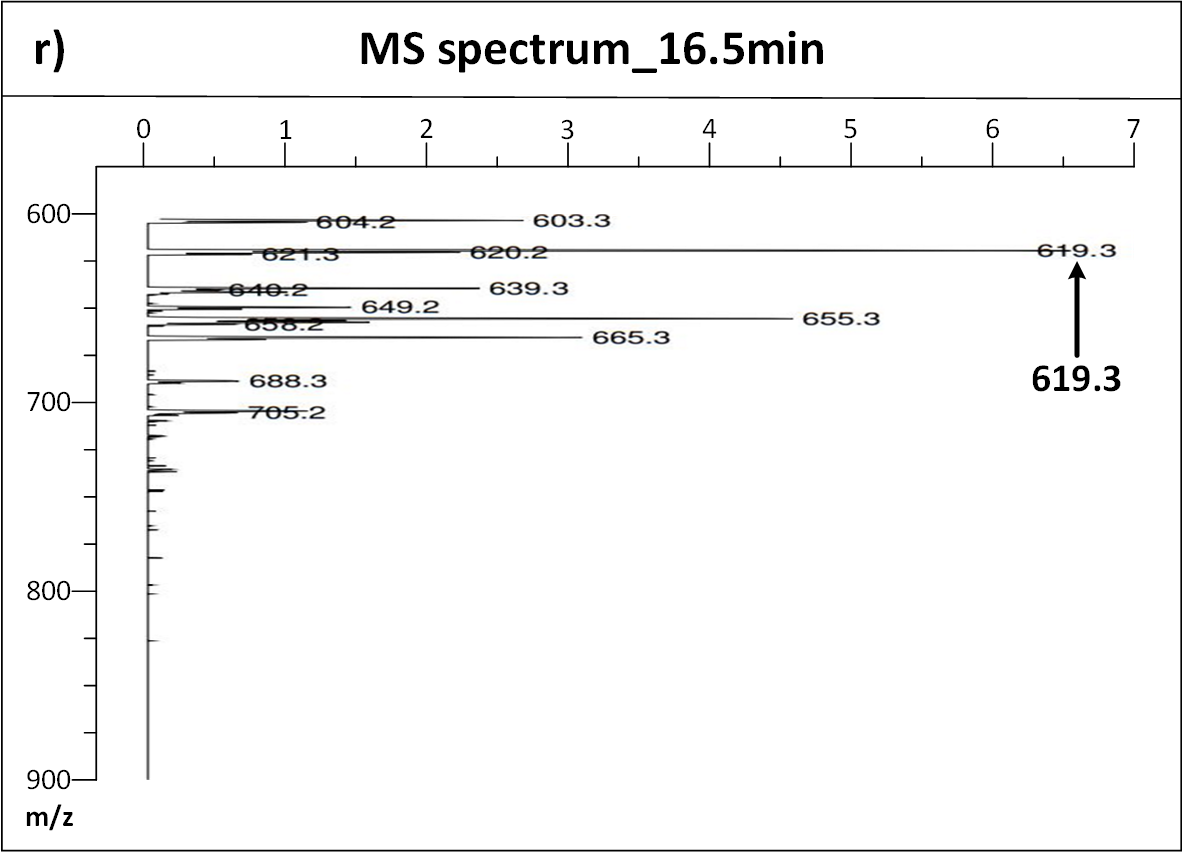

Supplement: Supplementary file 4 — Supplementary Material 4. Figure S4. MS/MS spectra of bola SLs and formed lactonic SLs after enzymatic conversion with rSBLE. [file 13068_2024_2533_MOESM4_ESM.zip › Fig. S4/Fig S4 r).png]

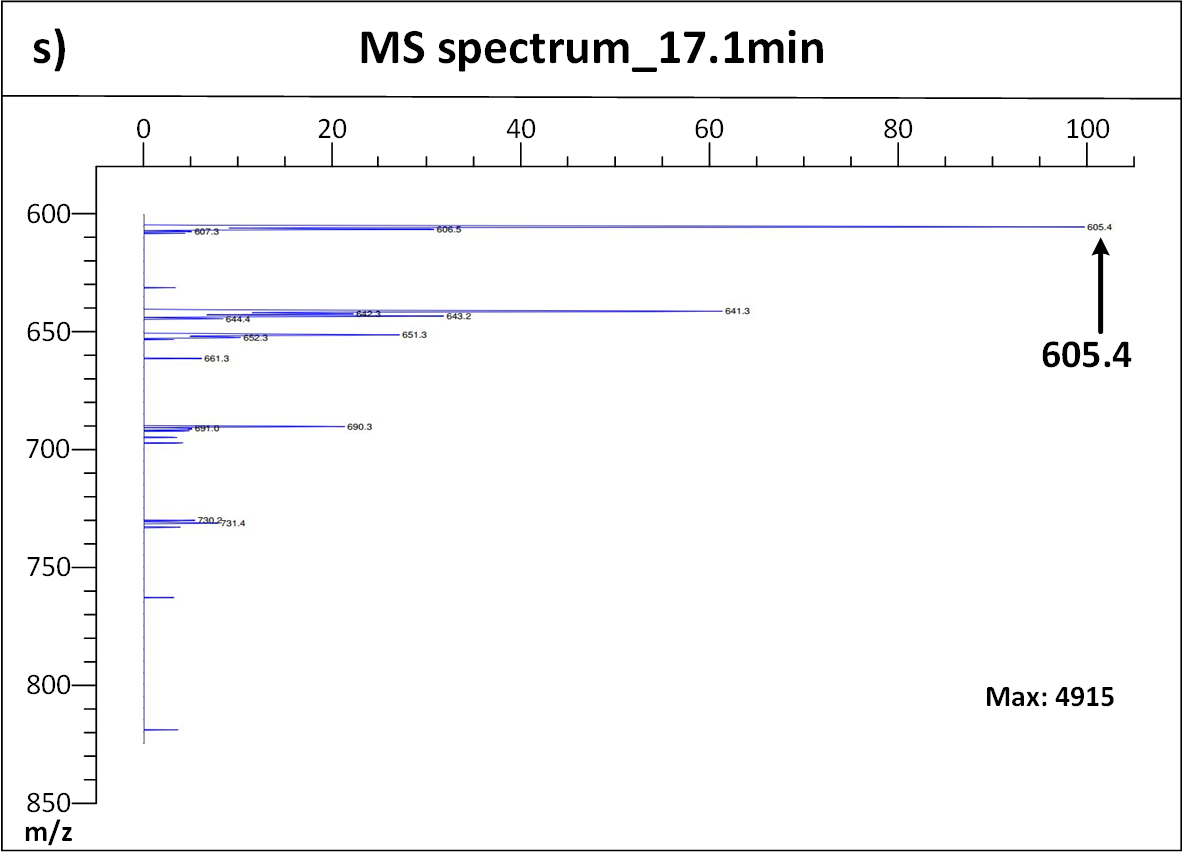

Supplement: Supplementary file 4 — Supplementary Material 4. Figure S4. MS/MS spectra of bola SLs and formed lactonic SLs after enzymatic conversion with rSBLE. [file 13068_2024_2533_MOESM4_ESM.zip › Fig. S4/Fig S4 s).png]

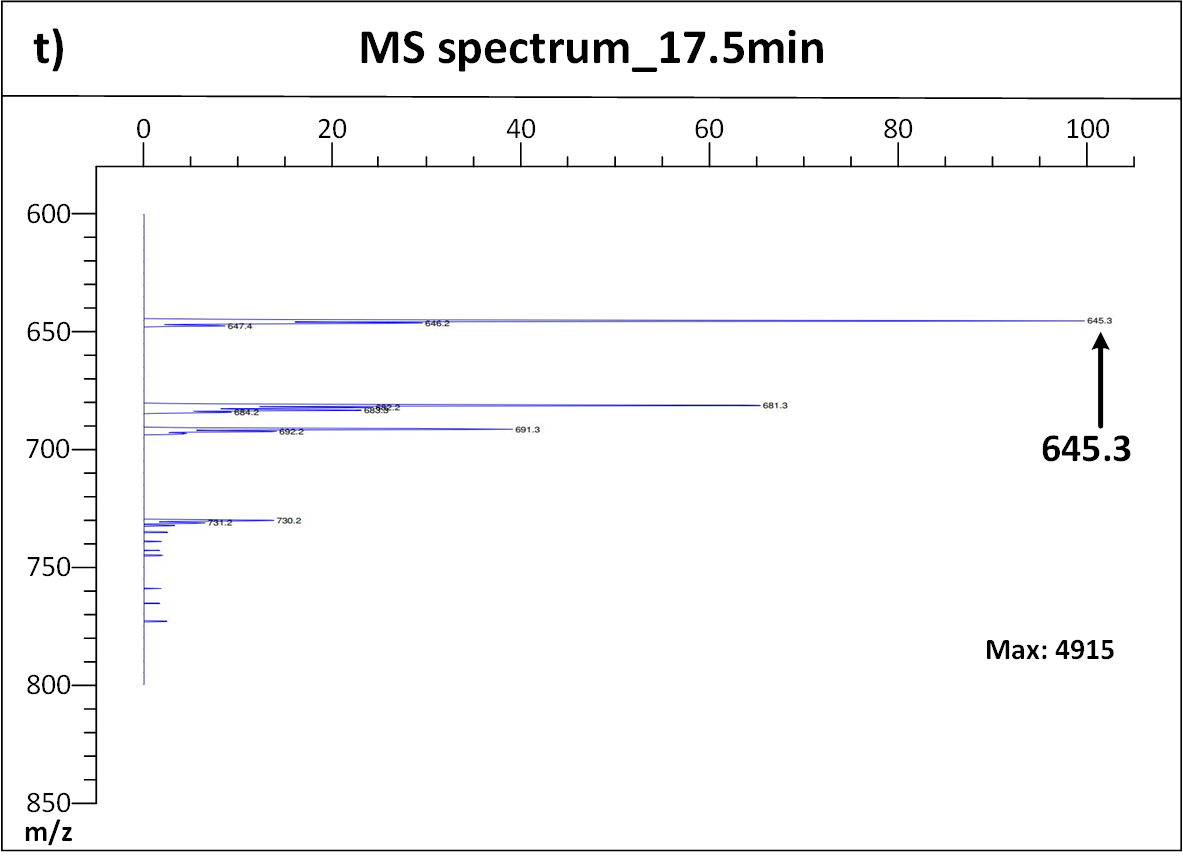

Supplement: Supplementary file 4 — Supplementary Material 4. Figure S4. MS/MS spectra of bola SLs and formed lactonic SLs after enzymatic conversion with rSBLE. [file 13068_2024_2533_MOESM4_ESM.zip › Fig. S4/Fig S4 t).png]

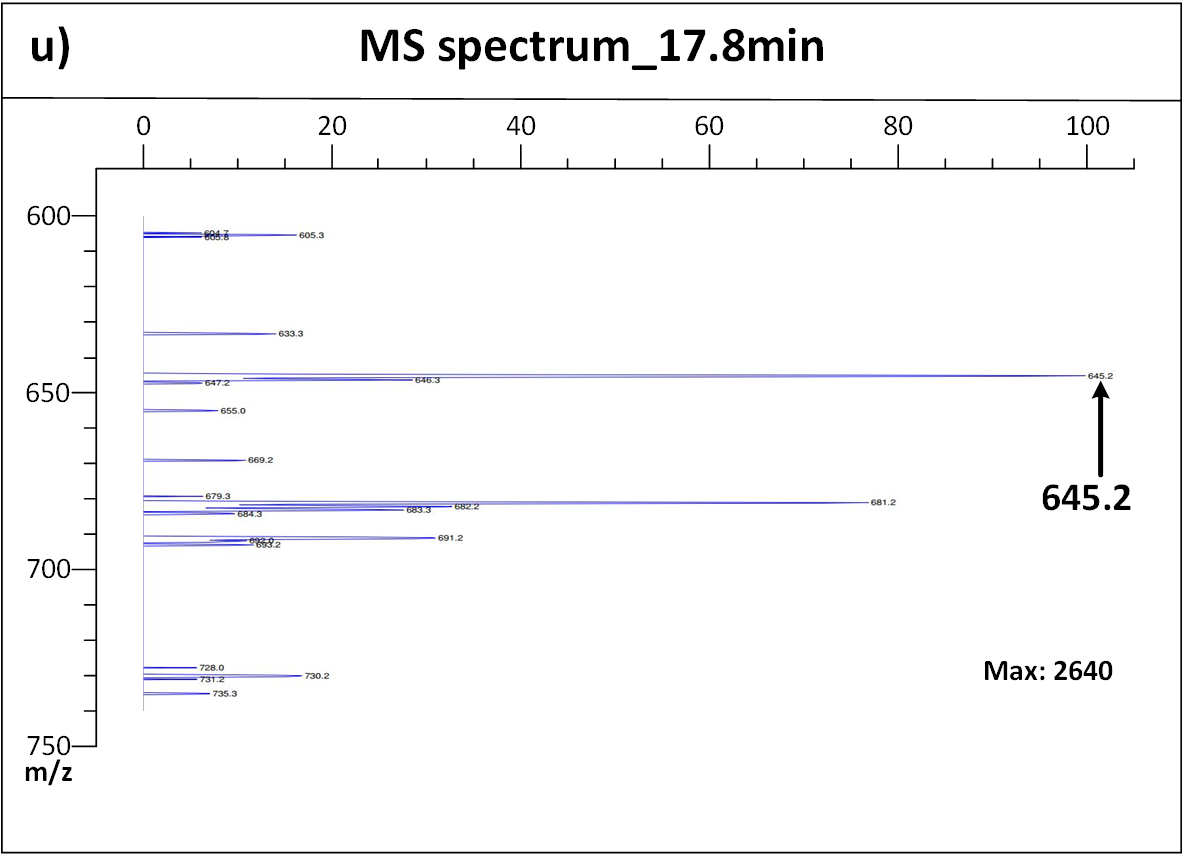

Supplement: Supplementary file 4 — Supplementary Material 4. Figure S4. MS/MS spectra of bola SLs and formed lactonic SLs after enzymatic conversion with rSBLE. [file 13068_2024_2533_MOESM4_ESM.zip › Fig. S4/Fig S4 u).png]

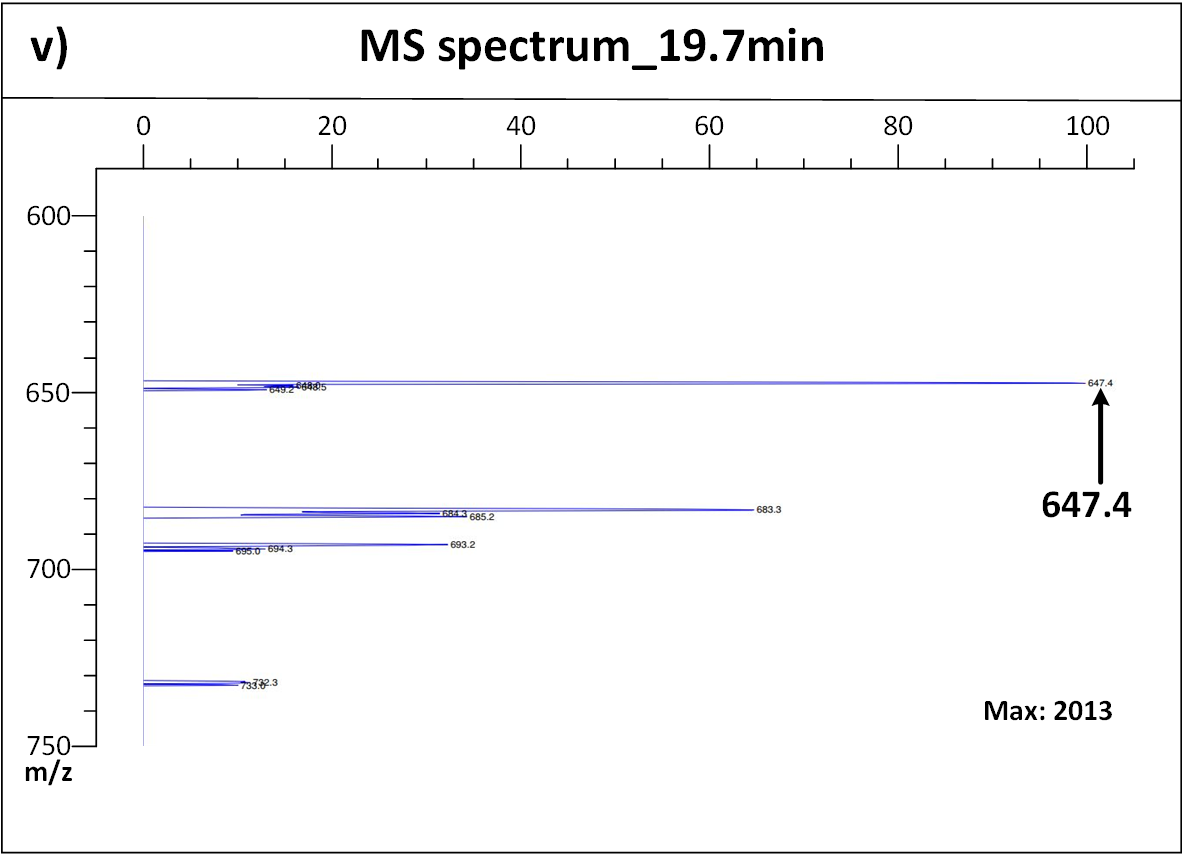

Supplement: Supplementary file 4 — Supplementary Material 4. Figure S4. MS/MS spectra of bola SLs and formed lactonic SLs after enzymatic conversion with rSBLE. [file 13068_2024_2533_MOESM4_ESM.zip › Fig. S4/Fig S4 v).png]

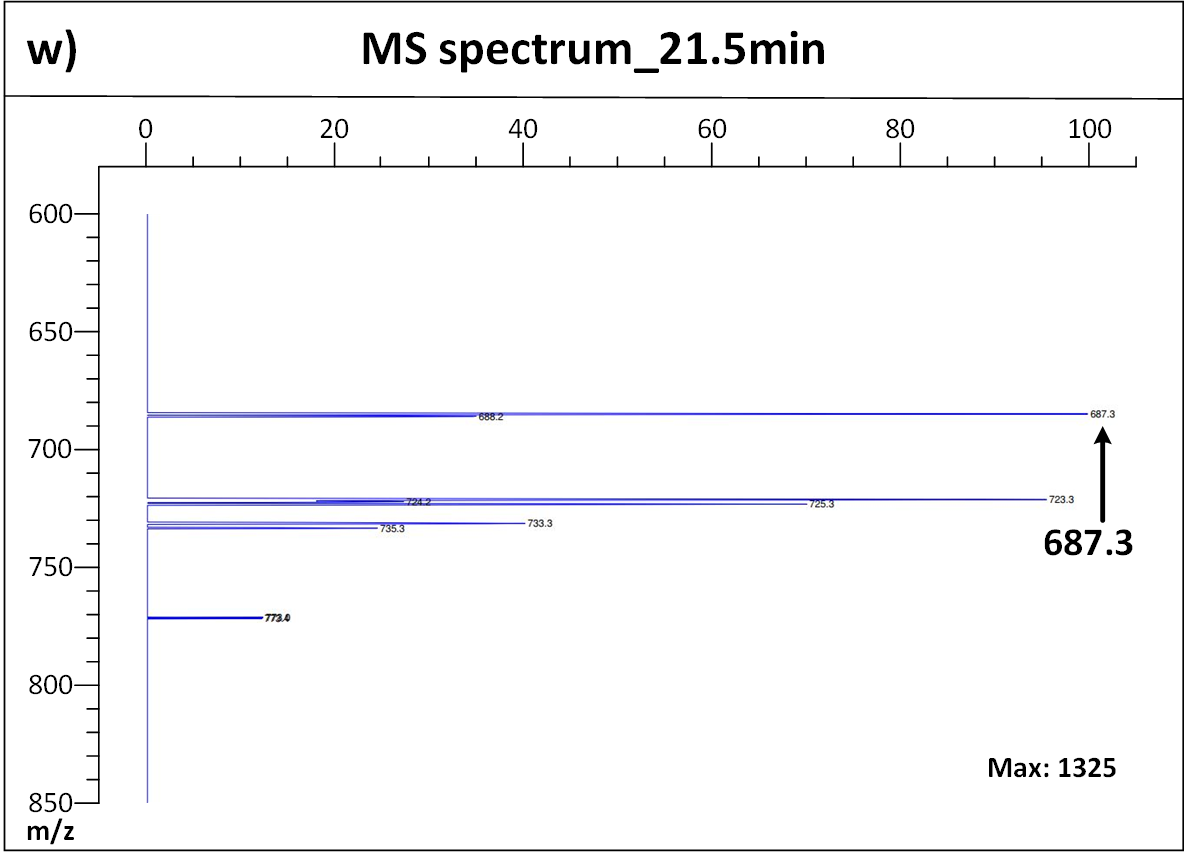

Supplement: Supplementary file 4 — Supplementary Material 4. Figure S4. MS/MS spectra of bola SLs and formed lactonic SLs after enzymatic conversion with rSBLE. [file 13068_2024_2533_MOESM4_ESM.zip › Fig. S4/Fig S4 w).png]

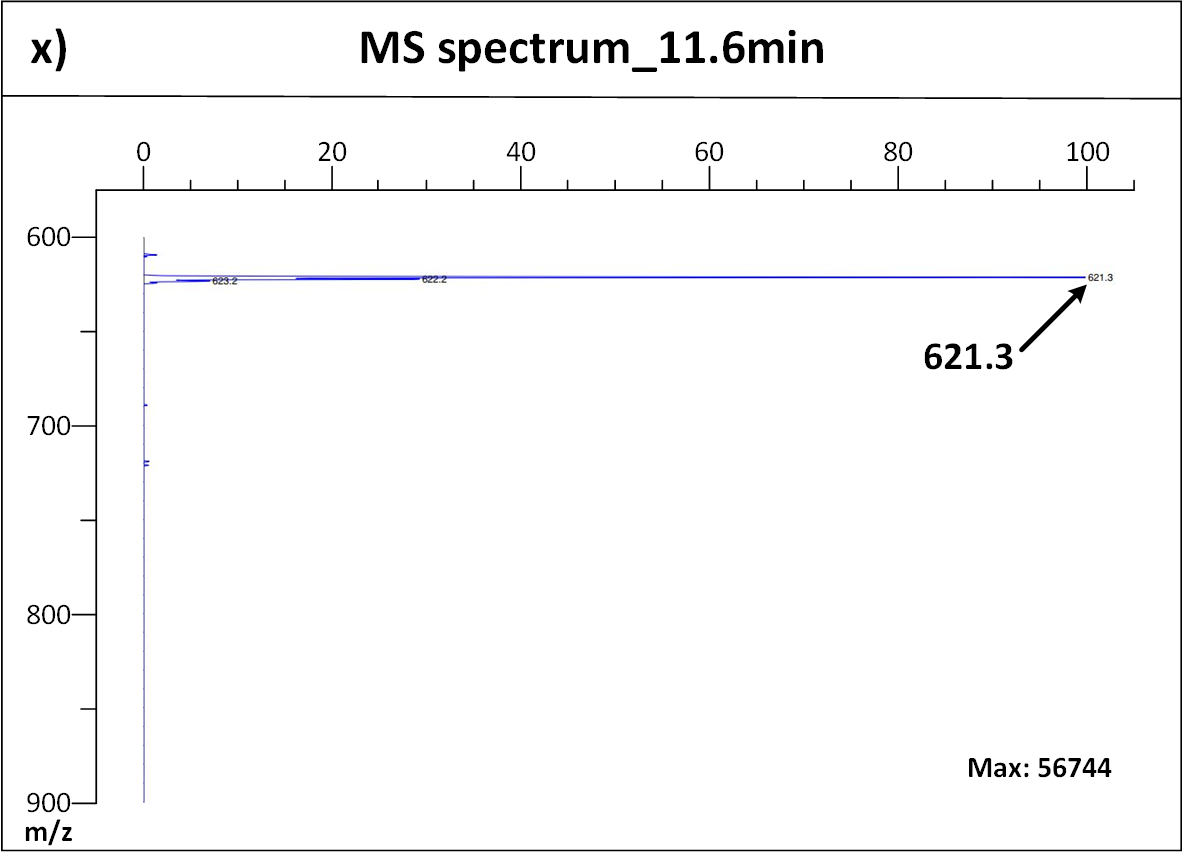

Supplement: Supplementary file 4 — Supplementary Material 4. Figure S4. MS/MS spectra of bola SLs and formed lactonic SLs after enzymatic conversion with rSBLE. [file 13068_2024_2533_MOESM4_ESM.zip › Fig. S4/Fig S4 x).png]

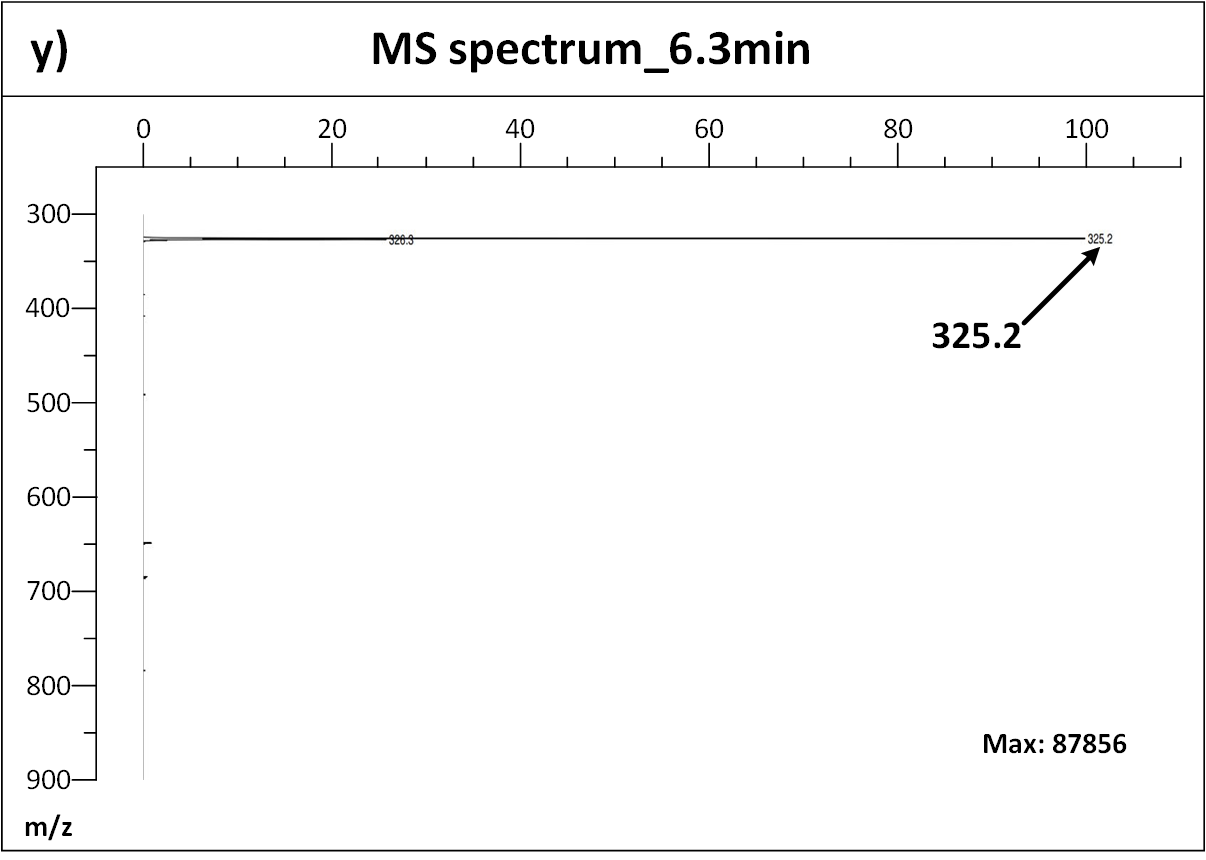

Supplement: Supplementary file 4 — Supplementary Material 4. Figure S4. MS/MS spectra of bola SLs and formed lactonic SLs after enzymatic conversion with rSBLE. [file 13068_2024_2533_MOESM4_ESM.zip › Fig. S4/Fig S4 y).png]

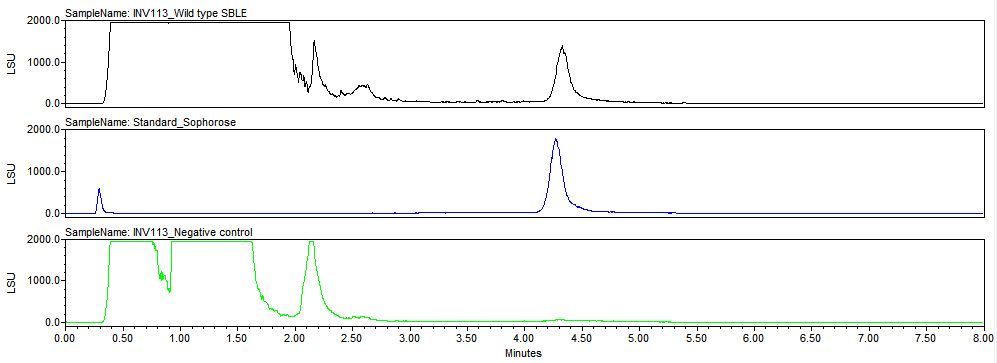

Supplement: Supplementary file 5 — Supplementary Material 5. Figure S5. HPLC analysis of the sophorose released during sophorolipid lactonization. Comparison of retention times with commercial sophorose. [file 13068_2024_2533_MOESM5_ESM.zip › Fig. S5/Fig S5.JPG]
